# Colorimetric indication of hidden catalysis

**DOI:** 10.1038/s41557-025-01955-0

**Published:** 2025-09-16

**Authors:** Julie Macleod, Stephen P. Thomas

**Affiliations:** EaStCHEM School of Chemistry, The University of Edinburgh, Edinburgh, UK

**Keywords:** Catalytic mechanisms, Reaction mechanisms, Inorganic chemistry

## Abstract

Hidden catalysis occurs when an impurity or species generated in situ facilitates the reaction instead of the intended catalyst. This pervasive issue plagues reaction development and fundamental understanding, with prominent examples including trace metal contamination, ‘metal-free’ reactivity, hidden (Brønsted) acid catalysis and hidden borane catalysis. Current methods to identify hidden catalysis are hindered by time-consuming and labour-intensive mechanistic analyses, limiting their practicality and widespread adoption. We introduce a transformative, colorimetric indicator that enables rapid, visual detection of hidden borane catalysis. The colorimetric test was successfully used for reaction sampling, in situ testing, reagent quality control and even test strip analyses. This rapid, visual detection method removes the necessity for laborious and costly mechanistic analyses and offers a powerful tool for chemists to quickly identify and mitigate hidden catalytic effects. This method holds the potential to substantially accelerate discovery and optimization in chemical synthesis by clarifying mechanistic understanding at the outset.

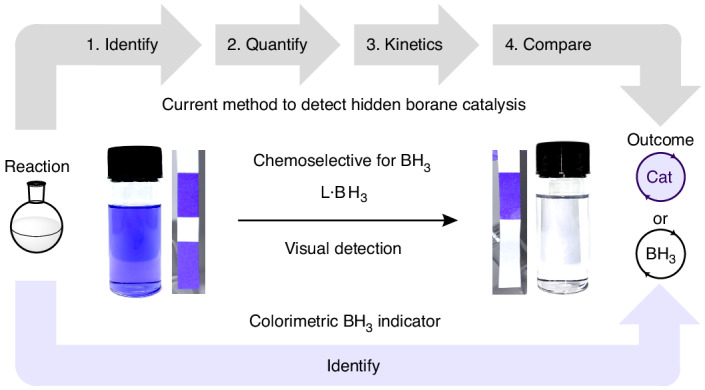

## Abstract (web summary)

Hidden catalysis plagues catalyst development and occurs when an impurity or species generated in situ facilitates the reaction instead of the intended catalyst. Current methods to identify hidden catalysis require time-consuming, labour-intensive mechanistic analyses, so limiting widespread use. A colorimetric indicator has been developed that enables rapid, visual detection of hidden borane catalysis.

## Main

Hidden catalysis, sometimes referred to as Trojan horse catalysis, occurs when an impurity or species generated under reaction conditions acts to facilitate the (target) reaction alongside or instead of the intended (added) catalyst. Hidden catalysis continues to inhibit reaction development and fundamental understanding of catalysis, with notable examples including trace metal contamination^1–6^, ‘metal-free’ reactivity^7,8^, hidden borane^9–17^ and hidden (Brønsted) acid catalysis^18–28^. Possibly the most prevalent of these is hidden borane catalysis; 654 publications on catalysed hydroboration reactions have been reported since 2010, with 67% susceptible to hidden borane catalysis^29^. Many of the proposed ‘catalysts’ used in these reactions simply mediate the decomposition of the dioxaborolane reagent (for example, HBcat **3a** and HBpin **3b**) to catalytically active borane (BH_3_), and derivatives thereof^9–17^. Reactions that proceed exclusively through hidden catalysis offer no improvement in chemo-, regio- or stereoselectivity over commercially available borane reagents and further hinder the development and understanding of novel catalytic processes.

**Fig. 1:**
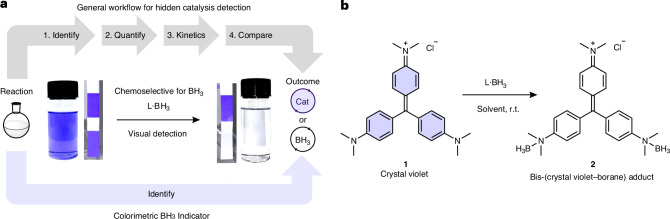
Detecting hidden borane catalysis. **a**, The current procedure (grey) to detect hidden borane catalysis involves four steps: (1) identify the presence of BH_3_ by trapping with TMEDA and observing the adduct by ^11^B NMR spectroscopy; (2) quantify the concentration of BH_3_ generated under the reaction conditions; (3) determine the kinetic viability at the identified concentration of BH_3_; and (4) compare the kinetic data of the reaction conditions to that at the concentration of the hidden BH_3_ to determine which is dominant. The new, one-step, colorimetric method (purple) uses crystal violet **1** (solution or test strips) as an indicator for detecting BH_3_ contamination/formation in hydroboration reactions. This instant, visual detection method removes the need for laborious and costly mechanistic analysis for the detection of hidden BH_3_. **b**, The structure of crystal violet **1** and its colour change upon addition of L·BH_3_ (L = SMe_2_, THF, pyridine, NEt_3_) in various solvents.

Despite this widespread issue, methods to identify all types of hidden catalysis rely on time-consuming, in-depth mechanistic analyses. The state-of-the-art methods to identify hidden catalysis typically comprise: (1) identify the potential for hidden catalysis; (2) identify the hidden catalyst; (3) quantify the amount of the hidden catalyst; (4) determine the kinetic viability of the hidden catalyst at the concentrations identified; and (5) compare the kinetic data of the reaction conditions to that at the concentration of the hidden catalyst to determine which is dominant (Fig. 1a). For hidden acid catalysis this involves identifying and quantifying the hidden acid species using NMR spectroscopy followed by thorough kinetic analyses^18,24^. This is further complicated by complex acids, not simple mineral acids, often being responsible for reactivity; kinetic analyses can therefore result in inconclusive results and extra care and experimentation are needed^18,24^. For trace metal contamination, including ‘metal-free’ reactivity, inductively coupled plasma mass spectrometry analyses of reagents and reaction mixtures are needed to identify trace metal contaminants followed by reaction monitoring to determine the kinetic viability of each of the identified trace metals at the measured level and finally determination of each component’s catalytic potential^1,3,8,30,31^.

The process for identifying hidden borane catalysis is no different, and typically involves the use of *N*,*N*,*N*′,*N*′-tetramethylethylenediamine (TMEDA) to trap any generated BH_3_ (from catalyst-mediated decomposition of HBpin/HBcat) followed by ^1^H and ^11^B NMR spectroscopy and kinetic analyses^9^. Prior to the introduction of the TMEDA method in 2020^9^, only 5% of publications tested for hidden borane catalysis within catalysed hydroboration reactions^29^. Since the introduction of the TMEDA test, 76% of reported catalysed hydroboration reactions were susceptible to hidden borane catalysis, but only 25% of these tested for it^29^. Of those using the inhibition protocol, 56% carried out the test under unsuitable conditions (>60 °C) where the test results are not valid^10^. Testing for hidden borane catalysis therefore remains an unsolved problem due to the requisite experimental and time investment needed to gain accurate identification. A colorimetric test would offer the simplest, low-barrier method to identify hidden borane catalysis and provides a fundamental change in the identification of hidden catalysis in general.

We report a simple, rapid, chemoselective colorimetric indicator for hidden borane catalysis using crystal violet (tris[4-(dimethylamino)phenyl]methylium chloride) **1** (Fig. 1a,b). The colorimetric test was successfully used for reaction sampling, in situ testing, reagent quality control and even test strip analyses.

## Indicator identification

Crystal violet **1** is a common acid–base indicator found in children’s chemistry sets and undergraduate laboratories. A solution of crystal violet **1** in dichloromethane (1.5 × 10^−5^ M) was found to be an intense purple colour (ultraviolet–visible, *λ*_max_ = 590 nm). Addition of excess BH_3_ (10 μl, Me_2_S·BH_3_) resulted in a rapid (<1 min) loss of colour to a colourless solution (ultraviolet–visible, no absorption) due to precipitation of the indicator–borane adduct **2** (Fig. 1b). A control reaction of the addition of SMe_2_ alone to a crystal violet **1** indicator resulted in no colour change, confirming the colour change observed was due to BH_3_. A change to a colourless solution was also observed using other commercially available sources of BH_3_ (L·BH_3_, L = tetrahydrofuran (THF), pyridine, triethylamine (NEt_3_)) and in a range of solvents including THF, dichloromethane, chloroform and chlorobenzene (Supplementary Information, sections 3.3 and 3.4). The detection limit of the crystal violet **1** indicator (0.5 ml, 0.1 mM) was identified as 0.001 M BH_3_ (Supplementary Information, section 3.7). For comparison to hidden borane catalysis, investigations of ‘catalyst’-mediated decomposition of HBpin (7.5 M) generated between 0.01 and 0.3 M BH_3_ in situ^9^. These initial studies indicated crystal violet **1** had the potential to be a colour indicator for hidden borane catalysis.

## Reagent quality control check

1,3,2-Dioxaborolanes, particularly HBcat **3a** and HBpin **3b**, are the go-to hydroboration reagents; however, these reagents rearrange over time to B_2_cat_3_ **3d** and B_2_pin_3_ **3e**, respectively, and BH_3_ (Supplementary Information, scheme 5)^32–34^. We first sought to apply the crystal violet **1** indicator as a reagent quality control check for dioxaborolanes; any reagent decomposition would be confirmed by a colour change from purple to colourless when BH_3_ was present. Control reactions confirmed that solutions of crystal violet **1** did not react with HBcat **3a** or HBpin **3b** with no colour change observed when these reagents were added to the crystal violet **1** indicator (Supplementary Information, section 3.9). When BH_3_ (Me_2_S·BH_3_) was doped into the dioxaborolanes or decomposition deliberately triggered (to produce BH_3_) this was successfully visualized using the crystal violet **1** test; the BH_3_ impurity caused a colour change of the indicator solution from purple to colourless. This showed that the crystal violet **1** indicator served as a rapid quality control test for these widely used reagents and offers a simple route to prevent future false-negative results by reagent contamination. If reagent decomposition has occurred, then the reagent should be purified (Supplementary Information, section 3.10) before use in a hydroboration reaction.

## Control tests; chemoselectivity and reagent compatibility

**Fig. 2:**
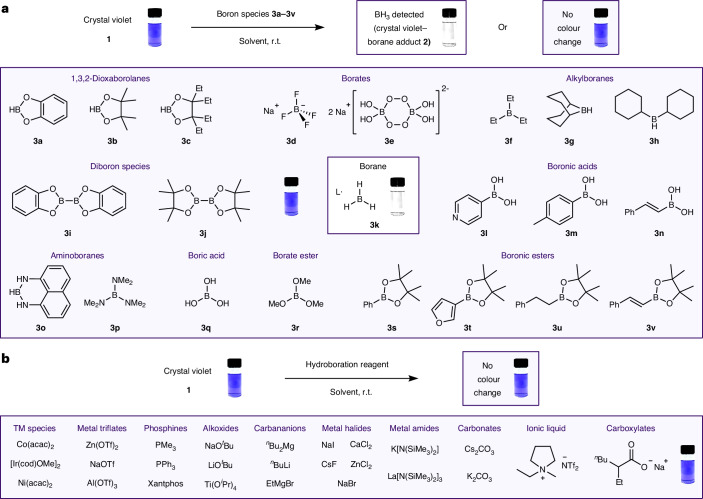
Chemoselectivity and compatibility of the indicator solution. **a**, Chemoselective detection of L·BH_3_ **3k** (L = SMe_2_, THF, pyridine, NEt_3_) was determined by testing the indicator for a colour change in the presence of various organoboron compounds **3a**–**3v**. A colour change from purple to colourless was only observed when BH_3_ **3k** was present. Conditions: boron reagent (0.8 mmol, 1,000 equiv.) and crystal violet solution (4 ml, 0.2 mM, 0.8 µmol, CH_2_Cl_2_). **b**, The compatibility of crystal violet **1** with reagents commonly used in hydroboration was demonstrated by the lack of colour change in the presence of these reagents. Conditions: hydroboration reagent (0.01–0.1 mmol) and crystal violet **1** indicator (2 ml, 0.2 mM, 0.4 µmol, CH_2_Cl_2_ or CHCl_3_). TM, transition metal; Tf, SO_2_CF_3_.

Although we had shown that crystal violet **1** acted as a colorimetric indicator for BH_3_, the general applicability of this test would only be possible if it were selective for BH_3_ over other reagents. Chemoselectivity for BH_3_ detection in the presence of other organoboron species was explored with boronic esters, borate esters, boric acid, boronic acids, amino boranes, borates, diboron species and alkylboranes (Fig. 2a). These all gave no observable colour change when added to the crystal violet **1** indicator, demonstrating excellent chemoselectivity of the indicator for BH_3_ over other organoboron compounds. The wavelength of maximum absorbance (*λ*_max_ = 590 nm) of solutions of crystal violet **1** (1.5 × 10^−5^ M, CH_2_Cl_2_) remained unchanged in the presence of trimethyl borate **3k**, boronic ester **3o** or sodium tetrafluoroborate (Supplementary Information, section 3.12).

Alongside organoborane reagents, chemoselectivity in the presence of typical catalyst structures was also explored. It is essential that the indicator colour change should not triggered by common structural motifs within catalysts for it to be used a test for hidden borane catalysis. Addition of metal halides, metal amides, metal triflates, transition metal species, carbonates, carboxylates, carbanions, alkoxides, ionic liquids, phosphines and amines to the crystal violet **1** indicator again gave no observable colour change (Fig. 2b), thereby demonstrating chemoselectivity for BH_3_ over all these typical catalyst motifs (Supplementary Information, section 3.13*)*. A broad series of reagents were tested with crystal violet **1** indicator solution to confirm chemoselective indication of BH_3_ over direct reaction with crystal violet. It is recommended that all reagents/catalysts are tested separately with crystal violet **1** (in situ method) before drawing a conclusion on the test result for the full catalytic conditions.

## Overcoming limitations

**Fig. 3:**
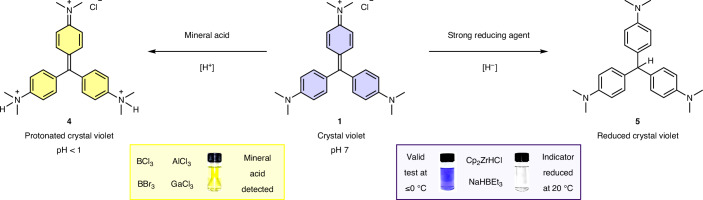
Limitations of the crystal violet indicator. The crystal violet **1** indicator turns yellow under acidic conditions (pH < 1). Main group halides (BF_3_, BCl_3_, BBr_3_, AlCl_3_, GaCl_3_), which readily hydrolyse to form mineral acid in the presence of trace water, therefore result in a colour change from purple to yellow being observed. Strong reducing agents react with crystal violet **1**, resulting in the formation of a colourless, reduced form **5**. This can be avoided by cooling, and testing the indicator solution at 0 °C.

Two limitations were identified: mineral acids and strong reducing agents (Fig. 3). Strong reducing agents (for example, Schwartz reagent and sodium triethylborohydride) react with the indicator to form its colourless, reduced derivative **5** (Supplementary Information, Section 3.15). This was avoided by carrying out the indicator test at 0 °C (crystal violet **1** indicator cooled with an ice bath), presumably by slowing the rate of reduction. Importantly decolorization by BH_3_ at 0 °C was still observed, therefore validating the hidden BH_3_ test at this reduced temperature.

In the presence of acid (HCl, 1.1 ml, 1 M, pH 1), the crystal violet **1** indicator solution (10 ml, 1.5 × 10^−5^ M) was observed to change colour from purple to yellow, not colourless (Supplementary Information, section 3.16). This was also observed when main group halides were tested; where any water was present (non-anhydrous conditions or advantageous water) a colour change to yellow, rather than colourless, was observed, indicating acid formation (by hydrolysis of the main group halide).

## Detecting ‘catalyst’-mediated HBpin decomposition

**Fig. 4:**
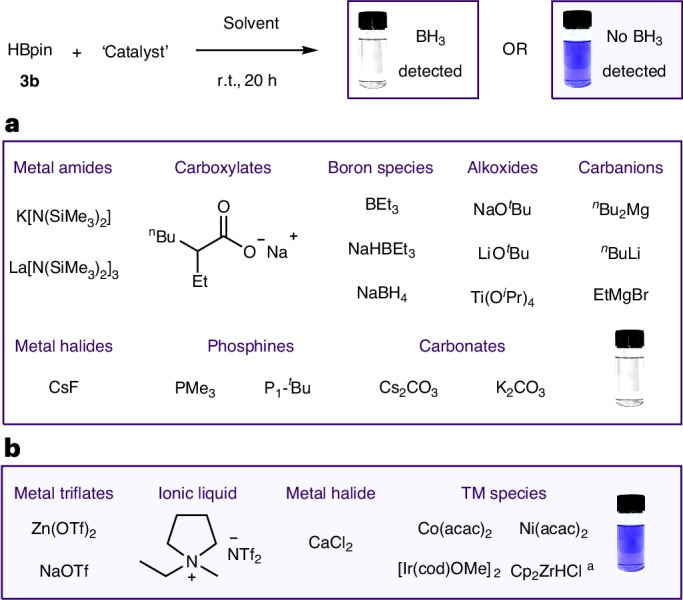
Testing ‘catalysts’ for in situ generation of BH_3_ by ‘catalyst’-mediated decomposition of HBpin. **a**, ‘Catalyst’-mediated decomposition of HBpin **3b** to BH_3_ was observed. **b**, No BH_3_ generated. Conditions: HBpin **3b** (3–5 mmol), ‘catalyst’ (0.01–0.1 mmol), solvent (0.2–1 ml, THF, toluene, dichloromethane or neat) and crystal violet **1** indicator (2 ml, 0.2 mM) used in situ or ex situ. ^a^Schwartz’s reagent (Cp_2_ZrHCl) was tested at 0 °C.

The ability to selectively detect BH_3_ in reagents, additives and catalysts independently is highly valuable, but this does not mimic catalysed hydroboration reaction conditions in which a catalyst is reacted in the presence of the hydroboration reagent. We thus sought to test if the crystal violet **1** indicator could be applied to detect BH_3_ formed in situ from ‘catalyst’-mediated HBpin **3b** decomposition. Catalyst structures previously used in hydroboration reactions were added to solutions of HBpin **3b** and the crystal violet **1** indicator used to identify catalyst-mediated decomposition of HBpin **3b** to BH_3_ (Supplementary Information, section 3.17). All reactions were tested at room temperature to mitigate any thermal decomposition of HBpin. Schwartz’s reagent (Cp_2_ZrHCl) was tested at 0 °C to avoid reduction of the indicator. Both in situ testing (crystal violet **1** indicator added directly to the reaction mixture) and ex situ testing (sample of the reaction mixture added to the crystal violet **1** indicator) successfully detected BH_3_ and hidden catalysis by a colour change from purple to colourless. Where reaction mixtures were strongly coloured, visualization of the colour change (purple to colourless) was most easily observed using the ex situ method. All tests were validated by ^11^B NMR spectroscopy, which confirmed the formation of BH_3_. Metal alkoxides, metal amides, metal carbanions, carbonates and boron species were among the reagents tested that mediated the decomposition of HBpin **3b** to BH_3_, resulting in a colourless indicator solution (Fig. 4a) and successful detection of hidden catalysis. Importantly where no BH_3_ was generated, for example, using metal trilfates and Schwartz’s reagent, the indicator remained purple (Fig. 4b), demonstrating that these species could be used as ‘true’ catalysts.

## Detecting hidden catalysis under full reaction conditions

**Fig. 5:**
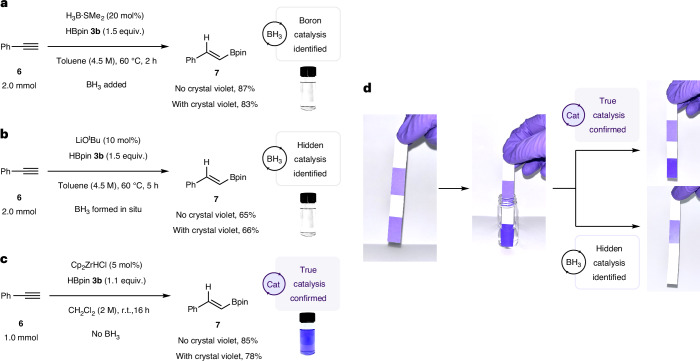
Visual detection of BH_3_ under full reaction conditions. **a**, BH_3_-catalysed hydroboration was used as the control reaction; the indicator solution changed from purple to colourless. Conditions: phenylacetylene **6** (2.0 mmol), pinacolborane (3.0 mmol), borane-dimethyl sulfide (0.4 mmol, 20 mol% BH_3_) and toluene (0.2 ml, 4.5 M) ± crystal violet **1** indicator (0.048 μM). **b**, BH_3_ generated in situ by LiO^*t*^Bu-mediated decomposition of HBpin; the crystal violet **1** indicator solution changed from purple to colourless due to the presence of BH_3_ in the reaction. Hidden borane catalysis was successfully identified. Conditions: phenylacetylene **6** (2.0 mmol), pinacolborane (3.0 mmol), LiO^*t*^Bu (0.2 mmol, 10 mol%) and toluene (0.2 ml, 4.5 M). **c**, Schwartz’s reagent does not result in the decomposition HBpin to BH_3_ and therefore no colour change was observed with the crystal violet **1** indicator, confirming true catalysis. Conditions: phenylacetylene **6** (1.0 mmol), pinacolborane (1.1 mmol) Schwartz’s reagent (0.5 mmol, 5.0 mol%) and dichloromethane (0.5 ml, 2 M). **d**, Alternatively, test strips can be used to identify the presence of BH_3_ in reagents and/or reaction conditions. No colour change was observed when the indicator strips were dipped into a solution with no BH_3_, confirming true catalysis. A colour change from purple to white indicates the presence of BH_3_, confirming hidden catalysis.

Having demonstrated the broad and chemoselective application of the crystal violet **1** indicator to reagents, catalysts and in situ dioxaborolane decomposition, we subsequently investigated testing full reaction mixtures. Unlike our previous detection method which involved time-consuming kinetic analyses, we hypothesized that this new colorimetric approach would offer a direct, simple and broadly applicable method to detect hidden borane catalysis using a single sample of an entire reaction mixture. This would be a visual indicator of hidden catalysis that does not require kinetic analyses. A control reaction using the BH_3_-catalysed hydroboration of phenylacetylene **6** with HBpin **3b** confirmed that the crystal violet **1** indicator decolorized using entire reaction mixtures both by in situ and ex situ testing^35^. The key colour change from purple to colourless was observed, confirming BH_3_ catalysis (Fig. 5a). The crystal violet **1** indicator gave the same visual indication when added to the reaction (in situ testing) or when reaction samples were taken and added to the crystal violet **1** indicator (ex situ testing). When LiO^*t*^Bu was used to deliberately trigger hidden BH_3_ catalysis^9^, by in situ decomposition of HBpin **3b**, in situ and ex situ testing was again successful, showing the direct, visual detection of hidden catalysis (Fig. 5b). It should be noted that indication of hidden BH_3_ catalysis by crystal violet **1** did not stop catalysis with product formation still being observed after in situ testing (Supplementary Information, section 3.18). This enabled not only the identification of ‘hidden’ catalysis, but also ‘true’ catalysis. Confirmation of true catalysis was carried using Schwartz’s reagent as the ‘true’ catalyst (one that does not decompose HBpin **3b** to BH_3_)^36^ where no colour change of the indicator was observed using in situ or ex situ testing (Fig. 5c).

In analogy to litmus paper, crystal violet **1** indicator paper was prepared (Supplementary Information, section 3.23) and used as a ‘dip stick’ method, greatly increasing the convenience of this ‘hidden’ borane catalysis indicator (Fig. 5d). Indicator strips (stained with crystal violet **1** solution) were dipped into a solution of BH_3_ (1 mM, in dichloromethane) and lost the purple colour, giving a white strip within 30 s. When no BH_3_ was present, no colour change of the indicator strip was observed, an indication of ‘true’ catalysis. This method was also applied to in situ testing of entire reaction mixtures with equal success (Supplementary Table 4).

## Improving on the state-of-the-art TMEDA test method

The current gold standard method to identify hidden borane catalysis is based on TMEDA sequestering BH_3_ from solution^9^; however, this test fails above 60 °C^10^. When crystal violet **1** was used as a BH_3_ colour indicator, it successfully identified hidden catalysis for reactions at 80 °C where the TMEDA method has been shown to fail (Table 1). This further exemplifies the advantage of using the crystal violet **1** indicator for visual detection over the current state-of-the-art.

**Table 1:** TMEDA inhibition versus crystal violet **1** indicator to detect hidden BH_3_ catalysis for the hydroboration of phenylacetylene **6** with HBpin **3b** (‘catalyst’-mediated decomposition of HBpin **3b** to BH_3_)


Entry	‘Catalyst’	TMEDA inhibition method				Crystal violet indicator		
		Control yield (%)	Yield in the presence of TMEDA (%)		Hidden catalysis identified	Colour change^c^ (at 80 °C)		Hidden catalysis identified
		60 °C	60 °C	80 °C	(Yes/No)	(Yes/No)		(Yes/No)
1	NaOH	41	1	7	Yes	Yes		Yes
2	NaO^*t*^Bu	59^a^	1^a^	77^a^	No	Yes		Yes
3	Na[N(SiMe_3_)_2_]	29	0	49	No	Yes		Yes
4	^*n*^BuLi	52	1^b^	27^b^	No	Yes		Yes
5	Ti(O^*i*^Pr)_4_	51	5^b^	39^b^	No	Yes		Yes

Conditions: phenylacetylene **6** (1.0 mmol), ‘catalyst’ (0.10 mmol), HBpin **3b** (1.5 mmol) ± TMEDA (0.10 mmol). Yields were determined by ^1^H NMR spectroscopy using an internal standard (1,3,5-trimethoxybenzene). ^a^18 h. ^b^With 0.30 mmol of TMEDA. ^c^Tested by taking a sample and adding this to the crystal violet **1** indicator or by adding an aliquot of crystal violet **1** indicator to the reaction vial.

## Conclusion

‘Hidden’ catalysis limits not only catalyst and reaction development, but also mechanistic understanding and application to large-scale settings. The state-of-the-art methods to identify ‘hidden’ from ‘true’ catalysis requires extensive effort and kinetic analyses, and thus routine adoption has yet to be realized. Catalysed hydroboration reactions are no exception to this and are plagued by hidden borane catalysis. The majority of new catalytic hydroboration systems contain nucleophilic motifs that are capable of decomposing HBcat **3a** and HBpin **3b** to generate catalytically active BH_3_ and so susceptible to hidden borane catalysis. Crystal violet **1**, a bench-stable and commercially available reagent, has been developed as a colorimetric indicator for ‘hidden’ borane catalysis. A colour change from purple to colourless indicated the presence of BH_3_ and thus the hidden borane catalysis for species from across the periodic table. Crystal violet **1** was found to be a chemoselective indicator for BH_3_ across other organoboron species, hydroboration substrates, HBcat **3a** and HBpin **3b**, nucleophilic species and all the ‘catalysts’ tested. The crystal violet **1** indicator also successfully indicated BH_3_ generated by nucleophilic- and ‘catalyst’-mediated decomposition of HBpin **3b**. Because the crystal violet **1** indicator did not inhibit catalysis, in situ testing was possible to identify ‘hidden’ and ‘true’ catalysis using entire reaction mixtures. Indicator test strips were prepared as a ‘litmus test’ to make detection of this complex catalytic phenomenon even easier and to showcase the simplicity and applicability of the method, and its potential for commercial distribution. This indicator offers a simple and easily applied test for all future catalysed hydroboration reactions. This qualitative method to indicate a ‘hidden’ catalysis manifold offers a step-change in catalysis development and understanding, making the identification of ‘hidden’ borane catalysis routine.

## Online content

Any methods, additional references, Nature Portfolio reporting summaries, source data, extended data, supplementary information, acknowledgements, peer review information; details of author contributions and competing interests; and statements of data and code availability are available at 10.1038/s41557-025-01955-0.

## Supplementary information

Supplementary Information Supplementary Figs. 1–64, Tables 1–4, Schemes 1–11, discussions and images, experimental data, characterization data and NMR spectra.

## Data Availability

All data are available in the main text or Supplementary Information.
