# Supplementary material for: Colorimetric indication of hidden catalysis
Source: Nat Chem. 2025 Sep 16;18(1):173–9. doi: 10.1038/s41557-025-01955-0 (PMC12768962; doi:10.1038/s41557-025-01955-0)
Supplement: Supplementary file 1 — Supplementary Figs. 1–64, Tables 1–4, Schemes 1–11, discussions and images, experimental data, characterization data and NMR spectra. [file 41557_2025_1955_MOESM1_ESM.pdf]

nature chemistry

Supplementary information https://doi.org/10.1038/s41557-025-01955-0

Colorimetric indication of hidden catalysis

In the format provided by the authors and unedited

Table of Contents

1. General Experimental ... 3

2. Proposed Mechanism for Nucleophilic Decomposition of Pinacolborane ... 4

3. Experimental Procedures ... 4

3.1. Method to Prepare the Crystal Violet Indicator Solution ... 4

3.2. Initial Tests for Crystal Violet as a Colour Indicator ... 4

3.3. Suitable Solvents for the Crystal Violet Indicator ... 5

3.4. Testing Other Commercially Available Boranes ... 6

3.5. UV-Vis Analysis of Crystal Violet Indicator ... 6

3.6. Characterisation of Crystal Violet Indicator ... 7

3.6.1. Characterisation of Solution ... 7

3.6.2. Characterisation of Precipitate ... 9

3.7. Detection Limit of Crystal Violet Indicator ... 11

3.7.1. Using a 0.1 mM Indicator Solution ... 11

3.7.2. Using a 0.01 mM Indicator Solution ... 12

3.7.3. Using a 0.001 mM Indicator Solution ... 12

3.8. Synthesis of 1,3,2-Dioxaborolanes ... 13

3.8.1. Preparation of Catecholborane (HBcat) ... 13

3.8.2. Preparation of 4,4,5,5-Tetraethyl-1,3,2-dioxaborolane (HB(Epin)) ... 13

3.9. Testing 1,3,2-Dioxaborolanes with Crystal Violet Indicator ... 13

3.10. Purification Method for 1,3,2-Dioxaborolanes ... 13

3.11. Thermal Stability of 1,3,2-Dioxaborolanes ... 15

3.12. Testing Other Boron Species with Crystal Violet (Chemoselectivity) ... 17

3.13. Testing Common Hydroboration Reagents with Crystal Violet 19

3.14. Adduct Competition Between Crystal Violet and Other Amines 20

3.15. Testing Strong Reducing Agents with Crystal Violet 21

3.16. Crystal Violet and Brønsted Acids 22

3.16.1. Crystal Violet as a Brønsted Acid Indicator 22

3.16.2. Crystal Violet Indicator with Main Group Halides 24

3.17. Detecting ‘Catalyst’-mediated Dioxaborolane (HBpin) Decomposition.................... 24

3.18. Hydroboration in the Presence of Crystal Violet 27

3.19. Hydroboration Catalysed by Crystal Violet-Borane Complex.................................. 29

3.20. Hidden BH_3 Detection – Full Reaction Conditions 30

3.21. True Catalysis Example – Schwartz’s Reagent 30

3.22. Comparison to TMEDA Inhibition Method 31

3.23. Crystal Violet Indicator Strips 32

3.23.1. Preparation of the Test Strips 32

3.23.2. Using the Test Strips to Detect Hidden Borane Catalysis................................. 33

3.24. Using TRIGIT for Colourimetric Analysis 34

3.25. Other Indicators 35

4. References 36

5. NMR Spectra 37

For Videos, see https://www.youtube.com/@spthomasgroup

1. General Experimental

Reaction Setup: All reactions were performed in oven (180 °C) dried glassware under an atmosphere of anhydrous argon or nitrogen, unless otherwise indicated. All air- and moisture-sensitive reactions were carried out using standard vacuum line and Schlenk techniques, or in a glovebox with a purified argon atmosphere. All reported reaction temperatures correspond to external bath temperatures. Room temperature (rt) was approximately 18 °C.

NMR Spectroscopy: ^{1}H, ^{13}C and ^{11}B NMR spectra were recorded on Bruker Avance III 400 and 500 MHz; Bruker PRO 500 MHz; Bruker Avance I 600 MHz spectrometers. Chemical shifts are reported in parts per million (ppm) and referenced to tetramethylsilane for ^{1}H and ^{13}C NMR spectra and $Et_2O{\cdot}BF_3$ for ^{11}B NMR spectra. ^{1}H and ^{13}C NMR spectra were referenced to the residual solvent peak ($CHCl_3$: ^{1}H 7.26 ppm, ^{13}C 77.00 ppm). Multiplicities are indicated by br. (broad), s (singlet), d (doublet), t (triplet), q (quartet), quin. (quintet), sext. (sextet), sept. (septet), m. (multiplet). Coupling constants, *J*, are reported in Hertz and rounded to the nearest 0.1 Hz. A background suppression function was applied to all ^{11}B NMR spectra.

Infrared Spectroscopy: Infrared (IR) spectra were recorded on Shimadzu IR-Spirit Spectrometer.

UV-Vis Spectroscopy: Shimadzu UV-1900 UV-Vis Spectrophotometer was used for UV-Vis analyses. Absorbance was recorded between 250 nm – 650 nm.

Solvents: All solvents for air- and moisture-sensitive techniques were obtained from an anhydrous solvent system (Innovative Technology). Dichloromethane (CH_2Cl_2) (Fisher, unstabilized HPLC grade) were dried by percolation through two columns packed with neutral alumina under a positive pressure of argon. Toluene (ACS grade) was dried by percolation through a column packed with neutral alumina and a column packed with Q5 reactant (supported copper catalyst for scavenging oxygen) under a positive pressure of argon. Deuterated chloroform, $CDCl_3$, (Sigma Aldrich) was dried over molecular sieves and stored under an argon atmosphere. Chloroform, $CHCl_3$, (Fisher Scientific UK); Dichloromethane, CH_2Cl_2, (Honeywell); Ethyl Acetate, EtOAc, (Sigma Aldrich); Acetone, $(CH_3)_2CO$, (Fisher Scientific UK), Tetrahydrofuran, THF, (Fisher Scientific UK); Chlorobenzene, PhCl, (Fisher Scientific UK); and Toluene, PhMe, (Fisher Scientific UK) were used as received. Solvents were dried if they were to be used for *in situ* testing (i.e. when the indicator solution was added into reaction).

Chemicals: All reagents were purchased from Sigma Aldrich, Alfa Aesar, Acros Organics, Fisher Scientific UK, Honeywell and Fluorochem and used without purification.

2. Proposed Mechanism for Nucleophilic Decomposition of Pinacolborane

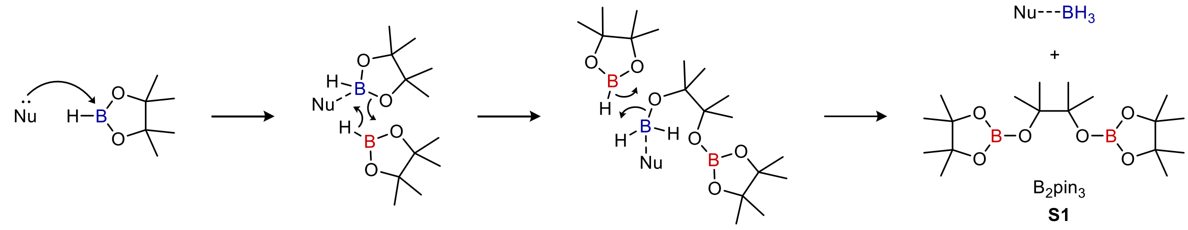

Scheme 1: Proposed mechanism for the nucleophilic decomposition of pinacolborane (based on the analogues mechanism proposed for catecholborane) (*32* - *34*).

3. Experimental Procedures

3.1. Method to Prepare the Crystal Violet Indicator Solution

The crystal violet indicators were prepared by adding crystal violet **1** to a compatible solvent until an intense purple colour was visible (approximately 0.5 – 1 mg per 10 mL of solvent, 0.1 – 0.2 mM) – see Fig. 1. Alternatively, a stock solution was prepared using crystal violet **1** (10 mg, 0.025 mmol) in a suitable solvent (125 mL, 0.20 mM) followed by serial dilutions to reach lower concentrations (0.1 mM or 0.01 mM). For suitable solvents, see *section S3.3 Suitable Solvents for the Crystal Violet Indicator.* For preparing *ex-situ* indicator solutions (sample taken from the reaction and added into indicator solution), solvents can be used as received. For *in-situ* indicator solutions (indicator solution added into the reaction) anhydrous solvents are recommended for preparing the indicator solution.

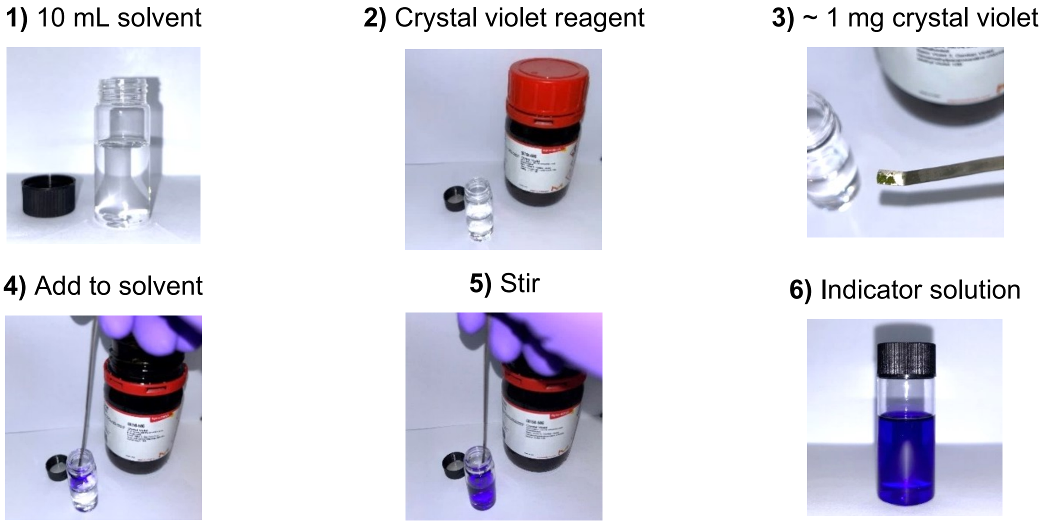

Fig. 1: Visualisation of procedure to prepare crystal violet indicator solution.

3.2. Initial Tests for Crystal Violet as a Colour Indicator

$Me_2S·BH_3$ (0.20 mL, 2.1 mmol) was added to a solution of crystal violet **1** in CH_2Cl_2 (4 mL, ~0.2 mM) and a colour change from purple to colourless was observed (Scheme 2A). As a control, SMe_2 (0.20 mL, 2.7 mmol) was also tested with the crystal violet **1** solution (4 mL, ~0.2 mM solution) and no colour change was observed (Scheme 2B).

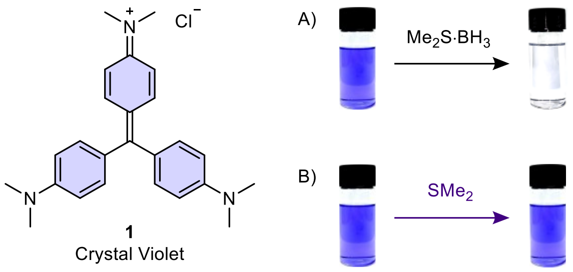

Scheme 2: **(A)** Change in colour of the crystal violet **1** solution from purple to colourless after addition of $Me_2S{\cdot}BH_3$. **(B)** No colour change observed for the control test of SMe_2 with the crystal violet **1** solution.

3.3. Suitable Solvents for the Crystal Violet Indicator

Crystal violet **1** was added to various solvents to test its solubility. The solvents investigated were ethyl acetate, acetone, tetrahydrofuran, dichloromethane, chloroform, chlorobenzene and toluene (Fig. 2A). Crystal violet **1** (~0.5 mg, 1 µmol) was fully soluble in tetrahydrofuran, dichloromethane, chloroform and chlorobenzene (5 mL, 0.2 mM) and a colourless solution was observed upon addition of $Me_2S{\cdot}BH_3$ (0.1 mL, 1 mmol). Crystal violet **1** was fully soluble in acetone and a clear colour change was observed upon addition of $Me_2S{\cdot}BH_3$ (0.1 mL, 1 mmol) but a slight purple hue remained. Crystal violet **1** (1 mg, 2 µmol) was partially soluble in ethyl acetate (5 mL) however a clear colour change was observed upon addition of $Me_2S{\cdot}BH_3$ (0.1 mL, 1 mmol). Crystal violet **1** (8 mg, 20 µmol) had poor solubility in toluene (5 mL) but a clear colour change is observable in the presence of $Me_2S{\cdot}BH_3$ (0.1 mL, 1 mmol), note that the crystal violet-borane precipitate **2** is visible. Overall, the most compatible solvents were tetrahydrofuran, dichloromethane, chloroform and chlorobenzene. Toluene can be used if a non-polar solvent is required.

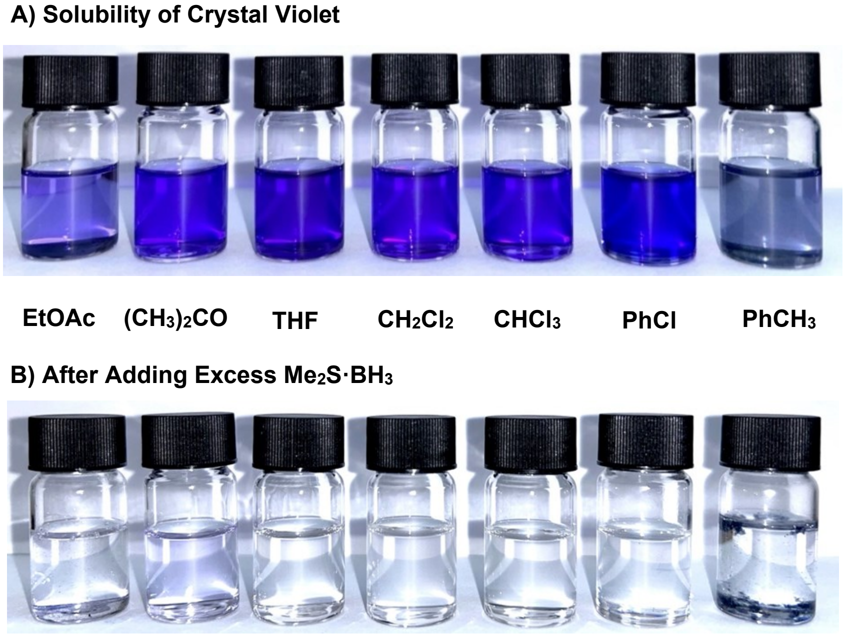

Fig. 2: **(A)** Testing the solubility of crystal violet **1** in various solvents. **(B)** Colour change after the addition of $Me_2S{\cdot}BH_3$.

3.4. Testing Other Commercially Available Boranes

Other commercially available sources of BH_3 were tested with crystal violet **1** solutions in either CH_2Cl_2 or THF (Fig. 3). THF-borane complex **S2** (0.20 mL, 2.0 mmol) was added to the crystal violet **1** indicator (2 mL, 0.2 mM, THF) and resulted in a colourless solution. When pyridine-borane complex **S3** (0.20 mL, 2.0 mmol) was added to the crystal violet **1** indicator solution (2 mL, 0.2 mM, THF), a colour change from purple to colourless was observed with respect to the indicator; the pyridine borane complex was a light red/brown which resulted in the slight pink colour observed. *tert*-Butyldimethyl phosphine-borane complex **S4** (30 mg, 2.0 mmol) was added to the crystal violet **1** indicator (2 mL, 0.2 mM, CH_2Cl_2) and resulted in no colour change, presumably due to stronger Lewis acid-Lewis base complex of phosphine-borane adduct compared to that of the amine-borane adduct. Triethylamine-borane complex **S5** (0.20 mL, 2.2 mmol) was added to the crystal violet **1** indicator (2 mL, 0.2 mM, CH_2Cl_2) and resulted in a significant colour change but with a slight purple hue remaining.

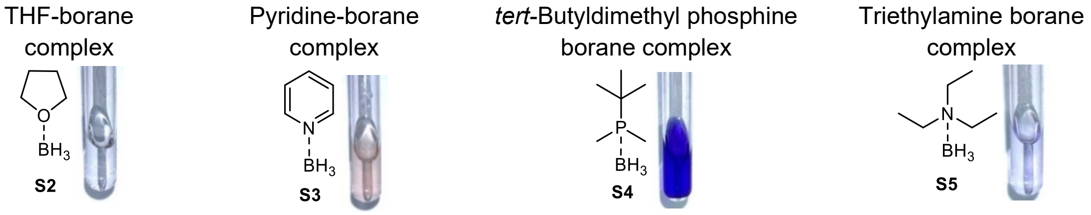

Fig. 3: Testing other commercially available sources of $L{\cdot}BH_3$ with the crystal violet **1** indicator.

3.5. UV-Vis Analysis of Crystal Violet Indicator

A solution of crystal violet **1** (1.5×10^{-5} M, CH_2Cl_2) was prepared through serial dilutions under an inert argon atmosphere. A Schlenk cuvette was used to allow for analyses to be carried out under an argon atmosphere (Fig. 4). The UV-Vis spectrum was recorded before and after addition of $Me_2S{\cdot}BH_3$ (10 µL) (Fig. 5). The wavelength at maximum absorbance, λ_{max}, was 590 nm before the addition of $Me_2S{\cdot}BH_3$. No absorbance was observed after the addition of $Me_2S{\cdot}BH_3$, confirming that the colourless solution is a result of precipitation of the bis(crystal violet-borane) adduct **2**.

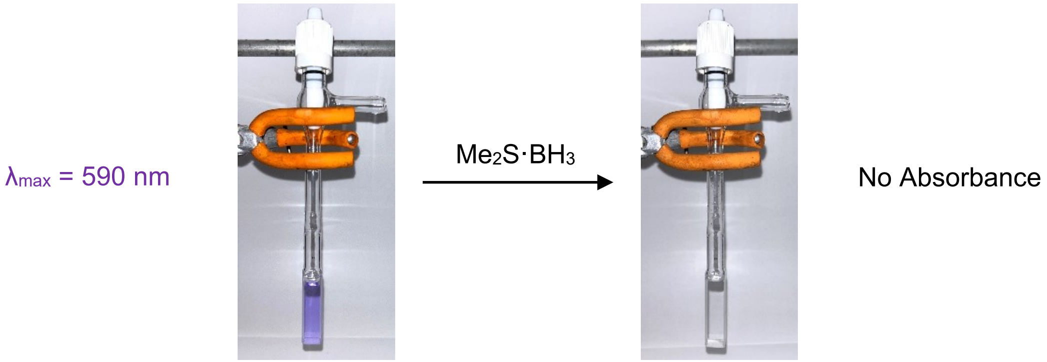

Fig. 4: Crystal violet **1** solution (1.5×10^{-5} M, CH_2Cl_2) in Schlenk cuvette before and after addition of $Me_2S{\cdot}BH_3$.

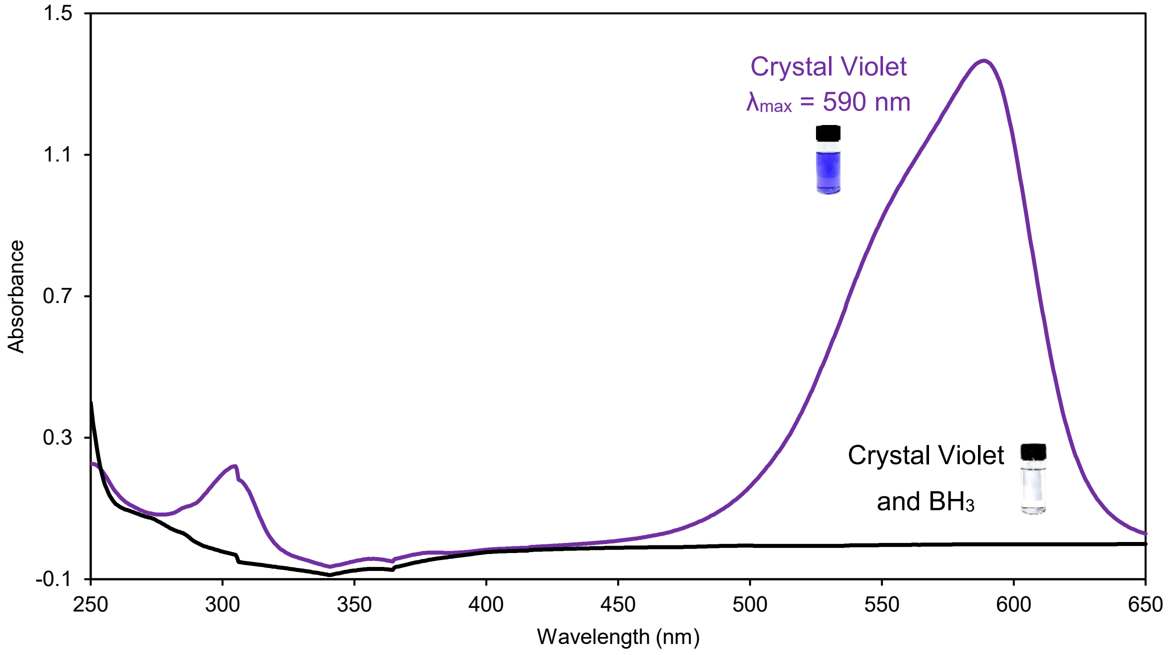

Fig. 5: UV-Vis absorption spectra of the crystal violet **1** solution (1.5 x 10^{-5} M, CH_2Cl_2) before and after addition of $Me_2S{\cdot}BH_3$.

3.6. Characterisation of Crystal Violet Indicator

3.6.1. Characterisation of Solution

Under a N_2 atmosphere, crystal violet **1** (8 mg, 0.02 mmol) was dissolved in $CDCl_3$ (0.5 mL) then borane dimethyl sulfide complex (4 uL, 0.04 mmol) was added. ^{1}H and ^{11}B NMR spectra were recorded.

^{1}H NMR: (400 MHz, $CDCl_3$)

Bis-Adduct **2:** 7.53 (m, 6H), 7.06 (m, 6H), 3.07 (s, 3H)

Mono-Adduct **S6:** 7.41 (m, 6H), 7.09 (m, 6H), 3.03 (s, 3H)

^{11}B NMR: (128 MHz, $CDCl_3$)

Bis-Adduct **2:** 6.50 (br. s)

Mono-Adduct **S6:** 1.21 (br. s)

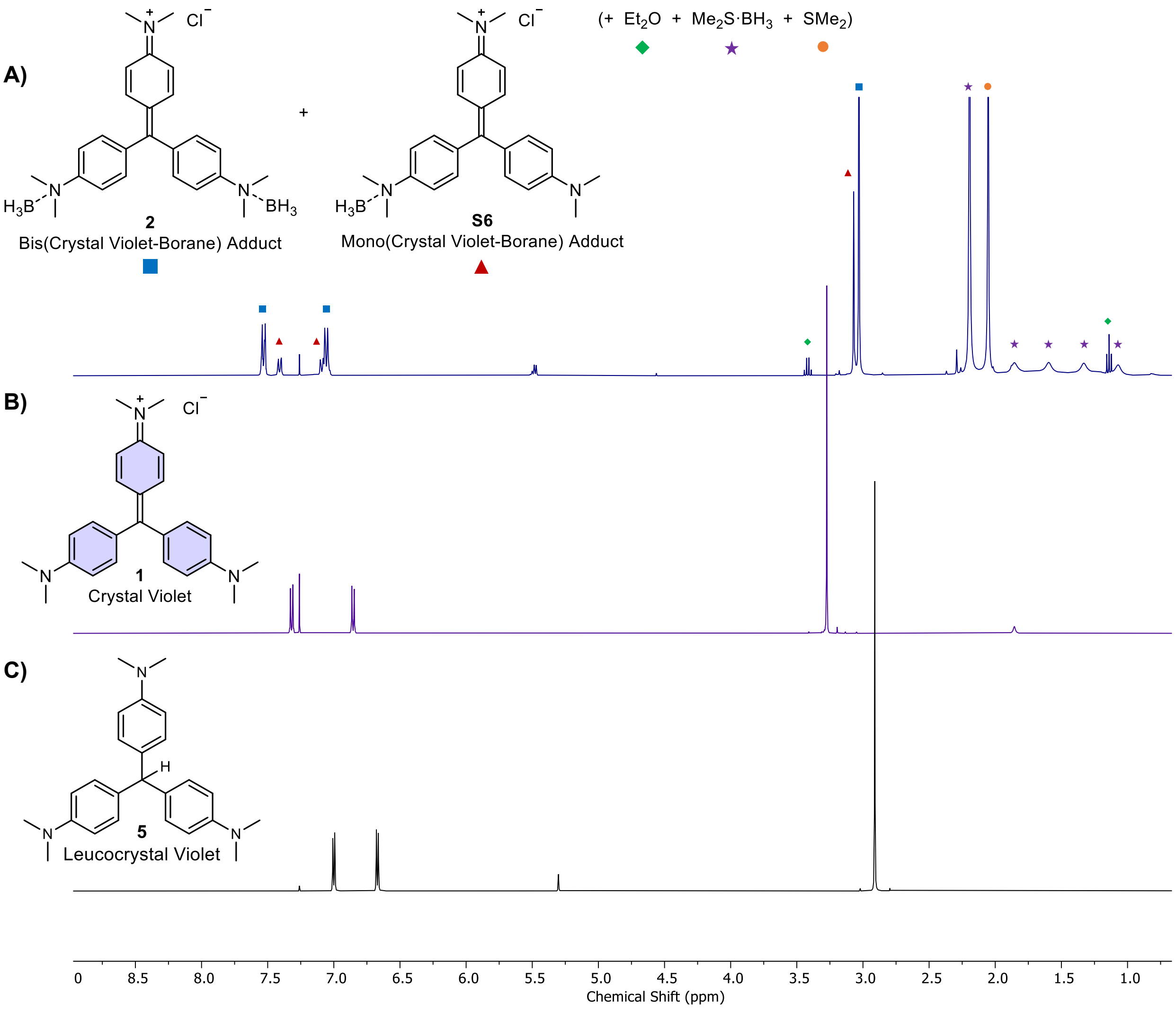

Fig. 6: ^{1}H NMR spectrum (400 MHz, $CDCl_3$): **(A)** Solution containing Crystal Violet·$(BH_3)_2$ **2**, Crystal Violet·BH_3 **S6**, $Me_2S·BH_3$, SMe_2 and Et_2O in $CDCl_3$ **(B)** Crystal Violet **1** **(C)** Leucocrystal Violet **5**

3.6.2. Characterisation of Precipitate

Under a N_2 atmosphere, crystal violet **1** (1.23 g, 3.01 mmol) was dissolved in CH_2Cl_2 (180 mL, 0.016 M) then borane dimethyl sulfide complex (1.0 mL, 10 mmol) was added. The reaction was stirred at room temperature for 1 hour. Dichloromethane and SMe_2 were removed under reduced pressure and the precipitate was dried under high vacuum for 3 hours. The bis(crystal violet-borane) adduct **2** was a blue powder (1.14 g, 2.62 mmol, 76%). ^{11}B NMR spectrum was recorded for the product in $CDCl_3$.

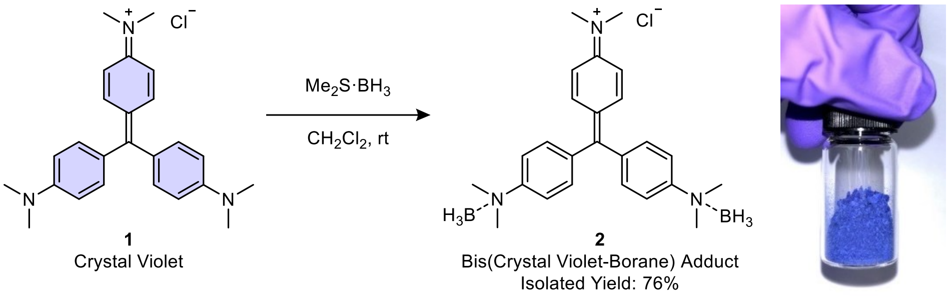

Scheme 3: Preparation and isolation of bis(crystal violet-borane) adduct **2**. The amine-borane adduct **2** was a blue powder.

^{11}B NMR: (128 MHz, $CDCl_3$) Bis-Adduct **2** -5.99 (br. s), Mono-Adduct **S6** 1.46 (br. s)

BH_3 chemical shift in accordance with data reported for *N,N*-dimethyl-aniline (mono-substituted) and tetramethyl-*p*-phenylenediamine (di-substituted) (*37*).

Due to the poor solubility of this product, it was necessary to use d_5-pyridine to record the ^{1}H and $^{13}C\{^{1}H\}$ NMR spectra. This acts as an indirect method to observe the presence of the adduct (Scheme 4).

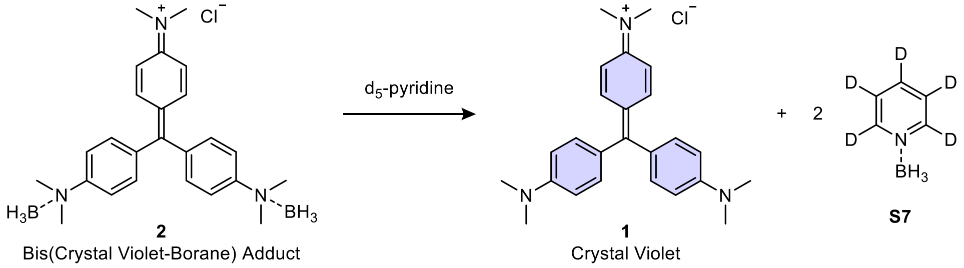

Scheme 4: Using d_5-pyridine as an indirect method to identify crystal violet-borane adduct **2** by ^{1}H and $^{13}C\{^{1}H\}$ spectroscopy.

^{1}H NMR: (600 MHz, $CDCl_3$) 7.28 (d, *J* = 8 Hz, 6H), 6.79 (d, *J* = 8 Hz, 6H),

3.37 (br. q, *J* = 96 Hz, 6H) 2.79 (s, 18H)

^{13}C{^{1}H} NMR: (126 MHz, $CDCl_3$) 134.7, 130.8, 113.5, 41.1

The IR spectrum of crystal violet **1** (Fig. 7), bis(crystal violet-borane) adduct **2** (Fig. 8) and leucocrystal violet **5** (Fig. 9) were recorded. There are distinct differences between the spectra which further illustrate the differences in these structures.

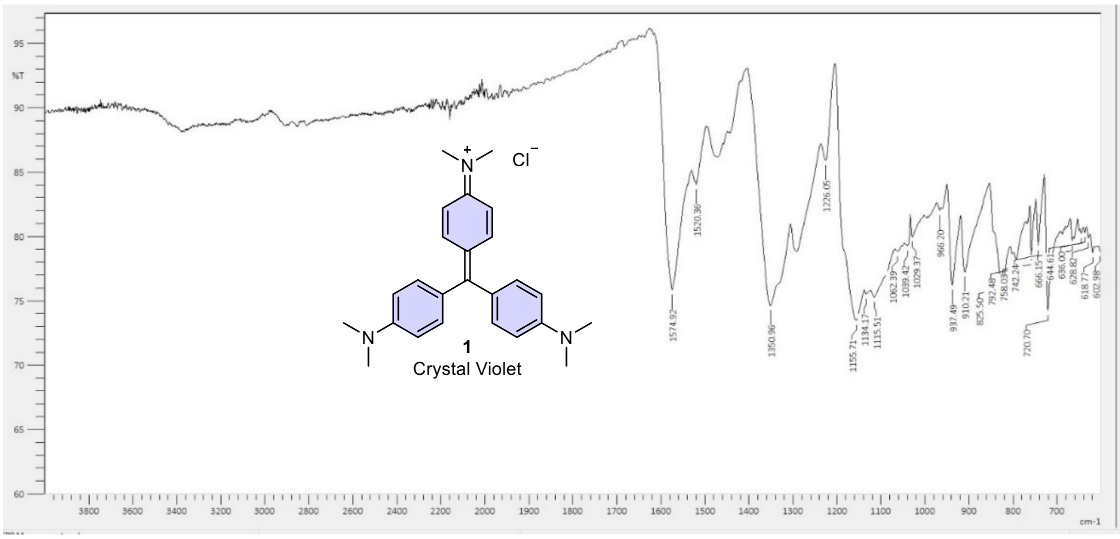

Fig. 7: IR spectrum of crystal violet **1**.

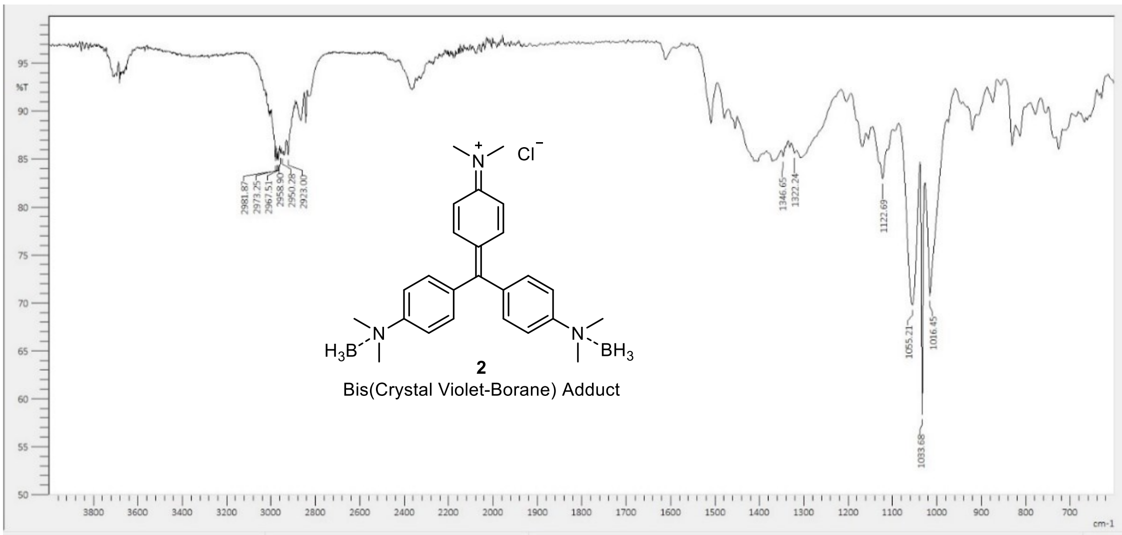

Fig. 8: IR spectrum of bis(crystal violet-borane) adduct **2**.

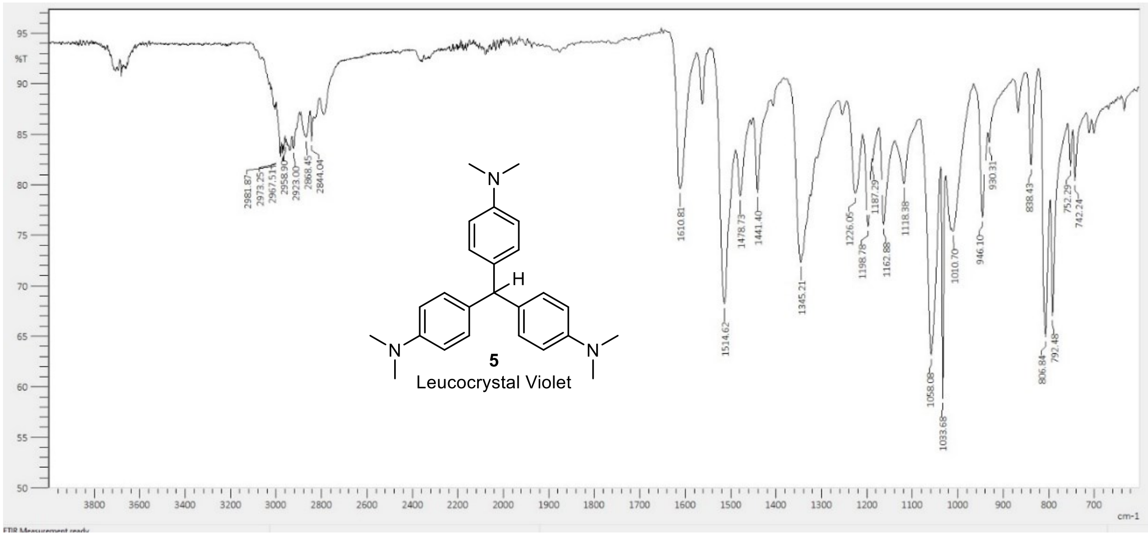

Fig. 9: IR spectrum of leucocrystal violet **5** (reduced form of crystal violet).

3.7. Detection Limit of Crystal Violet Indicator

3.7.1. Using a 0.1 mM Indicator Solution

A 1M, 0.1 M, 0.01 M, 0.001 M, 0.0001 M and 0.00001 M solution of $Me_2S{\cdot}BH_3$ in CH_2Cl_2 were prepared by serial dilution. A 0.1 mM solution of crystal violet **1** in CH_2Cl_2 was prepared and 0.5 mL was added to each vial containing 2 mL $Me_2S{\cdot}BH_3$ solution at each concentration (Fig. 10). 1 M, 0.1 M and 0.01 M all resulted in a colourless solution. A significant colour change was observed by the 0.001 M solution but it did not turn fully colourless. No colour change was observed for the 0.0001 M and 0.00001 M solutions. Therefore using 0.05 μmol of crystal violet **1** the presence of BH_3 was indicated to 0.001 M.

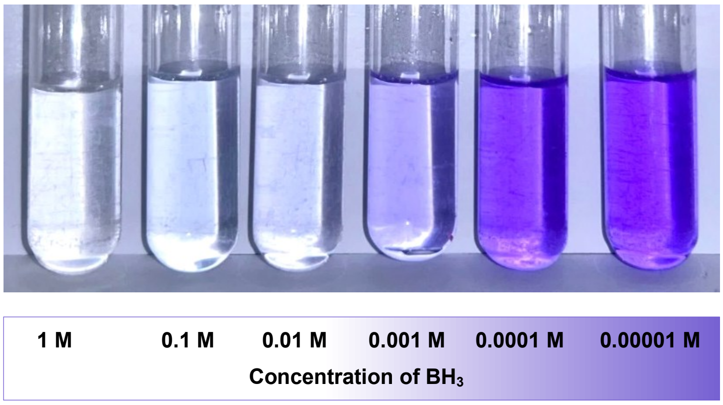

Fig. 10: Various concentrations of $Me_2S{\cdot}BH_3$ in CH_2Cl_2 after addition of the crystal violet **1** indicator (0.5 mL, 0.1 mM solution in CH_2Cl_2).

^{11}B NMR spectra were recorded for the 1 M, 0.1 M, 0.01 M, 0.001 M and 0.0001 M solutions of $Me_2S{\cdot}BH_3$ in CH_2Cl_2 (Fig. 11). The NMR analysis could only detect the presence of BH_3 up to 0.01 M; 10-fold less than the crystal violet **1** indicator.

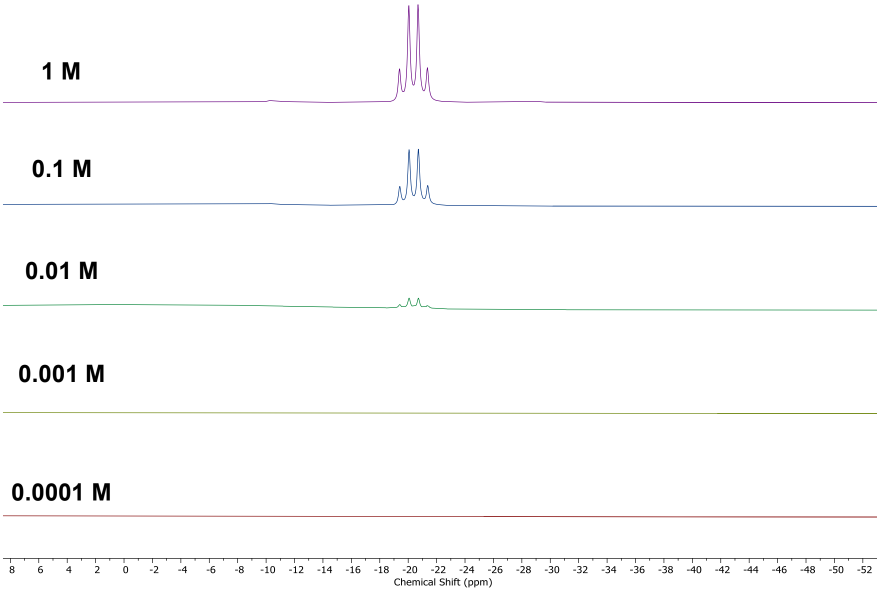

Fig. 11: ^{11}B NMR Spectrum (160 MHz) of various concentrations of BH_3 in CH_2Cl_2.

3.7.2. Using a 0.01 mM Indicator Solution

A 0.1 M, 0.01 M, 0.001 M, 0.0001 M and 0.00001 M solution of THF·BH_3 in CH_2Cl_2 were prepared by serial dilution. A 0.01 mM solution of crystal violet **1** in CH_2Cl_2 was prepared and 0.5 mL was added to each vial containing 2 mL THF·BH_3 (Fig. 12). 0.1 M, 0.01 M and 0.001 M all resulted in a colourless solution. No colour change was observed for the 0.0001 M and 0.00001 M solutions. Therefore using 0.005 µmol of crystal violet **1** the presence of BH_3 was indicated to 0.001 M.

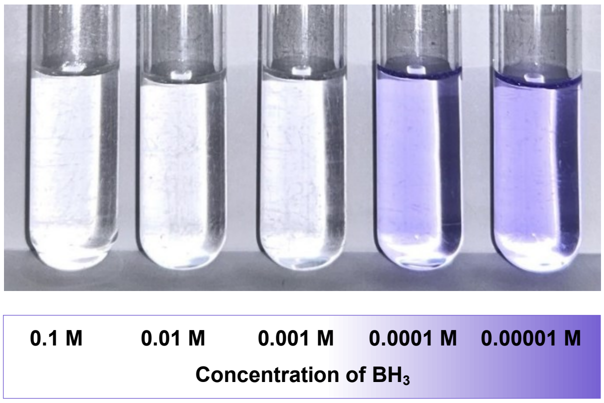

Fig. 12: Various concentrations of THF·BH_3 in CH_2Cl_2 after addition of crystal violet **1** indicator (0.5 mL, 0.01 mM solution in CH_2Cl_2).

3.7.3. Using a 0.001 mM Indicator Solution

A 0.1 M, 0.01 M, 0.001 M, 0.0001 M and 0.00001 M solution of THF·BH_3 in CH_2Cl_2 were prepared by serial dilution. A 0.001 mM solution of crystal violet **1** in CH_2Cl_2 was prepared and 1.0 mL was added to each vial containing 2 mL THF·BH_3 solution (Fig. 13). 0.1 M, 0.01 M and 0.001 M all resulted in a colourless solution. No colour change was observed for the 0.0001 M and 0.00001 M solutions. Therefore using 0.0005 µmol of crystal violet **1** the presence of BH_3 was indicated to 0.001 M. However the colour change is not obvious or clear when using an indicator solution at this concentration – see Fig. 13.Therefore the lowest concentration of indicator solution that can be used for detecting hidden borane catalysis is 0.01 mM (Fig. 12).

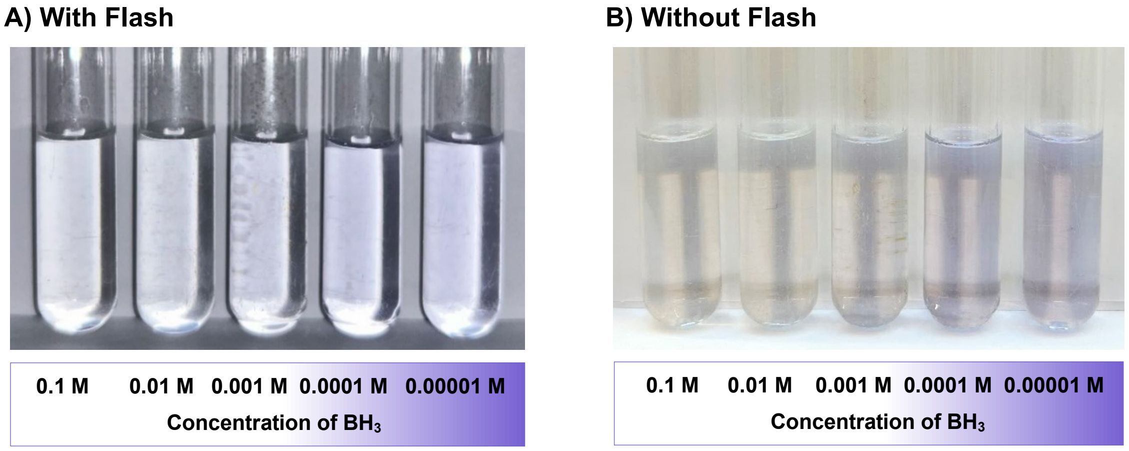

Fig. 13: Various concentrations of THF·BH_3 in CH_2Cl_2 after addition of crystal violet **1** indicator (1.0 mL, 0.001 mM solution in CH_2Cl_2).

3.8. Synthesis of 1,3,2-Dioxaborolanes

3.8.1. Preparation of Catecholborane (HBcat)

Procedure adapted from reference (*38*). A solution of catechol (12.0 g, 0.109 mol) in diethyl ether (30 mL, 3.6 M) was added dropwise over 4 hours to a solution of $Me_2S{\cdot}BH_3$ (10.0 mL, 0.105 mol) in diethyl ether (26 mL, 4.0 M) at 0 °C. The solution was warmed to room temperature and stirred until effervescence stopped. Volatiles (Et_2O and SMe_2) were removed under vacuum. The crude mixture was purified using the method detailed in *3.10. Purification Method for 1,3,2-Dioxaborolanes*.

3.8.2. Preparation of 4,4,5,5-Tetraethyl-1,3,2-dioxaborolane (HB(Epin))

Procedure adapted from reference (*39*). A solution of 3,4-diethylhexane-3,4-diol (4.80 g, 0.0275 mol) in CH_2Cl_2 (10 mL, 2.8 M) was added dropwise over 1 hour to a solution of $Me_2S{\cdot}BH_3$ (2.5 mL, 0.026 mol) in CH_2Cl_2 (10 mL, 2.6 M) at 0 °C. The solution was warmed to room temperature and stirred for 4 days. The crude mixture was purified using the method detailed in *3.10. Purification Method for 1,3,2-Dioxaborolanes.*

3.9. Testing 1,3,2-Dioxaborolanes with Crystal Violet Indicator

Clean (no BH_3) HBcat **3a,** HBpin **3b** and HB(Epin) **3c** (0.5 mmol) do not result in a colour change when added to the crystal violet **1** indicator (4 mL, 0.2 mM, 0.8 µmol) – see Fig. 14.

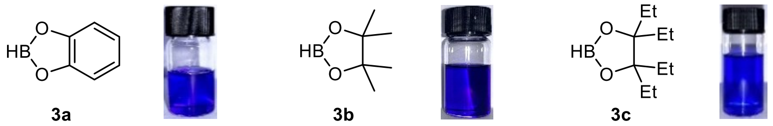

Fig. 14: Control tests to ensure no colour change was observed upon adding HBcat **3a,** HBpin **3b** or HB(Epin) **3c** to the crystal violet **1** indicator.

3.10. Purification Method for 1,3,2-Dioxaborolanes

Dialkoxyboranes are prone to undergo disproportionation, generating $B(OR)_3$ and BH_3 (*32, 33*). More specifically, for 1,3,2-dioxaborloanes (*34*), HBcat **3a,** HBpin **3b** and HB(Epin) **3c**, the disproportionation products are B_2pin_3 **S1**, $B_2(Epin)_3$ and B_2cat_3 respectively, and BH_3 (Scheme 5).

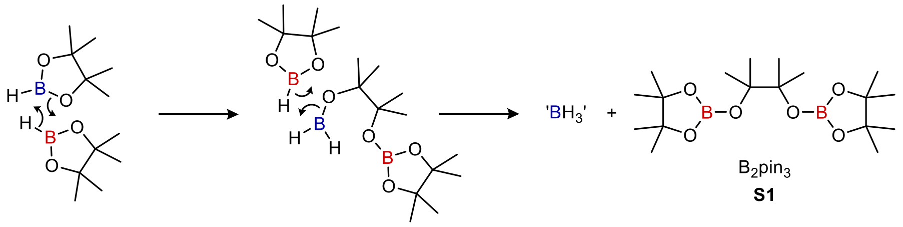

Scheme 5: Mechanism for the disproportionation of HBpin **3b** in the absence of nucleophiles (also applicable to HBcat **3a** and HB(Epin) **3c**) (*32 - 34*). 'BH_3' will exist as a dimer or as an adduct with stabilisers such as NEt_3. Nucleophilic mechanism (Scheme 1) may also be operating.

Over time, or due to improper storage of these air- and moisture-sensitive species, BH_3 forms as an impurity. As a result of the BH_3 impurity, a colour change of the indicator solution is observed (Fig. 15).

Clean HBpin

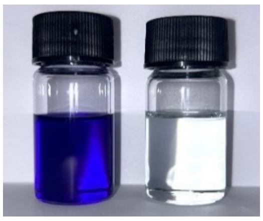

HBpin + BH_3 Impurity

Fig. 15: Crystal violet **1** indicator remains purple in the presence of clean HBpin **3b** but turns colourless in the presence of BH_3 impurity.

A purification method is detailed below (Fig. 16 and Fig. 17) for the purification of 1,3,2-dioxborloanes (e.g. HBcat **3a,** HBpin **3b** and HB(Epin) **3c**) that contain BH_3 as an impurity.

Crystal violet **1** is not soluble in 1,3,2-dioxaborolanes (e.g. HBcat **3a,** HBpin **3b** and HB(Epin) **3c**) alone, therefore a small amount (2 – 10 mL) of suitable, anhydrous solvent is required (Fig. 17, Step 1). The recommended solvents are: dichloromethane, chloroform or tetrahydrofuran. Crystal violet **1** should be added until a deep purple colour remains visible (decolourisation indicates the formation of the crystal violet-borane adduct **2**, therefore add more crystal violet **1** until the solution stays purple). The crystal violet **1** extracts any BH_3 present through precipitation. Filter cannula transfer is then required to remove the crystal violet-borane **2** precipitate (Fig. 17, Step 2). The solvent can be removed under Schlenk line vacuum or through distillation under the desired reduced pressure for the solvent (Fig. 17, Step 3). Distillation is required to purify the 1,3,2-dioxaborolanes from the residual crystal violet **1** (Fig. 17*, Step 5*). It is worth noting that prolonged heating under reduced pressure may encourage the decomposition of the 1,3,2-dioxaborolane to BH_3 – see Section S3.11. To avoid this, the Schlenk line high vacuum can be used to speed up the distillation.

The following boiling points were used:

- HBcat **3a** boiling point: 50 °C at 50 mmHg.
- HBpin **3b** boiling point: 42 °C at 50 mmHg.
- HB(Epin) **3c** boiling point: 60 °C at 50 mmHg.

Following distillation, a sample of the purified 1,3,2-dioxaborolane can be added to the crystal violet **1** indicator solution to ensure it stays purple – i.e. BH_3 impurity successfully removed. In addition, a sample can be taken and analysed by ^{11}B NMR spectroscopy.

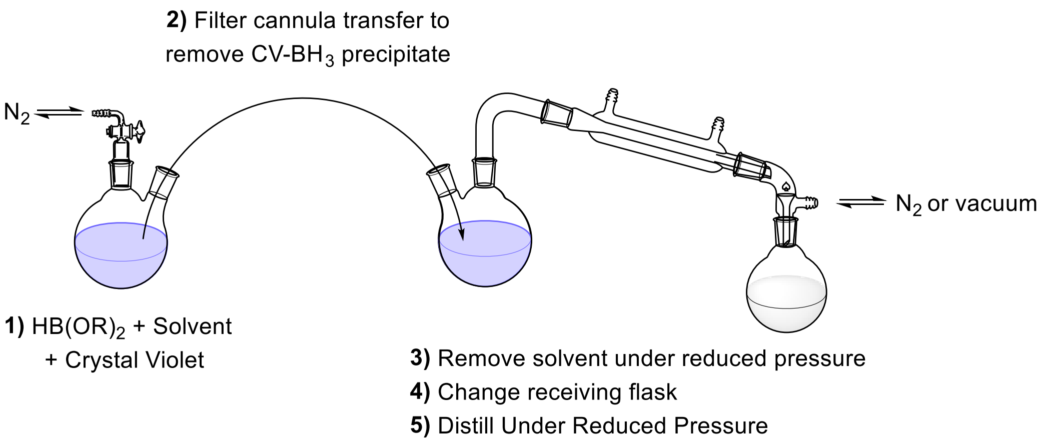

Fig. 16: Purification method for 1,3,2-dioxaborolanes, $HB(OR)_2$, to remove BH_3 impurity.

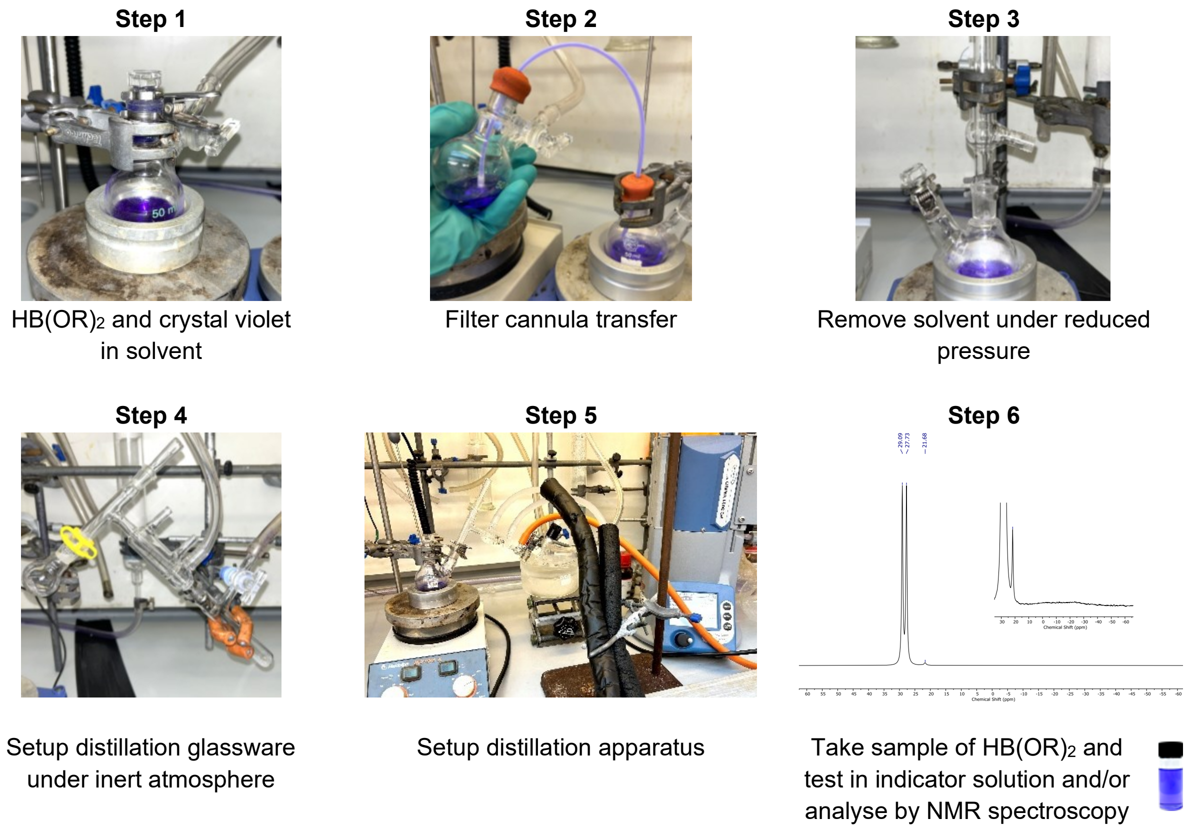

Fig. 17: Alternative illustration of the purification method for 1,3,2-dioxaborolanes, $HB(OR)_2$.

3.11. Thermal Stability of 1,3,2-Dioxaborolanes

Previously we investigated the thermal stability of 'clean' pinacolborane (no BH_3) - see Fig. 18 (*10*). The formation of BH_3 ($Et_3N{\cdot}BH_3$, q, -12.8 ppm, J = 98 Hz) was observed at 80 °C, 90 °C and 100 °C (*10*).

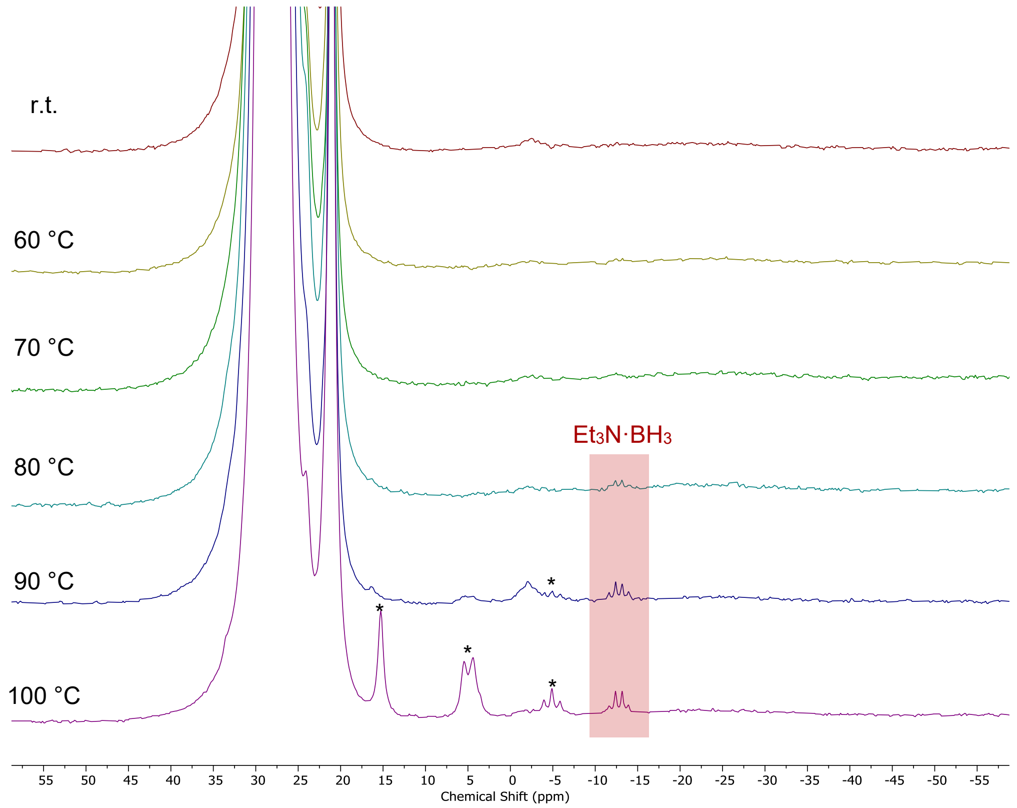

Fig. 18: ^{11}B NMR spectrum (128 MHz, neat) of the thermal stability of pinacolborane. *Decomposition products, see mechanism in Scheme 5 (10).

This work was repeated to investigate the thermal decomposition of HB(Epin) – see Fig. 19.

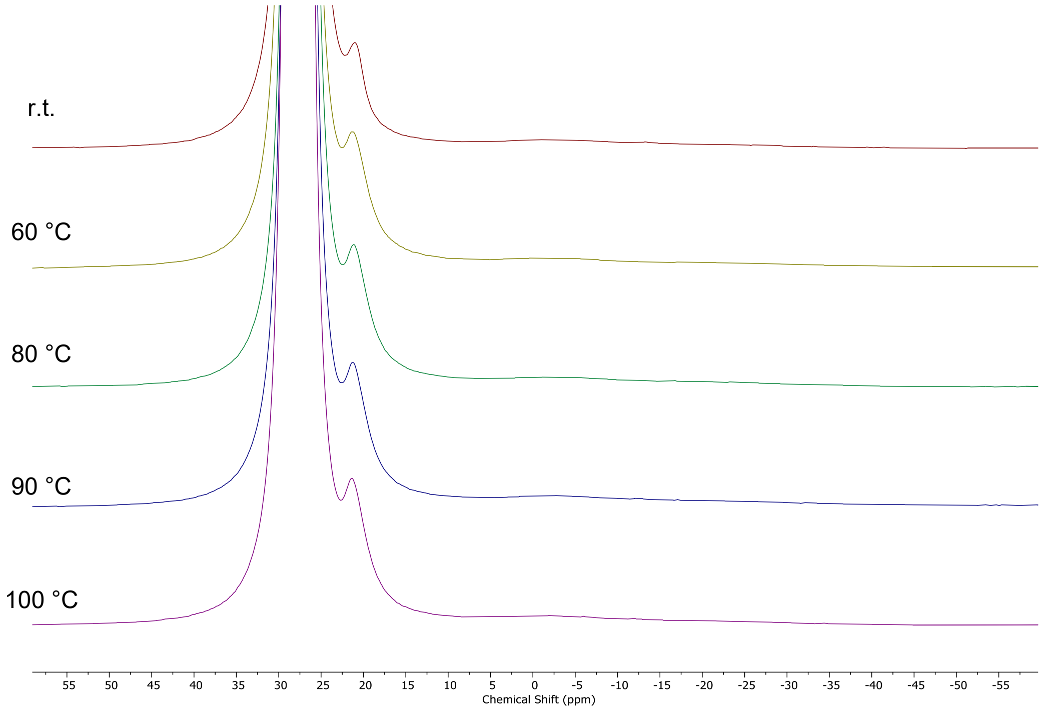

Fig. 19: ^{11}B NMR spectrum (160 MHz, toluene) of the thermal stability of HB(Epin). No decomposition products observed.

Triethylamine was distilled over calcium hydride. HB(Epin) was prepared and purified following the methods detailed in Sections S3.8.2 and S3.10, respectively. HB(Epin) (0.20 mL, 1.1 mmol), triethylamine (0.05 mL, 0.4 mmol) and anhydrous toluene (0.3 mL) were added to an NMR tube under a nitrogen atmosphere. The NMR tube was heated at the allocated temperature for 20 hours. ^{11}B NMR spectrum was recorded. No decomposition was observed after 20 hours at any temperature.

3.12. Testing Other Boron Species with Crystal Violet (Chemoselectivity)

Boron reagent (0.8 mmol, 1000 equivalents) was added to a crystal violet **1** solution (4 mL, 0.2 mM, 0.8 μmol, CH_2Cl_2). A control vial (no boron species) has been included in Fig. 20 for reference. It is worth noting that some vials look opaque due to the presence of undissolved solid boron reagent. No colour change was observed for any of the boron species tested (Fig. 21), even after 24 hours, apart from H-*B*-9-BBN and $HBCy_2$ which can disproportionate to BH_3 in solution (*33*) and therefore a slight loss of colour was observed after 15 minutes.

A solution of crystal violet **1** (1.5 x 10^{-5} M, CH_2Cl_2) was analysed by UV-Vis spectroscopy. The wavelength at maximum absorbance, λ_{max}, was 590 nm. Three crystal violet **1** solutions (1.5 x 10^{-5} M, CH_2Cl_2) had a boron reagent (0.015 M, 1000 equivalents) added – see Fig. 20. In all three solutions, the λ_{max}, was 590 nm, therefore these boron species did not change the colour of the crystal violet **1** indicator.

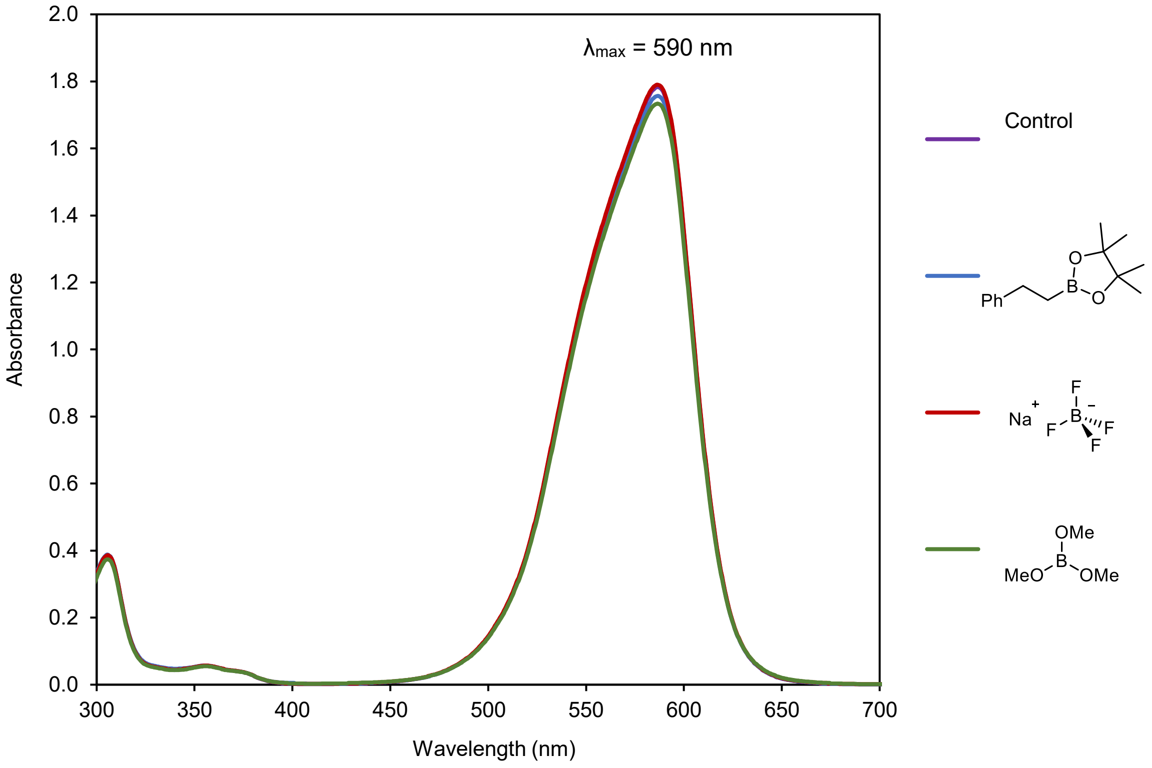

Fig. 20: UV-Vis absorption spectra of crystal violet **1** solution (1.5 x 10^{-5} M).
No change in λ_{max} in the presence of boron reagents.

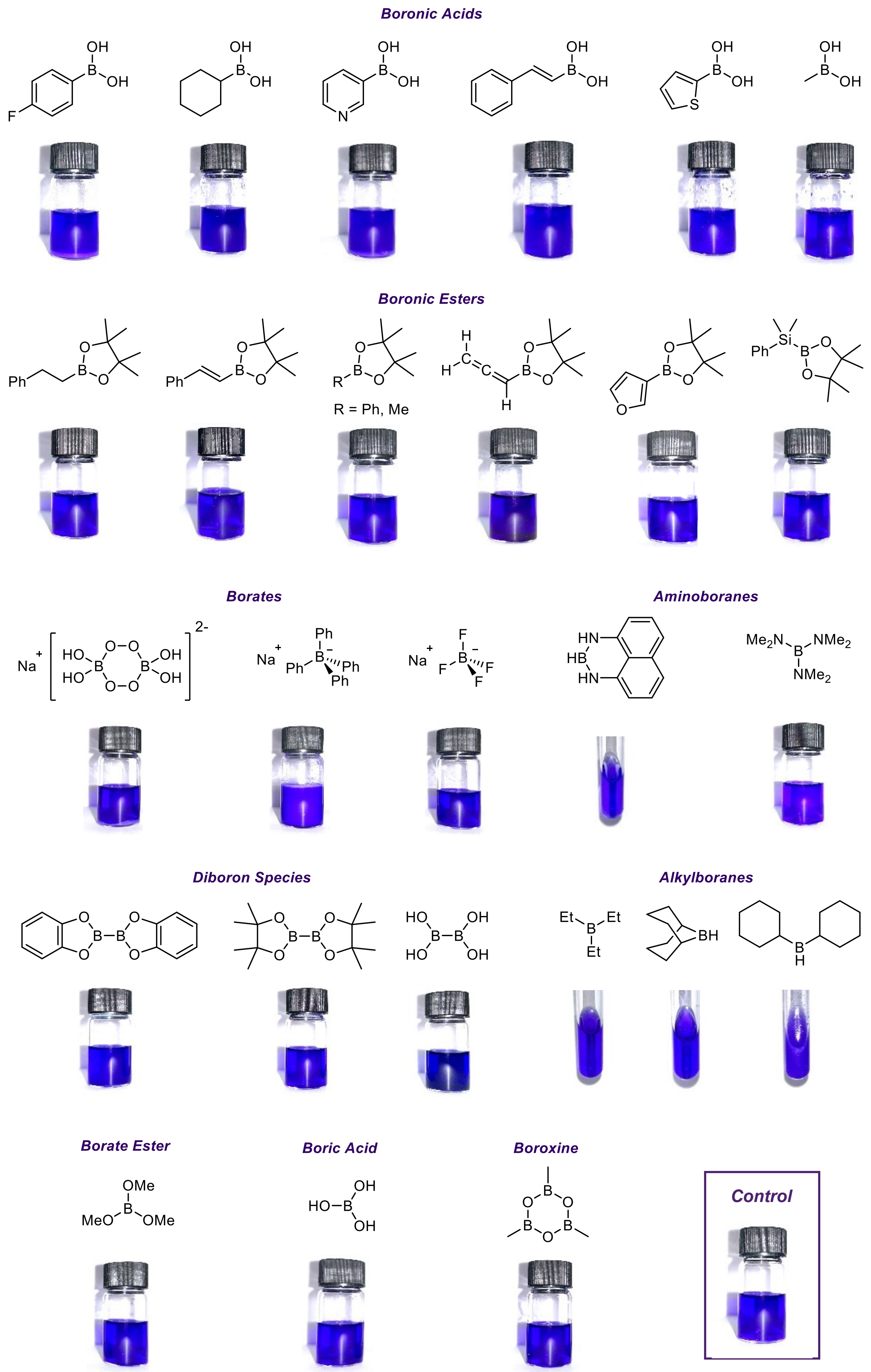

Fig. 21: Testing the crystal violet **1** indicator with various boron species to ensure no change in colour was observed.

3.13. Testing Common Hydroboration Reagents with Crystal Violet

A small sample of each reagent (0.01 – 0.1 mmol) was added to the crystal violet **1** indicator (2 mL, 0.2 mM, 0.4 μmol, CH_2Cl_2 or $CHCl_3$). No colour change was observed by the species tested in Fig. 22. Highly nucleophilic species (such as organolithium and organomagnesium) will undergo nucleophilic addition to crystal violet **1** resulting in a colourless solution. To avoid this, $CHCl_3$ should be used as the solvent since the organomagnesium/organolithium reagent will preferentially deprotonate the solvent over nucleophilic addition to the indicator (*40*). Therefore, $LiCH_2SiMe_3$, *n*BuLi, *n*Bu_2Mg and EtMgBr were tested using a crystal violet **1** solution in $CHCl_3$.

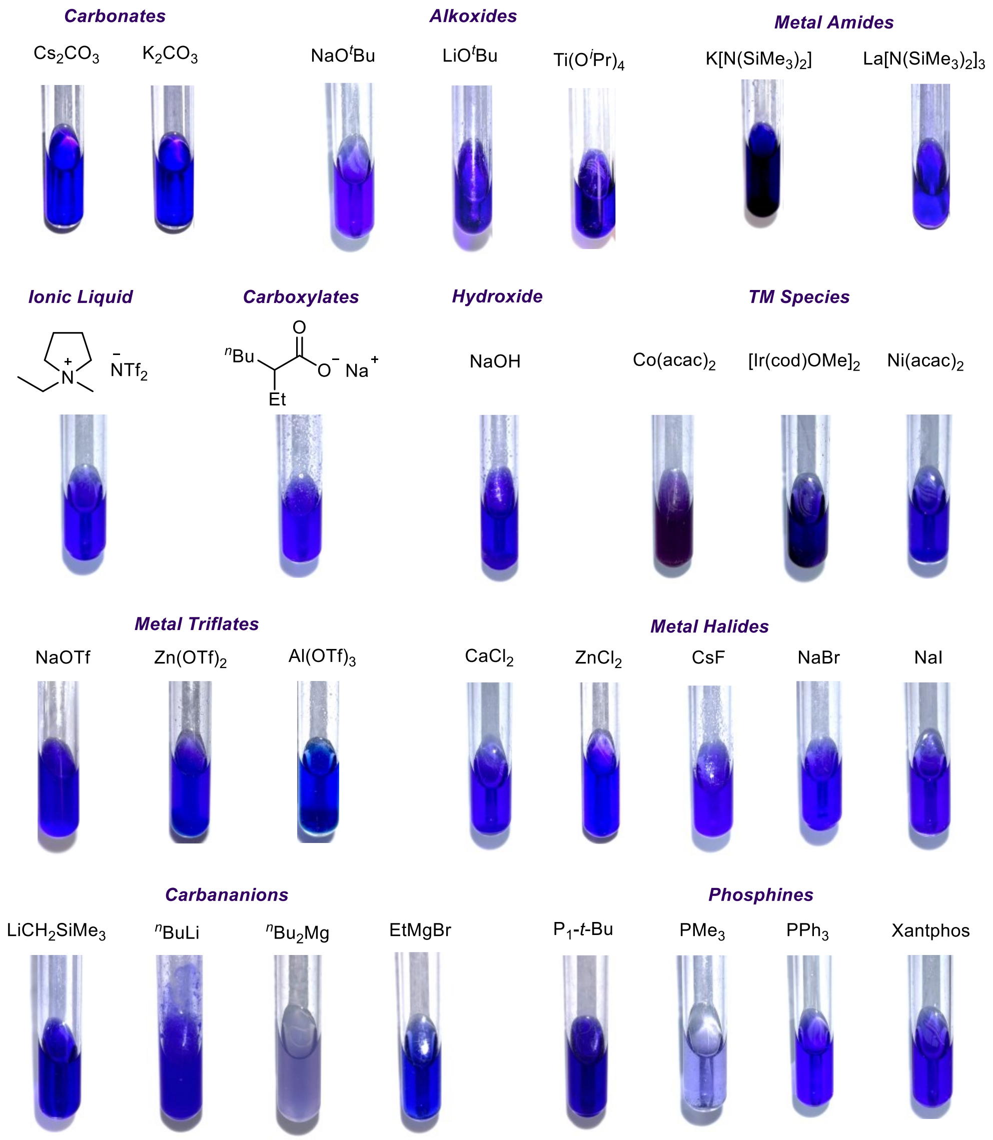

Fig. 22: Testing the crystal violet **1** indicator with common hydroboration reagents to ensure no change in colour was observed.

3.14. Adduct Competition Between Crystal Violet and Other Amines

The ability of crystal violet **1** to detect BH_3 in the presence of various amines was investigated (Table 1). Excess amine (2.5 mmol) was added to a crystal violet **1** solution in CH_2Cl_2 or THF (2 mL, 0.2 mM, 0.4 μmol). Then $Me_2S{\cdot}BH_3$ (2 mmol, 0.2 mL) or $THF{\cdot}BH_3$ (2 mmol, 0.2 mL) was added.

Table 1: Investigating that crystal violet **1** can still detect BH_3 in the presence of other amines.

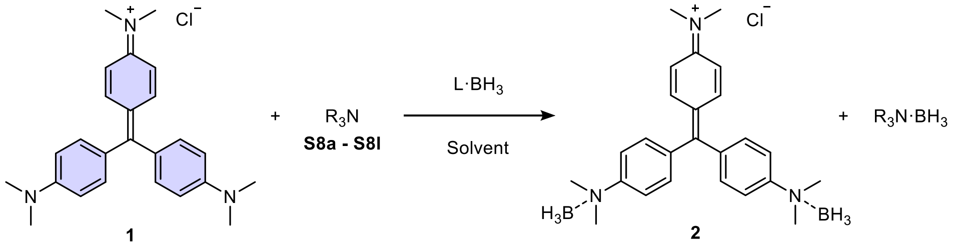

1 Crystal Violet

2 Bis(Crystal Violet-Borane) Adduct

N,N,N',N'-Tetramethylethylene diamine (TMEDA)

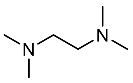

S8a

S8a + **1** | + $Me_2S{\cdot}BH_3$

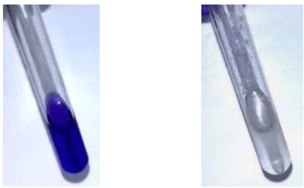

Triethylamine

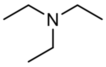

S8b

S8b + **1** | + $Me_2S{\cdot}BH_3$

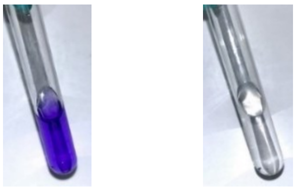

p-Toluenesulfonamide

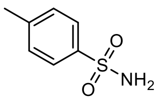

S8c

S8c + **1** | + $Me_2S{\cdot}BH_3$

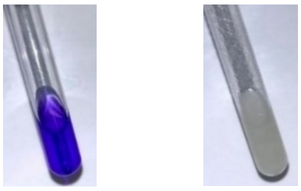

4,4'-Dimethyldiphenylamine

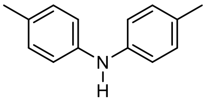

S8d

S8d + **1** | + $Me_2S{\cdot}BH_3$

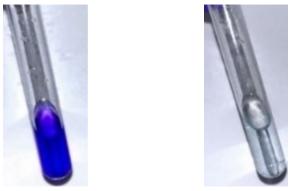

4-Methylaniline

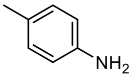

S8e

S8e + **1** | + $Me_2S{\cdot}BH_3$

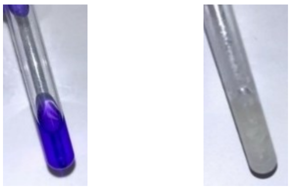

4-Dimethylaminopyridine (DMAP)

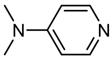

S8f

S8f + **1** | + $THF{\cdot}BH_3$

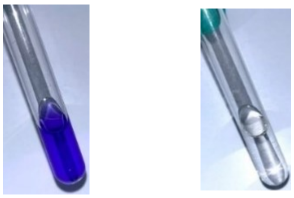

Pyridine

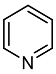

S8g

S8g + **1** | + $THF{\cdot}BH_3$

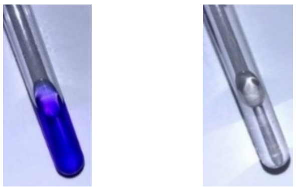

2,2'-Bipyridine

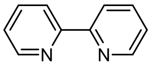

S8h

S8h + **1** | + $Me_2S{\cdot}BH_3$

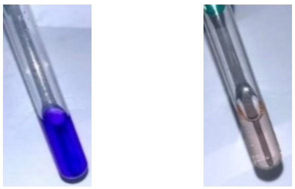

Diisopropylamine

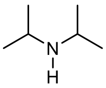

S8i

S8i + **1** | + $Me_2S{\cdot}BH_3$

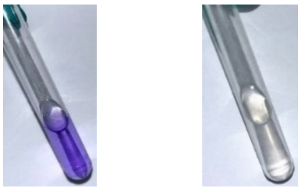

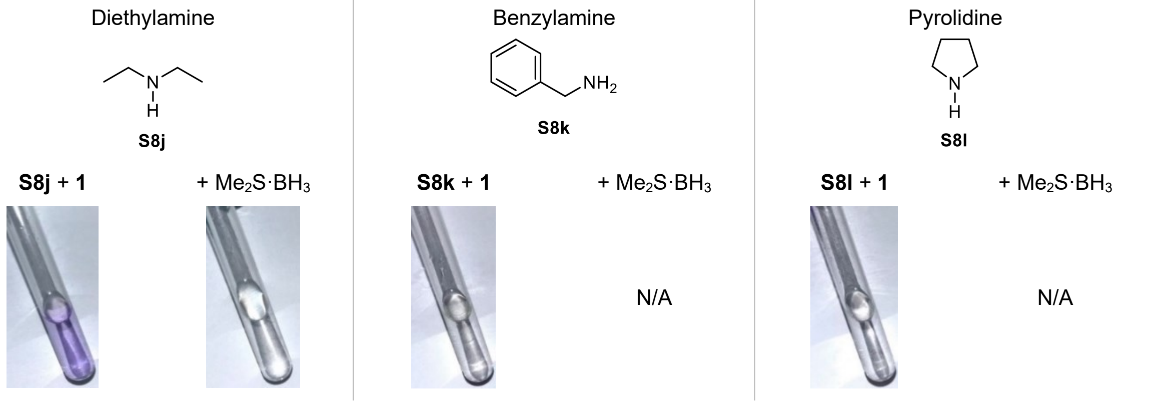

Decolourisation and effervescence were observed upon the addition of benzylamine **S8k** and pyrrolidine **S8l**. Nucleophilic primary and secondary amines will undergo nucleophilic addition to crystal violet **1** resulting in a colourless product due to the loss of conjugation (Scheme 6), as confirmed by ^{1}H NMR spectroscopy.

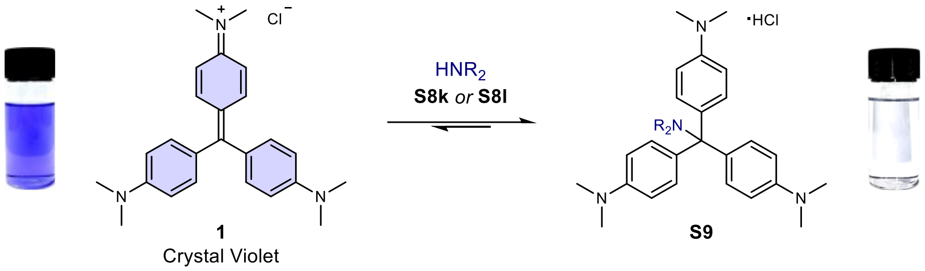

Scheme 6: Crystal violet **1** decolourises in the presence of nucleophilic primary/secondary amines.

3.15. Testing Strong Reducing Agents with Crystal Violet

For strongly reducing reagents, the hidden catalysis test must be carried out at reduced temperatures to avoid reduction of crystal violet **1**. This was exemplified with Schwartz's reagent (Cp_2ZrHCl). Crystal violet **1** solution (2 mL, 0.2 mM, 0.4 μmol) was added to Schwartz's reagent at room temperature and decolourisation was observed (Fig. 23) due to the Schwartz's reagent reducing crystal violet **1** (confirmed by ^{1}H NMR, Fig. 24). Crystal violet **1** solution (2 mL, 0.2 mM, 0.4 μmol) was added to Schwartz's reagent at 0 °C and no colour change was observed initially. After 5 minutes decolourisation becomes noticeable. It is worth noting that hidden catalysis (BH_3) can still be detected at 0 °C by an instant colour change from purple to colourless.

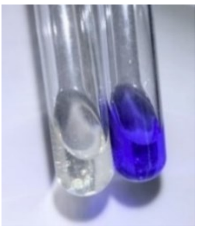

Fig. 23: **LEFT** Crystal violet **1** solution added to Schwartz's reagent (Cp_2ZrHCl) in dichloromethane at room temperature. **RIGHT** Crystal violet **1** solution added at 0 °C.

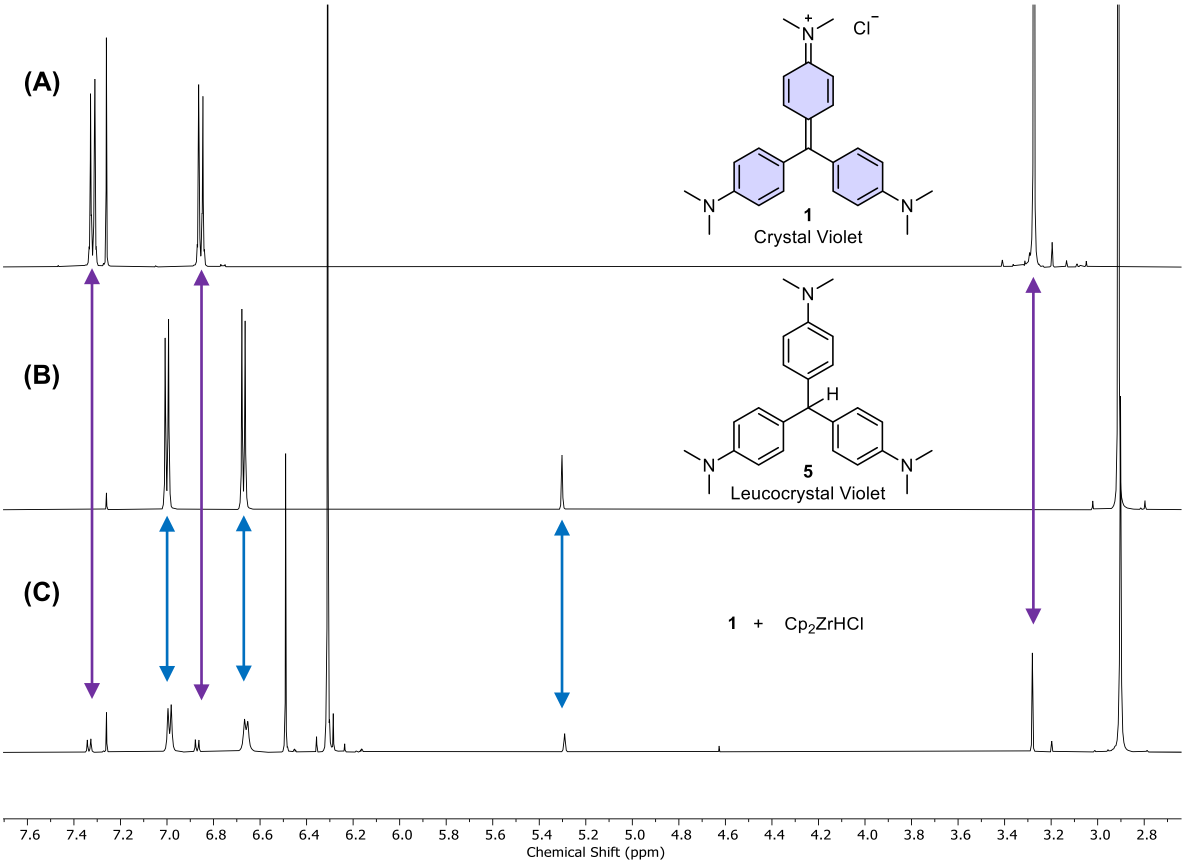

Fig. 24: (A) ^{1}H NMR of crystal violet **1** in $CDCl_3$. **(B)** ^{1}H NMR of reduced crystal violet **5** in $CDCl_3$. **(C)** Reaction between crystal violet **1** and Schwartz's reagent (Cp_2ZrHCl) in $CDCl_3$. Formation of reduced crystal violet **1** observed.

3.16. Crystal Violet and Brønsted Acids

3.16.1. Crystal Violet as a Brønsted Acid Indicator

Crystal violet **1** is also a pH indicator. It changes colour from purple to yellow in the presence of acid (Fig. 25). A solution of crystal violet **1** (10 mL, 1.5 x 10^{-5} M, H_2O) was prepared through serial dilutions. UV-Vis absorbance spectrum was recorded (Fig. 25, Purple). Hydrochloric acid (0.1 mL, 1 M) was added and a green solution was observed. UV-Vis absorbance spectrum was recorded (Fig. 25, Green). Hydrochloric acid (1.0 mL, 1 M) was added and a yellow solution was observed. UV-Vis absorbance spectrum was recorded (Fig. 25, Yellow).

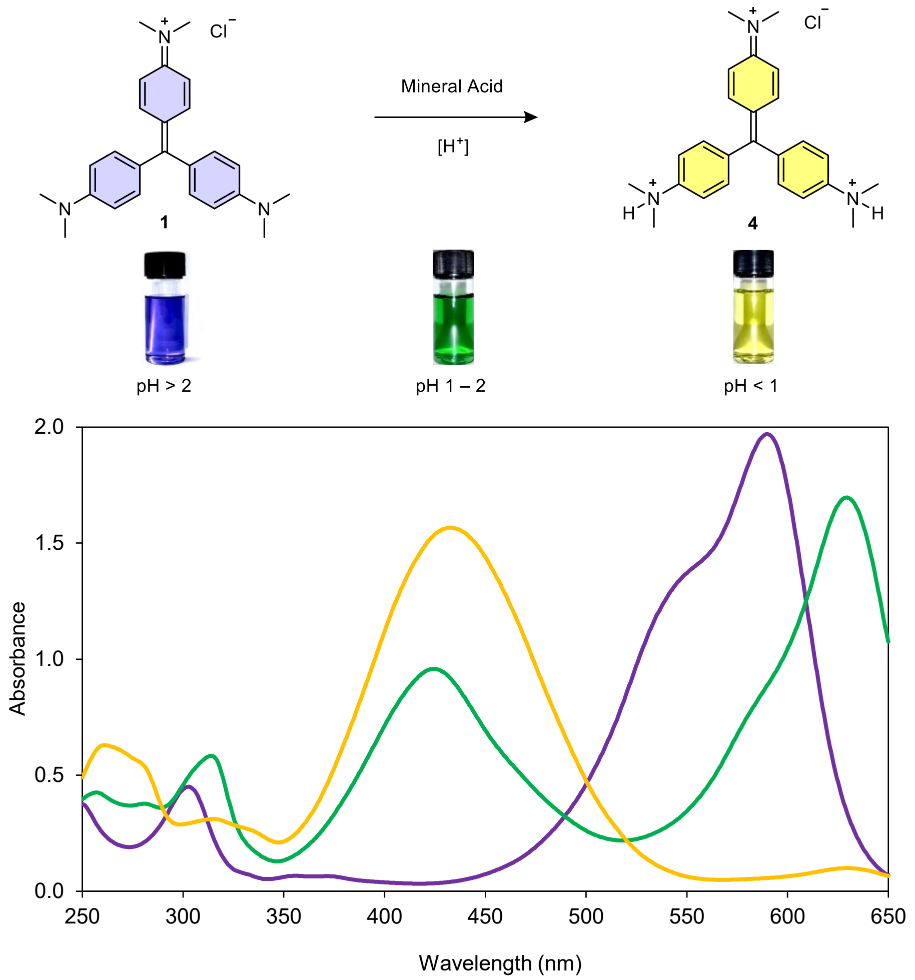

Fig. 25: Crystal violet **1** as a pH indicator and the UV-vis spectrum of each coloured solution.

Since crystal violet **1** can detect acidic conditions, it would be very useful if it could detect hidden Brønsted acid catalysis when Lewis acid catalysis is expected. However, this indicator is not sensitive enough to detect the low/trace concentration of Brønsted acid expected under reaction conditions.

A 1M, 0.1 M, 0.01 M, 0.001 M, 0.0001 M and 0.00001 M solution of HCl in H_2O were prepared by serial dilution. A 0.1 mM solution of crystal violet **1** in H_2O was prepared and 0.5 mL was added to each vial containing 2 mL of the HCl solution (see Fig. 26). A significant colour change was observed by the 1 M and 0.1 M solutions which turned yellow and green respectively. A less obvious colour change was observed by the 0.01 M HCl solution which resulted in a dark blue solution. A very slight colour change was observed by the 0.001M HCl solution which turned a slightly darker purple. No colour change was observed by the 0.0001 M HCl. Therefore, using 0.05 µmol of crystal violet **1** can detect H^+ up to 0.01 M for strong acids in aqueous solution (see Fig. 26). It should be noted that organic solvents and/or weaker acids may result in lower dissociation compared to acids in aqueous solvent, and that reactions affected by hidden Brønsted acid catalysis may generate very low concentrations of (complex) acids.

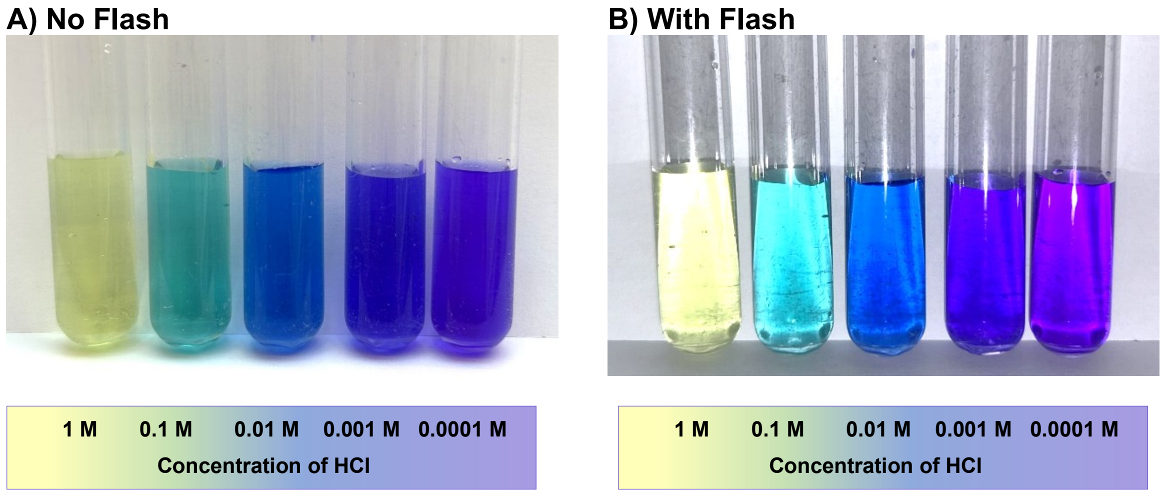

Fig. 26: Various concentrations of aqueous HCl after addition of crystal violet **1** indicator (0.5 mL, 0.1 mM solution in H_2O).

3.16.2. Crystal Violet Indicator with Main Group Halides

A small sample of each reagent (~0.01 mmol) was added to the crystal violet **1** solution (2 mL, 0.2 mM, 0.4 μmol, CH_2Cl_2). Yellow solutions were observed due to the presence of acid from main-group halide hydrolysis by trace water (Fig. 27).

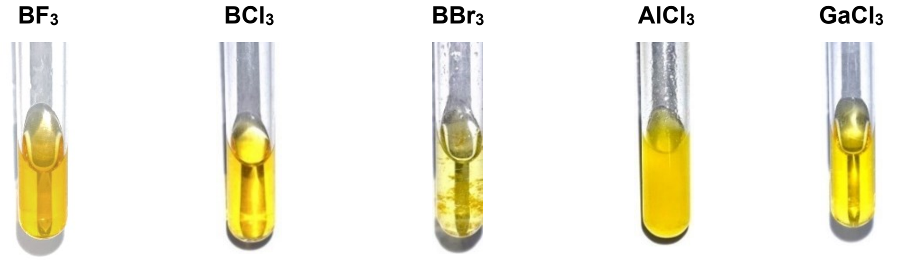

Fig. 27: Main group halides added to crystal violet **1** solution.

3.17. Detecting 'Catalyst'-mediated Dioxaborolane (HBpin) Decomposition

Various species used as 'catalysts' for hydroboration were tested for 'catalyst'-mediated decomposition of HBpin **3b** to BH_3 (Scheme 7) – mechanism is shown in Scheme 1. These tests were carried out at room temperature to avoid any thermal decomposition of HBpin **3b** since recent studies revealed that high temperatures (>80 °C) encourage the disproportionation of HBpin **3b** to BH_3 (Mechanism shown in Scheme 5). Therefore, any BH_3 present is due to 'catalyst'-mediated decomposition of HBpin **3b**.

Pinacolborane **3b** (3 - 5 mmol) and 'catalyst' (0.01 – 0.1 mmol) were added to solvent (0.2 – 1 mL) under an argon or nitrogen atmosphere.

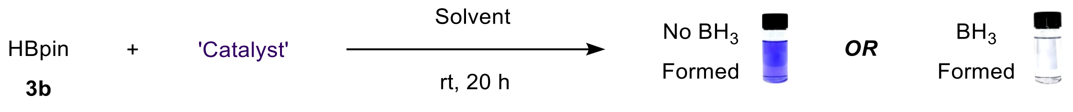

Scheme 7: Screening various common hydroboration 'catalysts' for 'catalyst'-mediated decomposition of HBpin **3b** to BH_3.

Crystal violet **1** solution (0.5 mL, ~0.2 mM) was added and a colour change from violet to colourless was observed when BH_3 had formed from decomposition of HBpin **3b** (see Table 2). Alternatively, a sample of the reaction can be added to a vial containing the indicator solution. Both methods are equally successful for detecting BH_3. For reactions with coloured solutions, it is best to take a sample of the reaction and add it to the indicator. For organolithium and organomagnesium, the sampling method A must be used and the indicator solution must be prepared using $CHCl_3$. All reactions were analysed by ^{11}B NMR spectroscopy to confirm the results of the indicator (see Fig. 41 to 64 for ^{11}B NMR spectra).

Table 2: Using the crystal violet **1** colourimetric procedure to detect hidden BH_3 generated from 'catalyst'-mediated decomposition of HBpin **3b**.

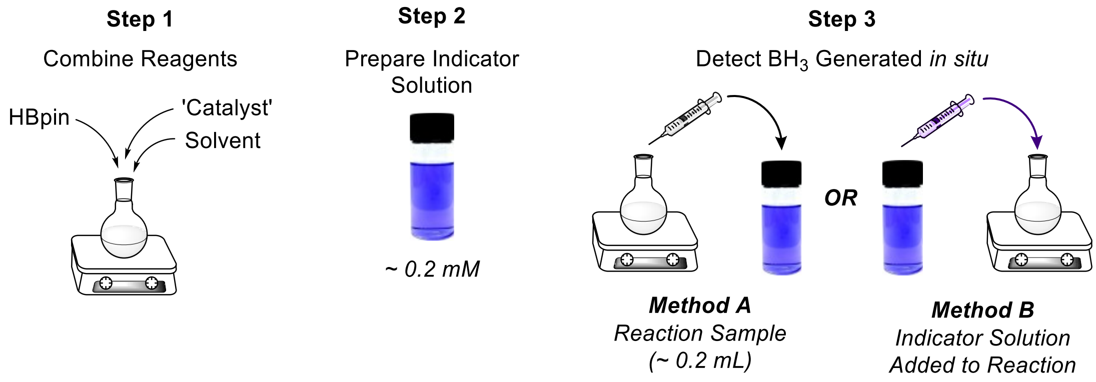

Entry	'Catalyst'	Reaction Solvent	Method	Indicator Solution		^{11}B NMR
1	$CaCl_2$	Neat	A	Purple	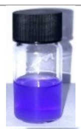	No BH_3
2	$Co(acac)_2$	THF	A	Purple	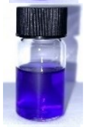	No BH_3
3	$Co(acac)_2$ + Xantphos	THF	A	Purple	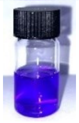	No BH_3
4	$Ni(acac)_2$	THF	A	Purple	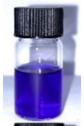	No BH_3
5	$Ir(cod)OMe]_2$	CH_2Cl_2	A	Purple	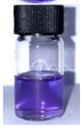	No BH_3
6	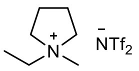	Neat	B	Purple	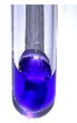	No BH_3
7	$Zn(OTf)_2$	Toluene	A	Purple	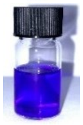	No BH_3

8	NaOTf	Toluene	B	Purple	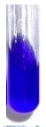	No BH_3
9	Cp_2ZrHCl	CH_2Cl_2	B	Purple	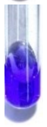	No BH_3
10	LiO^{t}Bu	Toluene	B	Colourless	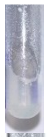	BH_3 observed
11	NaO^{t}Bu	Toluene	B	Colourless	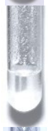	BH_3 observed
12	NaOH	Toluene	B	Colourless	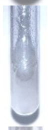	BH_3 observed
13	$K[N(SiMe_3)_2]$	Toluene	B	Colourless	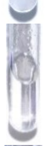	BH_3 observed
14	$La[N(SiMe_3)_2]_3$	Toluene	B	Colourless	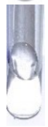	BH_3 observed
15	Cs_2CO_3	Toluene	B	Colourless	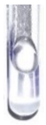	BH_3 observed
16	K_2CO_3	Toluene	B	Colourless	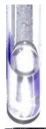	BH_3 observed
17	$LiCH_2SiMe_3$	Toluene	A	Colourless	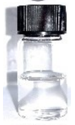	BH_3 observed
18	^{n}BuLi	Toluene	A	Colourless	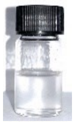	BH_3 observed
19	$^nBu_2Mg$	Toluene	A	Colourless	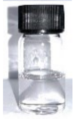	BH_3 observed
20	EtMgBr	Toluene	A	Colourless	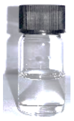	BH_3 observed
21	PMe_3	Toluene	B	Colourless	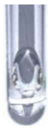	BH_3 observed

22	P_1-*t*-Bu	Toluene	B	Colourless	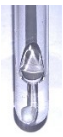	BH_3 observed
23	CsF	THF	B	Colourless	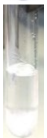	BH_3 observed
24	BEt_3	Toluene	B	Colourless	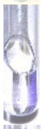	BH_3 observed
25	$NaBH_4$	Toluene	B	Colourless	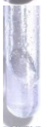	BH_3 observed
26	$NaHBEt_3$	Toluene	B	Colourless	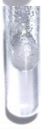	BH_3 observed
27	$Ti(O^iPr)_4$	Toluene	A	Colourless	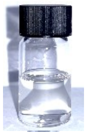	BH_3 observed
28	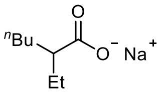	CH_2Cl_2	B	Colourless	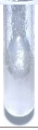	BH_3 observed

3.18. Hydroboration in the Presence of Crystal Violet

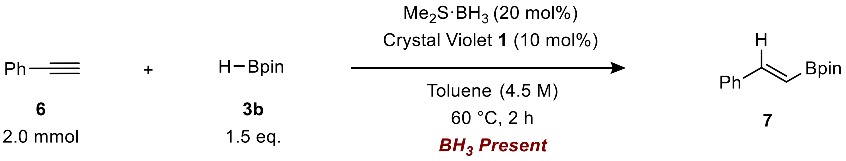

Scheme 8: BH_3-catalysed hydroboration of phenylacetylene **6** with HBpin **3b** in the presence of crystal violet **1**.

An internal standard solution of 1,3,5-trimethoxybenzene (1.0 M in toluene) was prepared. Crystal violet **1** (90 mg, 0.22 mmol) was added to a reaction vial under an argon atmosphere. Pinacolborane **3b** (0.44 mL, 3.0 mmol), internal standard solution (0.2 mL, 0.2 mmol), toluene (0.2 mL) and borane-dimethyl sulfide (38 μL, 0.4 mmol, 20 mol% BH_3) were added. Phenylacetylene **6** (0.22 mL, 2.0 mmol) was added and the reaction was heated to 60 °C. Aliquots were taken from the reaction at given time intervals and quenched in Et_2O. The yield of the alkenyl boronic ester **7** was determined by ^1H NMR spectroscopy in $CDCl_3$ by comparison of diagnostic peak [6.18 (d, *J* = 18 Hz, 1H)] with the internal standard [6.09 (s, 3H)], using an average of two runs. See Fig. 28 for exemplar ^1H NMR spectrum with diagnostic peaks of key species labelled. A colourless solution was observed, indicating the formation of the crystal violet-borane adduct **2**. However, the

reaction profile indicates that the reaction is not inhibited (Fig. 29). Therefore, the crystal violet-borane adduct **2** is labile and does not inhibit BH_3-catalysed hydroboration reactions.

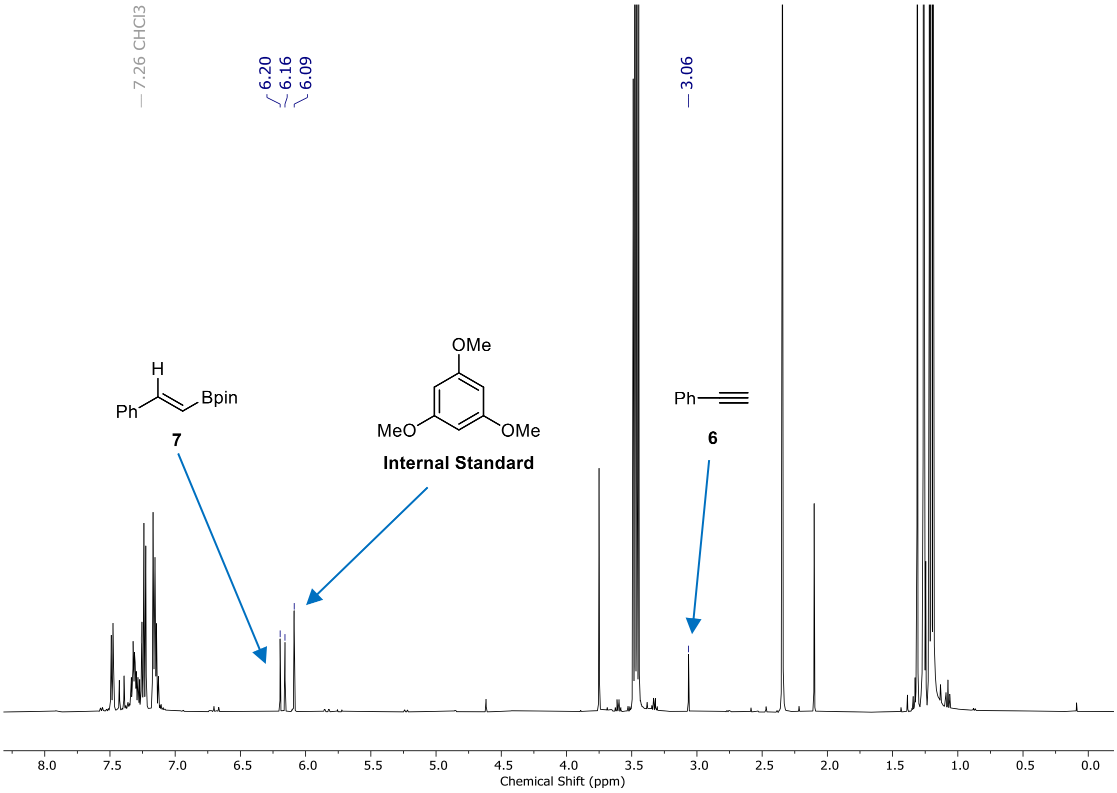

Fig. 28: ^1H NMR spectrum (600 MHz, $CDCl_3$) spectrum with diagnostic peaks labelled for key species.

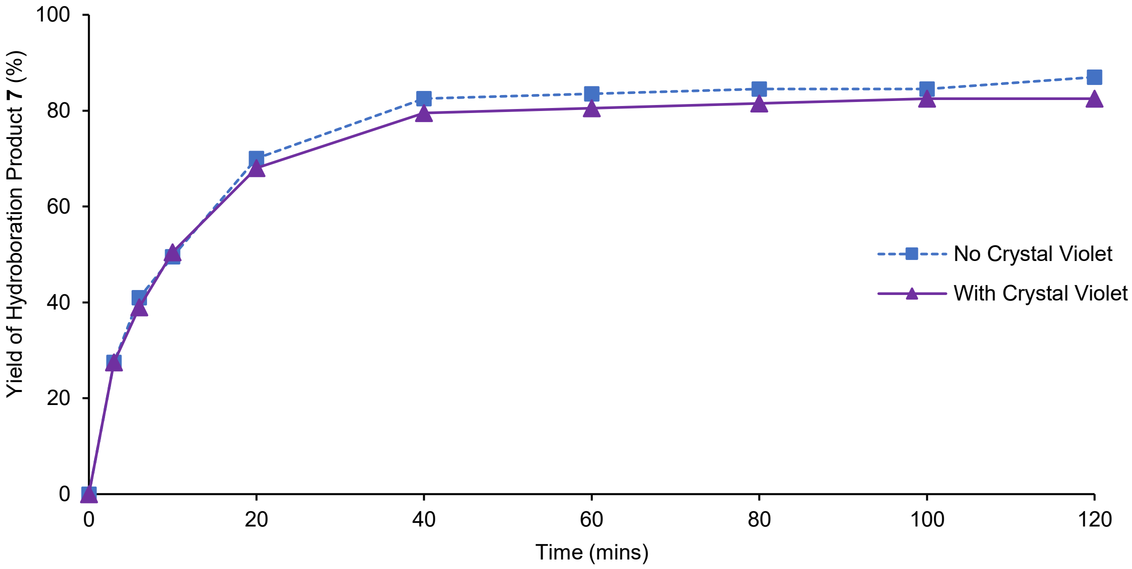

Fig. 29: Reaction monitoring for the BH_3-catalysed hydroboration of phenylacetylene **6** with HBpin **3b** in the presence of crystal violet **1**.

3.19. Hydroboration Catalysed by Crystal Violet-Borane Complex

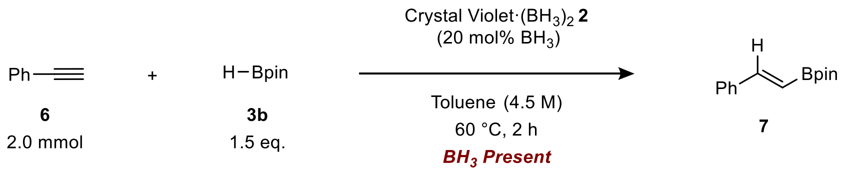

Scheme 9: (Crystal violet-borane adduct **2**)-catalysed hydroboration of phenylacetylene **6** with HBpin **3b**.

An alternative method to determine whether crystal violet **1** would inhibit BH_3-catalysed hydroboration reactions was to use the crystal violet-borane adduct **2** as a source of BH_3 for the hydroboration of phenylacetylene **6** with HBpin **3b**.

An internal standard solution of 1,3,5-trimethoxybenzene (1.0 M in toluene) was prepared. Crystal violet-borane complex **2** (90 mg, 0.22 mmol) was added to a reaction vial under Ar. Pinacolborane **3b** (0.44 mL, 3.0 mmol), internal standard solution (0.2 mL, 0.2 mmol) and toluene (0.2 mL) were added. Phenylacetylene **6** (0.22 mL, 2.0 mmol) was added and the reaction was heated to 60 °C. Aliquots were taken from the reaction at given time intervals and quenched in Et_2O. The yield of the alkenyl boronic ester **7** was determined by ^{1}H NMR spectroscopy in $CDCl_3$ by comparison of diagnostic peak [6.18 (d, *J* = 18 Hz, 1H)] with the internal standard [6.09 (s, 3H)], using an average of two runs. See Fig. 28 for exemplar ^{1}H NMR spectrum with diagnostic peaks of key species labelled.

Product formation was observed (see Fig. 30), therefore the crystal violet-borane adduct **2** was sufficiently labile to allow for the BH_3-catalysed hydroboration of phenylacetylene **6** with HBpin **3b**. It is worth noting that the crystal violet-borane complex **2** was not very soluble, therefore, a much slower reaction was observed (Fig. 30) compared to that of the $Me_2S{\cdot}BH_3$ catalysed hydroboration in section *3.18. Hydroboration in the Presence of Crystal Violet* (Fig. 29).

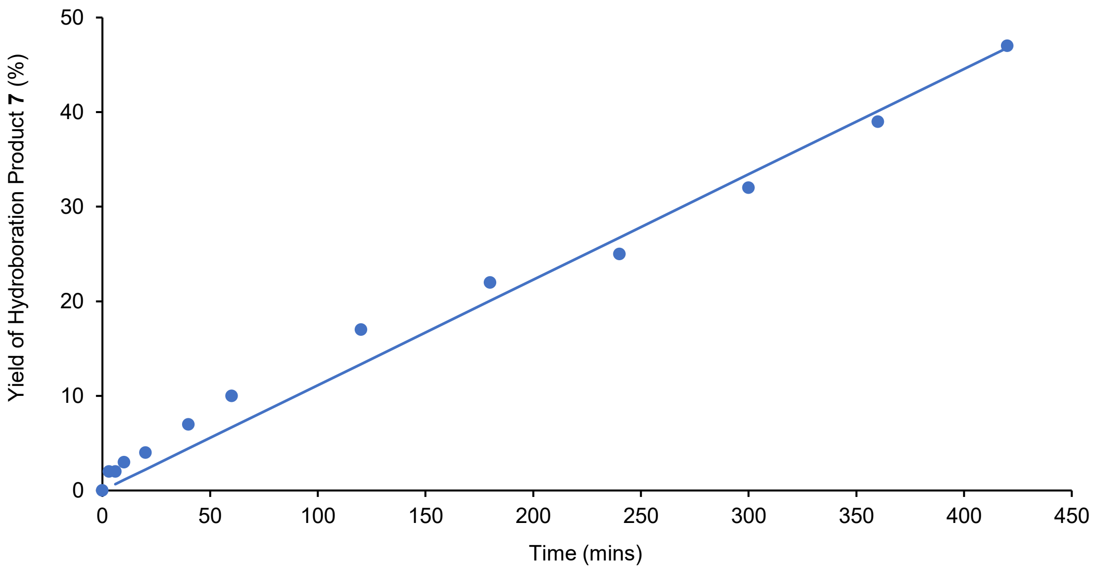

Fig. 30: Reaction monitoring for the (Crystal violet-borane adduct **2**)-catalysed hydroboration of phenylacetylene **6** with HBpin **3b**.

3.20. Hidden BH_3 Detection – Full Reaction Conditions

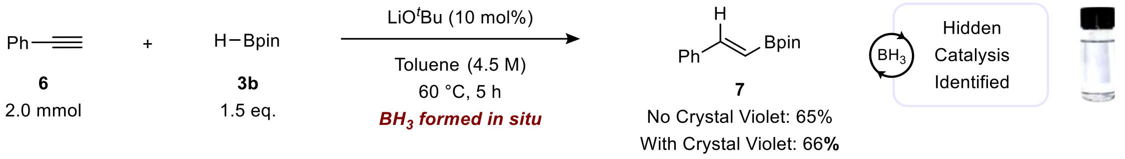

Scheme 10: Hidden BH_3-catalysed hydroboration of phenylacetylene **6** with HBpin **3b** (LiO^{t}Bu-mediated decomposition of HBpin **3b** to BH_3).

An internal standard solution of 1,3,5-trimethoxybenzene (1.0 M in toluene) was prepared. Pinacolborane **3b** (0.52 mL, 3.6 mmol), LiO^{t}Bu (16 mg, 0.2 mmol) and internal standard solution (0.2 mL, 0.2 mmol) were added to toluene (0.24 mL) under an argon atmosphere. Phenylacetylene **6** (0.22 mL, 2.0 mmol) was added. The reaction was stirred at 60 °C for 5 hours. The yield of the alkenyl boronic ester **4** was determined by ^{1}H NMR spectroscopy in $CDCl_3$ by comparison of diagnostic peak [6.18 (d, *J* = 18 Hz, 1H)] with the internal standard [6.09 (s, 3H)]. See Fig. 28 for exemplar ^{1}H NMR spectrum with diagnostic peaks of key species labelled. NMR yield of 65% was observed.

This reaction was repeated in the presence of crystal violet **1**. Crystal violet **1** solution in toluene (0.24 mL, 0.2 mM, 0.048 μmol) was added instead of toluene and a colour change from violet to colourless was observed. NMR yield of 66% was observed.

3.21. True Catalysis Example – Schwartz's Reagent

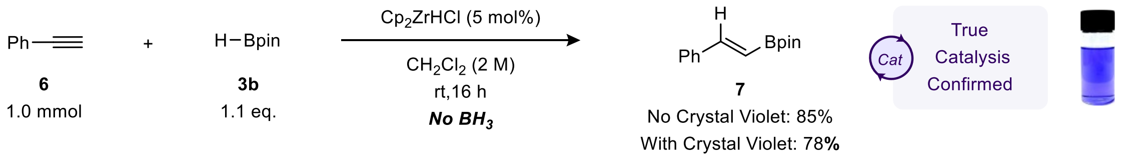

Scheme 11: Example of a 'true' catalysed hydroboration of phenylacetylene **6** with HBpin **3b** using Schwartz's reagent (hydrozirconation).

Phenylacetylene **6** (0.11 mL, 1.0 mmol) and pinacolborane **3b** (0.16 mL, 1.1 mmol) were added to dichloromethane (0.5 mL, 2 M). This solution was added to Schwartz's reagent (13 mg, 5.0 mol%) at 0 °C. The reaction was stirred at room temperature for 16 hours. The yield of the alkenyl boronic ester **7** was determined by ^{1}H NMR spectroscopy in $CDCl_3$ by comparison of diagnostic peak [6.18 (d, *J* = 18 Hz, 1H)] with the internal standard [6.09 (s, 3H)]. See Fig. 28 for exemplar ^{1}H NMR spectrum with diagnostic peaks of key species labelled. NMR yield of 85% was observed. Data were in accordance with those previously reported.

This reaction was repeated but with crystal violet **1** present. Crystal violet **1** solution (1 mL, 0.001 mmol) was added and no decolourisation was observed initially but after 5 minutes decolourisation was noticeable due to the Schwartz's reagent reducing crystal violet **1**. A 78% yield of **7** was observed by ^{1}H NMR spectroscopy. Data in accordance with literature (*25*).

3.22. Comparison to TMEDA Inhibition Method

One of the current hidden borane catalysis detection methods involved inhibition of hidden BH_3-catalysed pathways by TMEDA (6). However this method was only shown to work up to 60 °C. The TMEDA inhibition method fails above this temperature.

The data for the control yield (no TMEDA) and for the TMEDA inhibition at 60 °C were taken from our previous publication (Table 3) (6). The same procedure was used to investigate TMEDA inhibition at 80 °C.

Pinacolborane **3b** (0.22 mL, 1.5 mmol), 'catalyst' (0.1 mmol) and TMEDA (0.015 mL, 0.10 mmol) were added to a reaction vial under an argon atmosphere. Phenylacetylene **6** (0.11 mL, 1.0 mmol) was added and the reaction was stirred at 80 °C for 4 hours. The reaction was cooled to room temperature and quenched with an internal standard solution [1,3,5-trimethoxybenzene (0.1 mmol) in CH_2Cl_2 (1.0 mL)]. The yield of the alkenyl boronic ester **4** was determined by ^1H NMR spectroscopy in $CDCl_3$ by comparison of diagnostic peak [6.18 (d, J = 18 Hz, 1H)] with the internal standard [6.09 (s, 3H)]. See Fig. 28 for exemplar ^1H NMR spectrum with diagnostic peaks of key species labelled. The crystal violet **1** indicator turned from purple to colourless in all cases and, therefore, successfully detected the presence of BH_3 (Table 3).

Table 3: TMEDA inhibition method versus crystal violet **1** colourimetric method to detect hidden BH_3-catalysis for the hydroboration of phenylacetylene **6** with HBpin **3b** ('Catalyst'-mediated decomposition of HBpin **3b** to BH_3).

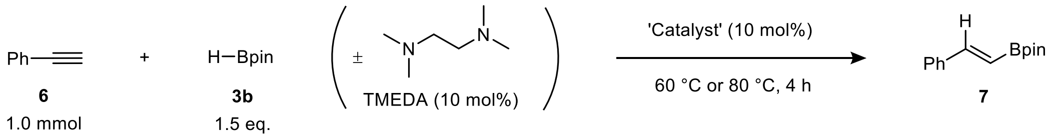

Entry	'Catalyst'	Control Yield (%)	TMEDA Inhibition Yield (%)		Crystal Violet Indicator Colour Change[c]
		60 °C	60 °C	80 °C	80 °C (Yes/No)
1	NaOH	41	1	7	Yes
2	NaO^{t}Bu	59 [a]	1 [a]	77 [a]	Yes 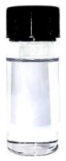
3	Na[N(SiMe$_3$)$_2$]	29	0	49	Yes
4	^{n}BuLi	52	1 [b]	27 [b]	Yes
5	Ti(O^{i}Pr)$_4$	51	5 [b]	39 [b]	Yes

False Negative Result

Hidden Catalysis Identified

3.23. Crystal Violet Indicator Strips

3.23.1. Preparation of the Test Strips

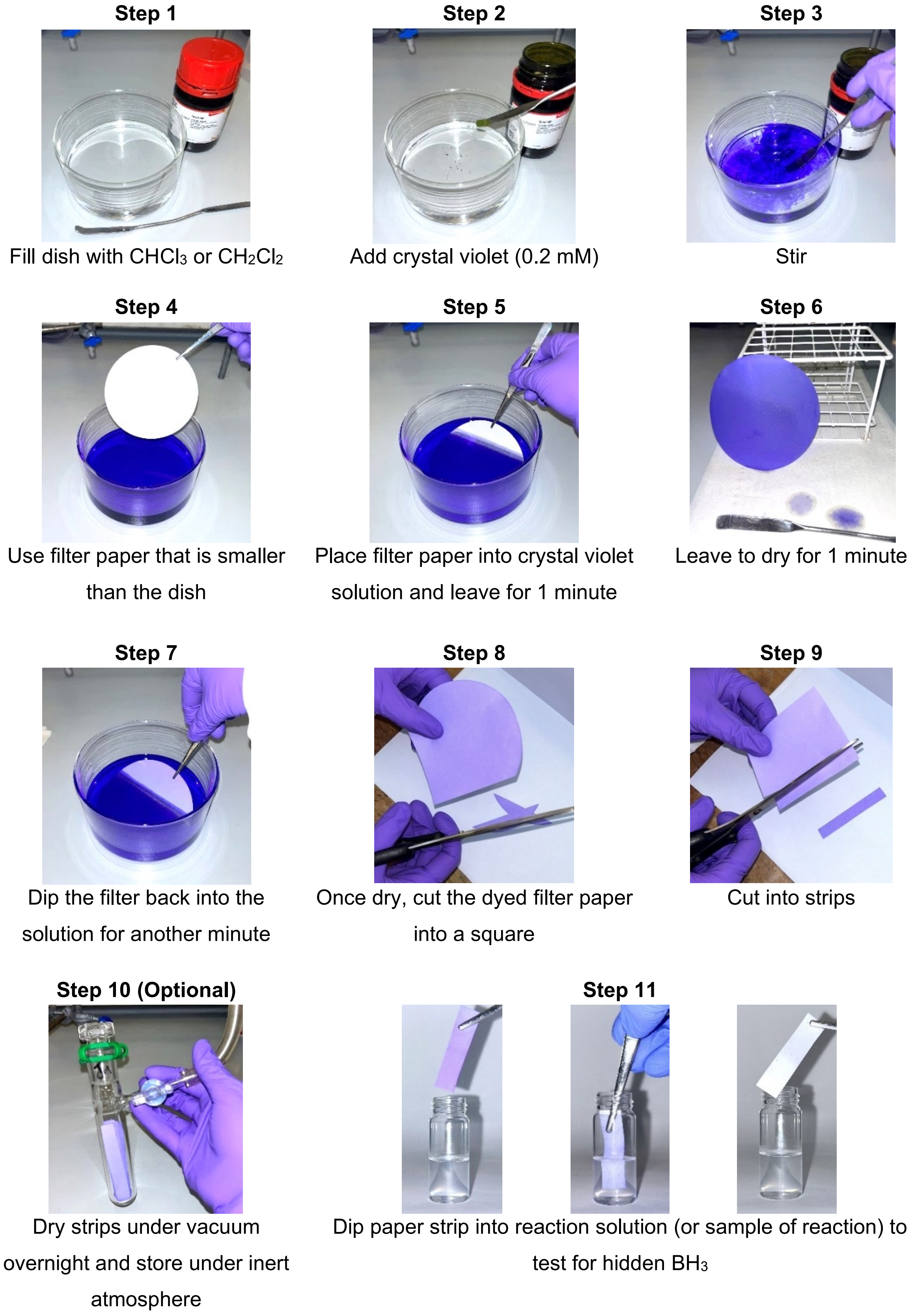

Fig. 31: Procedure for preparing the crystal violet **1** indicator test strips.

3.23.2. Using the Test Strips to Detect Hidden Borane Catalysis

A selection of reactions carried out in *Section 3.17* were also tested using the crystal violet **1** paper strips to detect hidden BH_3 generated by 'catalyst'-mediated decomposition of HBpin **3b** (see Table 4). The test paper strips successfully detected hidden BH_3 and the results are consistent with that observed in Table 2.

Table 4: Using the crystal violet **1** test strips to detect hidden BH_3 generated from 'catalyst'-mediated decomposition of HBpin **3b**.

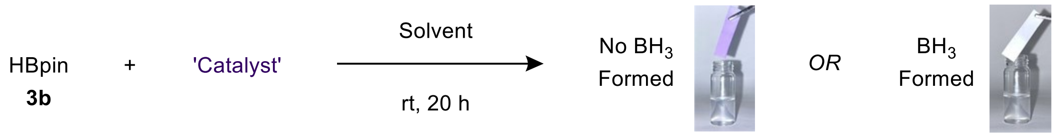

Entry	Catalyst	Solvent	Crystal Violet Paper	Outcome
**1 Control**	HBpin (+BH_3)	THF	White	BH_3 present
		CH_2Cl_2	White	BH_3 present
		PhCl	White	BH_3 present
		Toluene	White	BH_3 present
**2**	PMe_3	Toluene	White	BH_3 present
**3**	CsF	THF	White	BH_3 present
**4**	NaO^{t}Bu	$CHCl_3$	White	BH_3 present
**5**	K_2CO_3	DCM	White	BH_3 present
**6**	P_1-*t*-Bu	Neat	White (CH_2Cl_2 added)	BH_3 present
**7**	[Ir(cod)OMe]$_2$	DCM	Purple	No BH_3
**8**	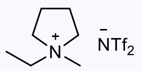	Neat	Purple	No BH_3
**9**	$CaCl_2$	Neat	Purple	No BH_3
**10**	Co(acac)$_2$	THF	Purple	No BH_3
**11**	Co(acac)$_2$ + Xantphos	THF	Purple	No BH_3
**12**	Ni(acac)$_2$	THF	Purple	No BH_3
**13**	Zn(OTf)$_2$	Toluene	Purple	No BH_3

3.24. Using TRIGIT for Colourimetric Analysis

Trigit (http://trigit.com.au) was used for colourimetric analysis of images (*41, 42*) to extract colourimetric data and RGB (Red Green Blue) values (Fig. 32, 33 and 34). A change from any saturation of purple (see Fig. 32, 33 and 34 for exemplar values) to white (hex #ffffff) indicates a colour change (purple to colourless/white) and positive identification of BH_3.

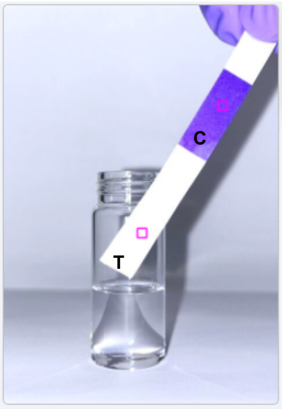

	ID	µR	µG	µB	%R	%G	%B	σR	σG	σB	µH	µS	µV	px	hex	—
	Control	122.75	65.97	249.93	0.28	0.15	0.57	9.30	9.38	6.56	258.52	73.60	98.01	256	#7b42fa	—
	Test Strip	255.00	255.00	255.00	0.33	0.33	0.33	0.00	0.00	0.00	137.28	0.00	100.00	256	#ffffff	—

Fig. 32: TRIGIT colour analyses of the control (purple, hex #7b42fa) and test (white, hex #ffffff) sections of the indicator paper strip. The test section was dipped into a solution containing BH_3 (X M $Me_2S{\cdot}BH_3$ in CH_2Cl_2) resulting in decolourisation of the dipped section of the strip (purple to white).

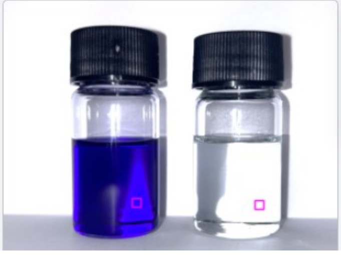

	ID	µR	µG	µB	%R	%G	%B	σR	σG	σB	µH	µS	µV	px	hex	—
	True Catalysis	43.55	0.86	254.72	0.15	0.00	0.85	3.17	0.74	0.51	250.09	99.66	99.89	4096	#2c01ff	—
	Hidden Catalysis	255.00	255.00	255.00	0.33	0.33	0.33	0.00	0.00	0.00	137.28	0.00	100.00	4096	#ffffff	—

Fig. 33: TRIGIT colour analyses of a purple (hex #2c01ff) crystal violet **1** solution (5 mL, 0.2 mM, 1 µmol, $CHCl_3$) and colourless (white, hex #ffffff) crystal violet **1** + BH_3 solution (5 mL, 0.2 mM, 1 µmol, $CHCl_3$ containing $Me_2S{\cdot}BH_3$ 0.20 mL, 2.1 mmol).

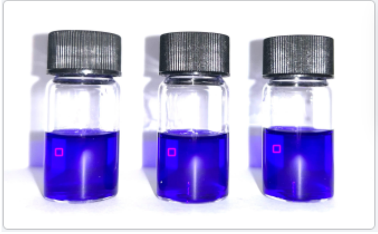

	ID	µR	µG	µB	%R	%G	%B	σR	σG	σB	µH	µS	µV	px	hex	—
		48.23	1.88	254.83	0.16	0.01	0.84	1.87	0.89	0.45	250.99	99.26	99.93	2304	#3002ff	—
		47.70	1.51	254.61	0.16	0.00	0.84	2.34	1.06	0.59	250.95	99.41	99.85	2304	#3002ff	—
		29.33	0.85	254.78	0.10	0.00	0.89	3.44	1.04	0.58	246.73	99.67	99.91	2304	#1d01ff	—

Fig. 34: TRIGIT colour analyses of three crystal violet **1** solutions (4 mL, 0.2 mM, 0.8 µmol, CH_2Cl_2) containing boron reagents (0.8 mmol, 1000 equivalents); boric acid (LEFT, purple, hex #3002ff), trimethyl borate (MIDDLE, purple, hex #3002ff) and trimethylboroxine (RIGHT, purple, hex #1d01ff).

3.25. Other Indicators

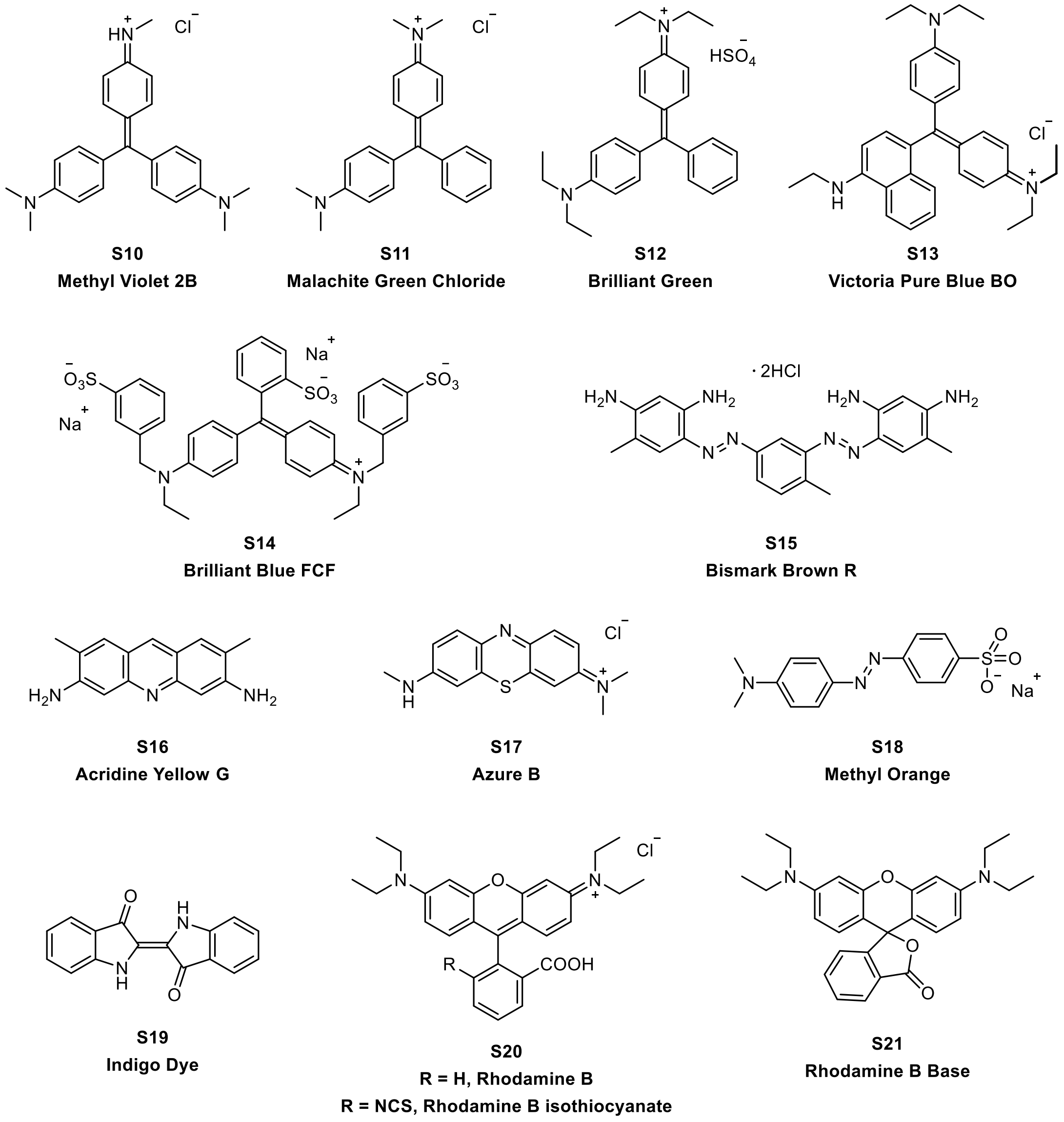

Fig. 35: Structures of other indicators that were investigated as potential indicators for hidden borane catalysis.

4. References

37. Camacho, C., Paz-Sandoval, M. A. & Contreras, R. Studies on Aromatic Amine Boranes by ^{11}B and ^{1}H NMR. *Polyhedron* **5**, 1723–1732 (1986).

38. Brown, H. C. & Gupta, S. K. Hydroboration. XXXIX. 1,3,2-Benzodioxaborole (catecholborane) as a New Hydroboration Reagent for Alkenes and Alkynes. *J. Am. Chem. Soc.* **97**, 5249–5255 (1975).

39. Kanno, S., Kakiuchi, F. & Kochi, T. Palladium-Catalyzed Hydroboration/Cyclization of 1,*n*-Dienes. *J. Org. Chem.* **88**, 2621–2630 (2023).

40. Davis, M., Deady, L. W., Finch, A. J. & Smith, J. F. Some Reactions of Grignard Reagents with Chloroform and Carbon Tetrachloride in the Presence of Cyclohexene. *Tetrahedron* **29**, 349–352 (1973).

41. Tjandra A. D., Heywood T. & Chandrawati R. Trigit: A Free Web Application for Rapid Colorimetric Analysis of Images. *Biosensors and Bioelectronics: X* **14**, 100361–100365 (2023).

42. TRGIT Color in Digits, https://trigit.com.au/, (Accessed April 2025).

5. NMR Spectra

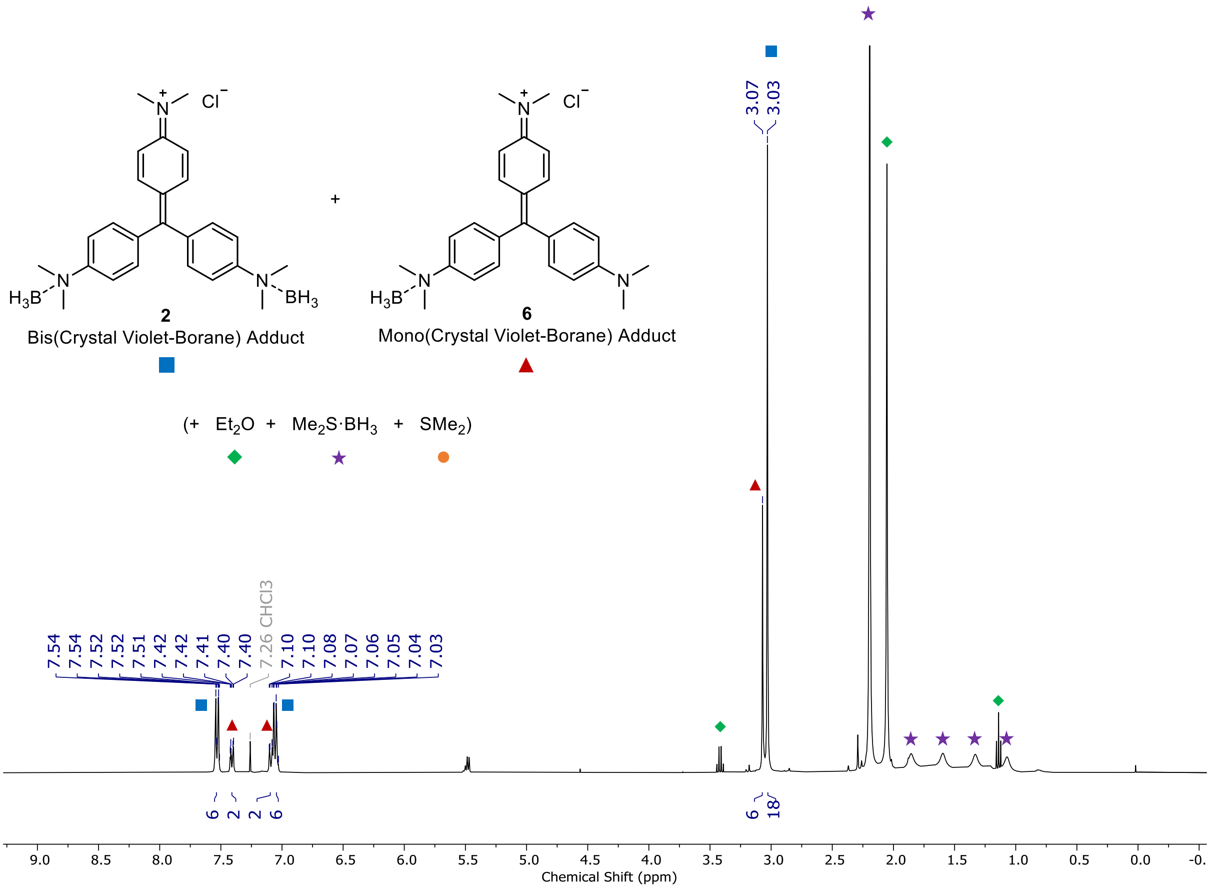

Fig. 36: ^{1}H NMR spectrum (400 MHz, $CDCl_3$) of a solution containing Crystal Violet·$(BH_3)_2$ **2**, Crystal Violet·BH_3 **S6**, $Me_2S{\cdot}BH_3$, SMe_2 and Et_2O in $CDCl_3$.

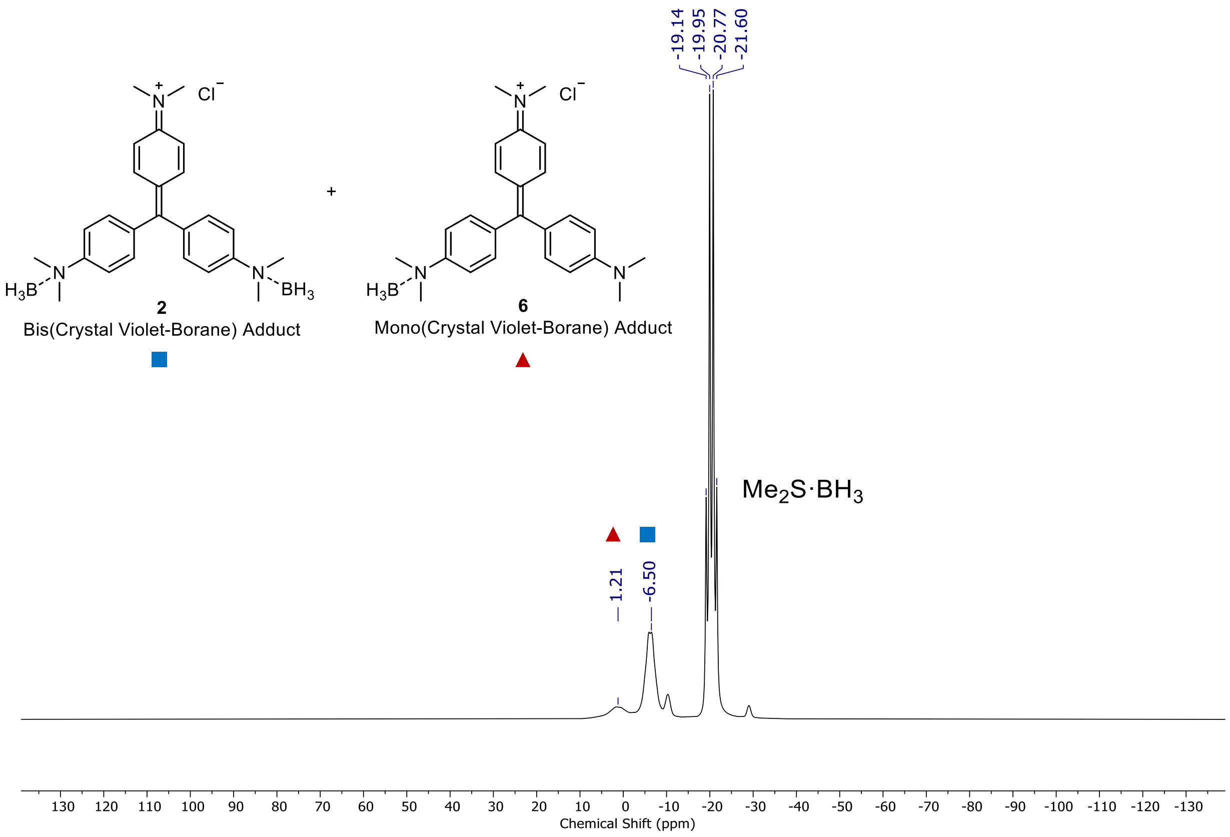

Fig. 37: ^{11}B NMR spectrum (128 MHz, $CDCl_3$) of a solution containing Crystal Violet·$(BH_3)_2$ **2**, Crystal Violet·BH_3 **S6** and $Me_2S·BH_3$.

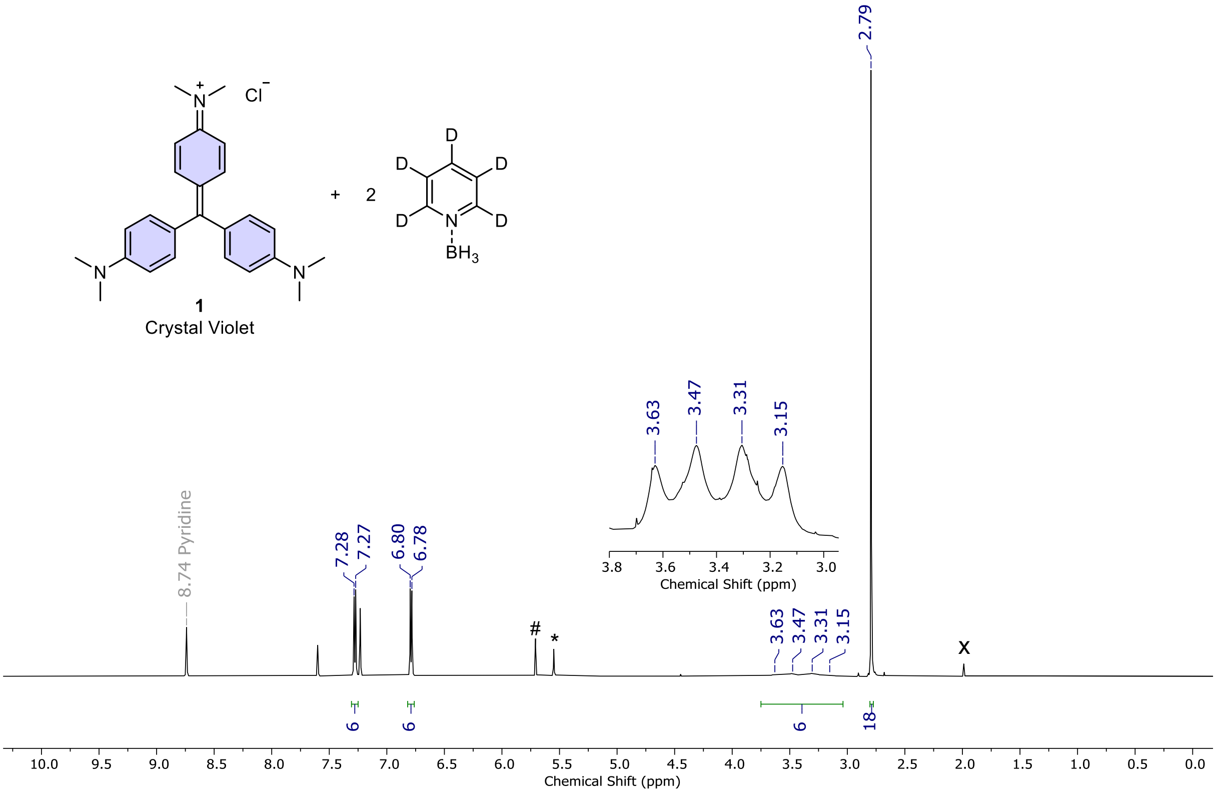

Fig. 38: ^{1}H NMR spectrum (500 MHz, d_5-pyridine) of crystal violet·$(BH_3)_2$ **2** precipitate in d_5-pyridine. The d_5-pyridine preferentially co-ordinates to the BH_3 instead of crystal violet **1**. Therefore d_5-pyridine removes the BH_3 from crystal violet·$(BH_3)_2$ **2** resulting in the formation of the d_5-pyridine·BH_3 **S7** complex and free crystal violet **1**. The signal denoted with X is residual SMe_2. The signal denoted with * is water present in the NMR solvent. The signal denoted with # is residual dichloromethane.

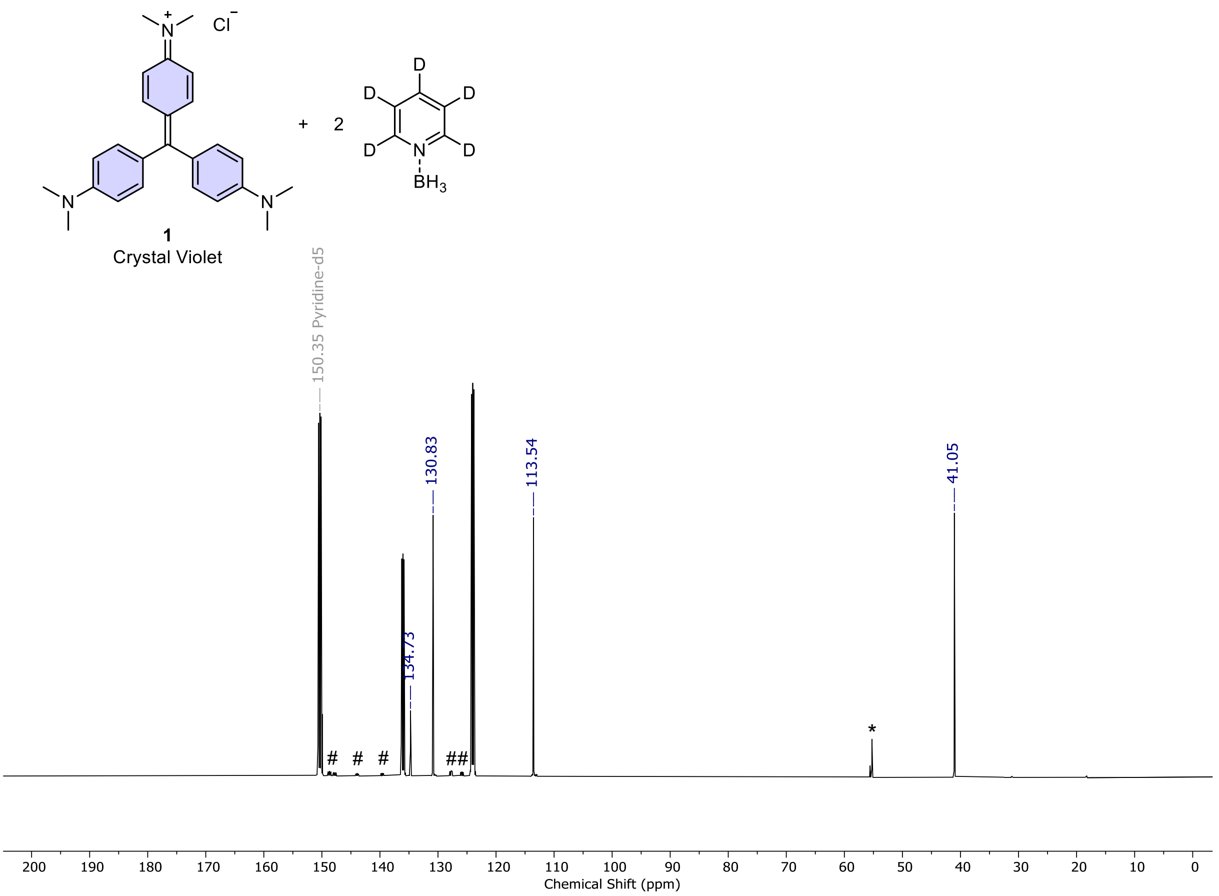

Fig. 39: $^{13}C\{^{1}H\}$ NMR (126 MHz, d_5-pyridine) of crystal violet·$(BH_3)_2$ **2** precipitate in d_5-pyridine. The d_5-pyridine preferentially co-ordinates to the BH_3 instead of crystal violet **1**. Therefore d_5-pyridine removes the BH_3 from crystal violet·$(BH_3)_2$ **2** resulting in the formation of the d_5-pyridine·BH_3 **S7** complex and free crystal violet **1**. The signal denoted with * is residual dichloromethane. The signal denoted with # is pyridine·BH_3.

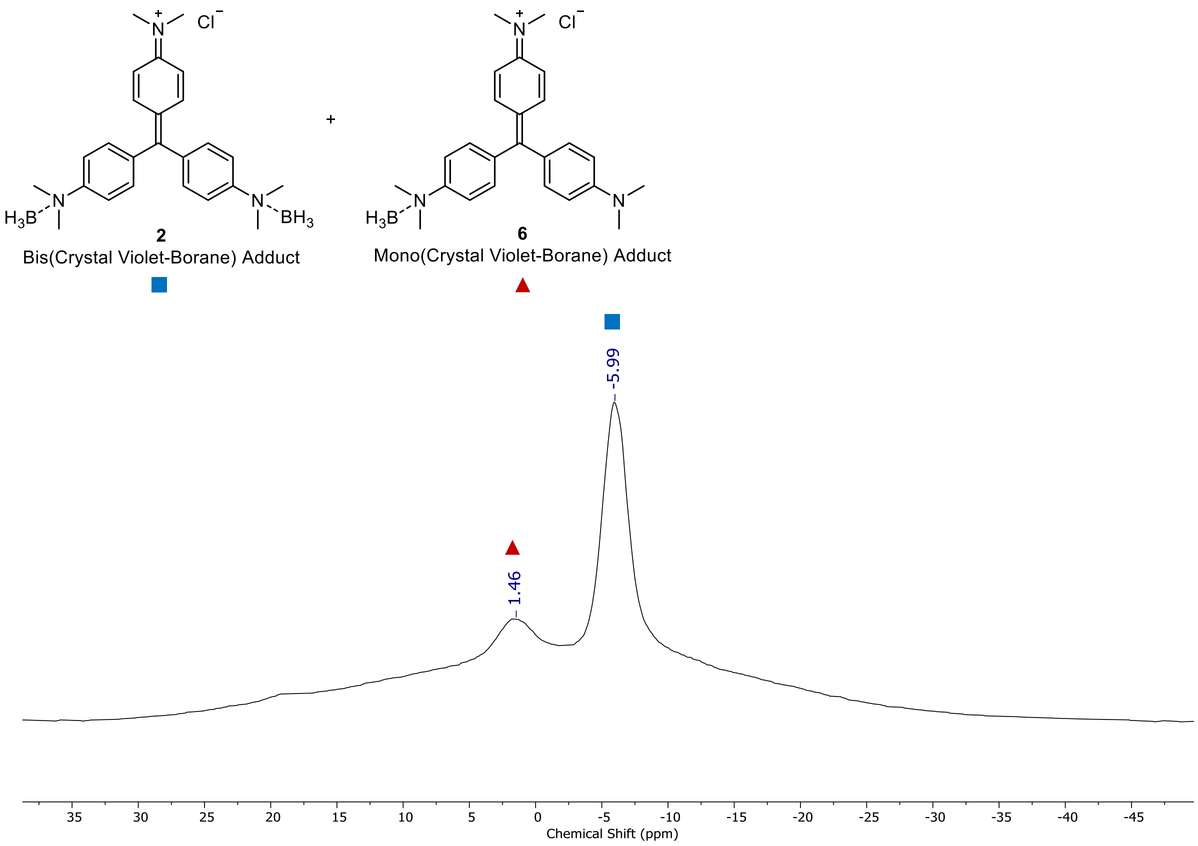

Fig. 40: ^{11}B NMR (128 MHz, $CDCl_3$) of crystal violet-borane **2**. Mono-adduct **S6** (1.46 ppm) and bis-adduct **2** (-5.99 ppm).

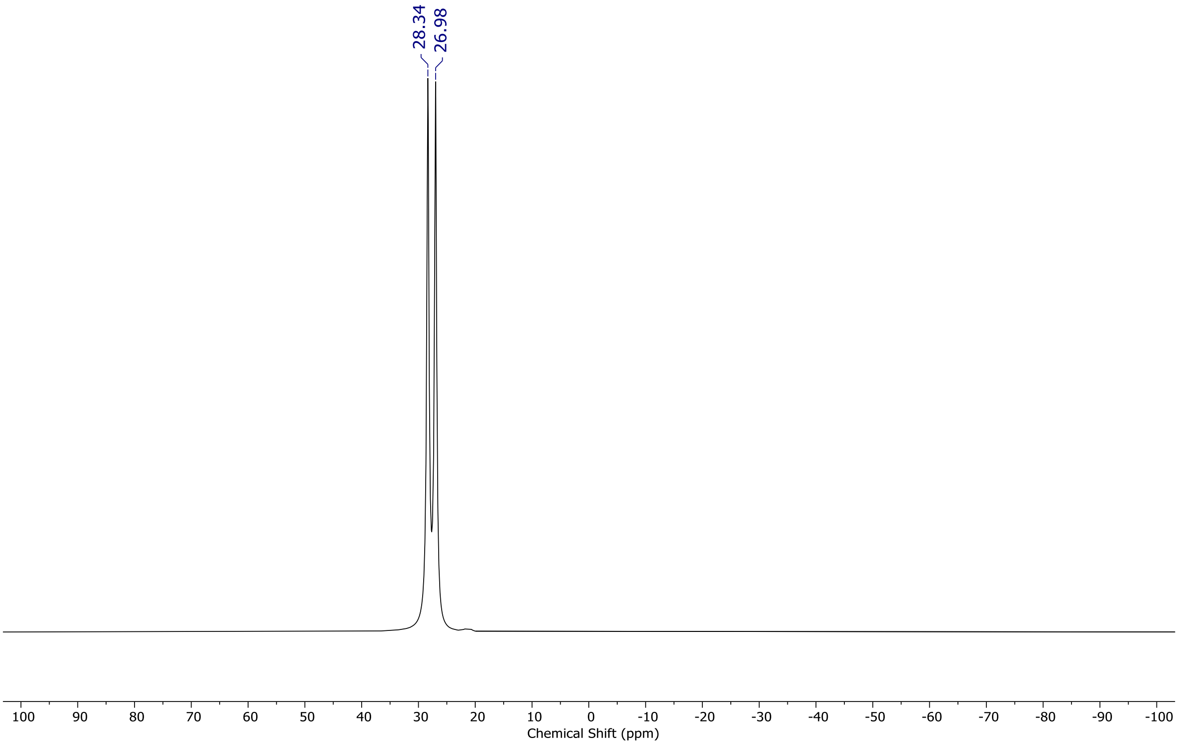

Fig. 41: ^{11}B NMR (128 MHz, $CDCl_3$) of clean HBpin **3b**.

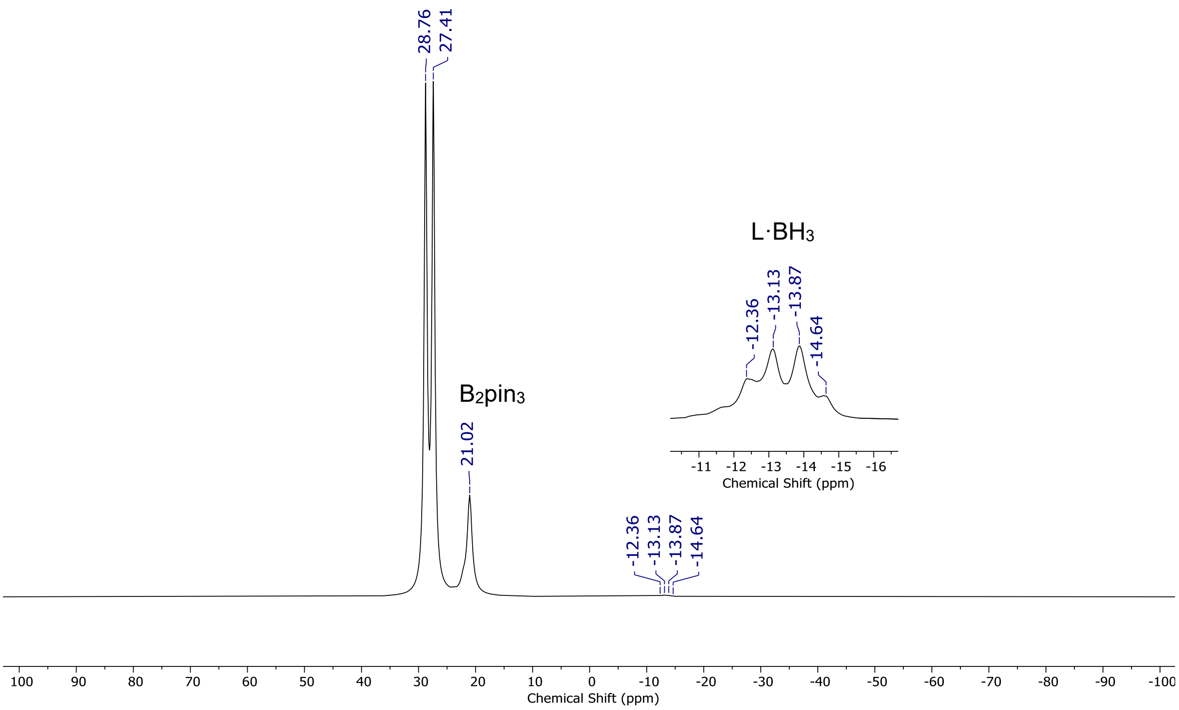

Fig. 42: ^{11}B NMR (128 MHz, $CDCl_3$) of HBpin **3b** containing BH_3 and B_2pin_3 generated from decomposition of HBpin **3b**.

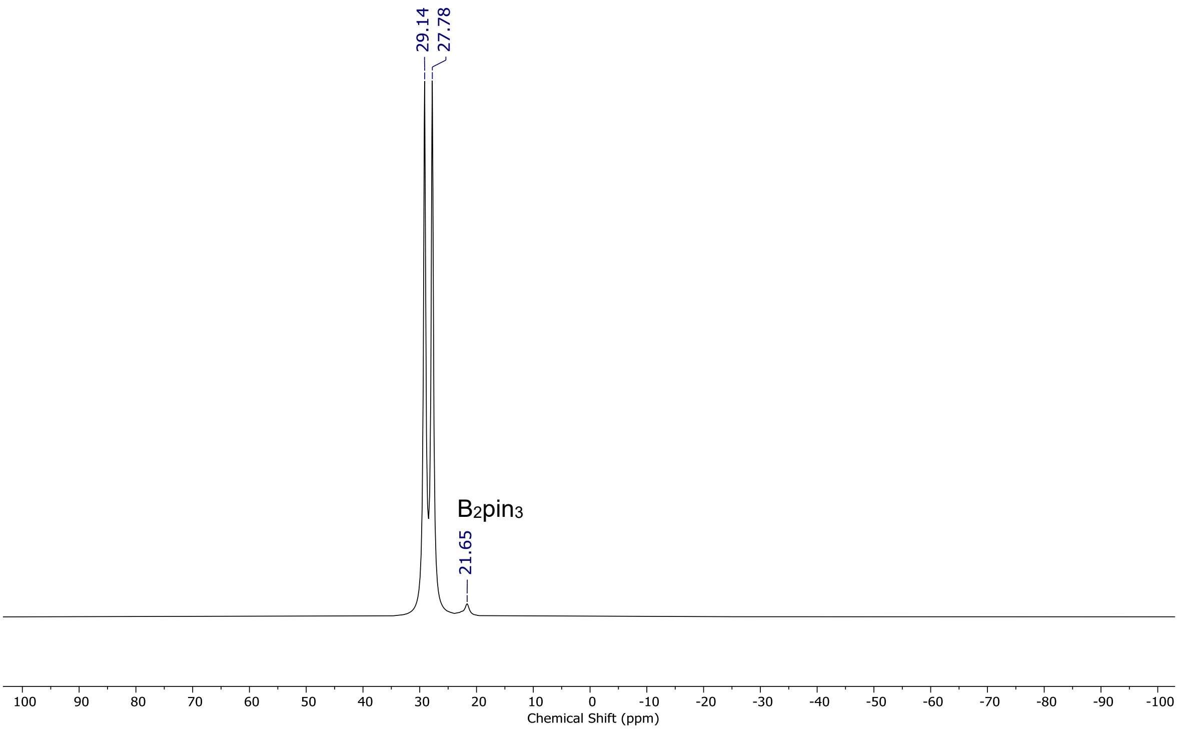

Fig. 43: ^{11}B NMR (128 MHz, h_8-toluene) of NaOTf and HBpin **3b**.

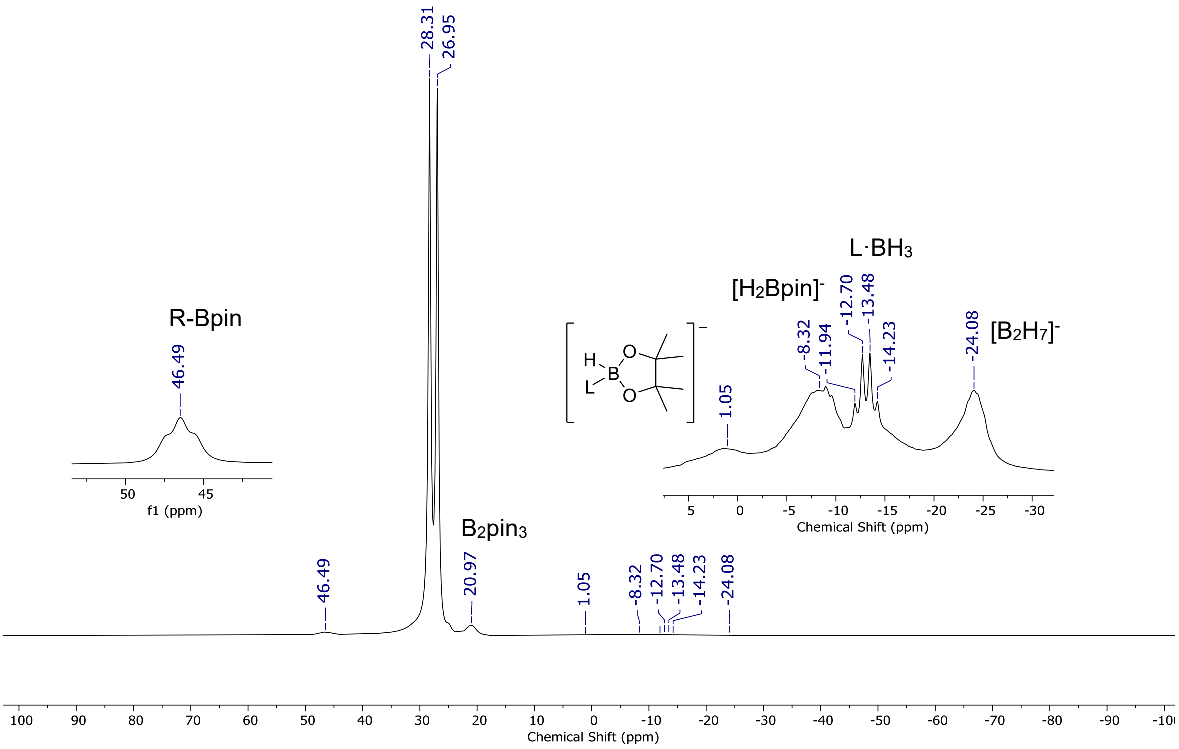

Fig. 44: ^{11}B NMR (128 MHz, h_8-toluene) of $La[N(SiMe_3)_2]_3$ and HBpin **3b**.

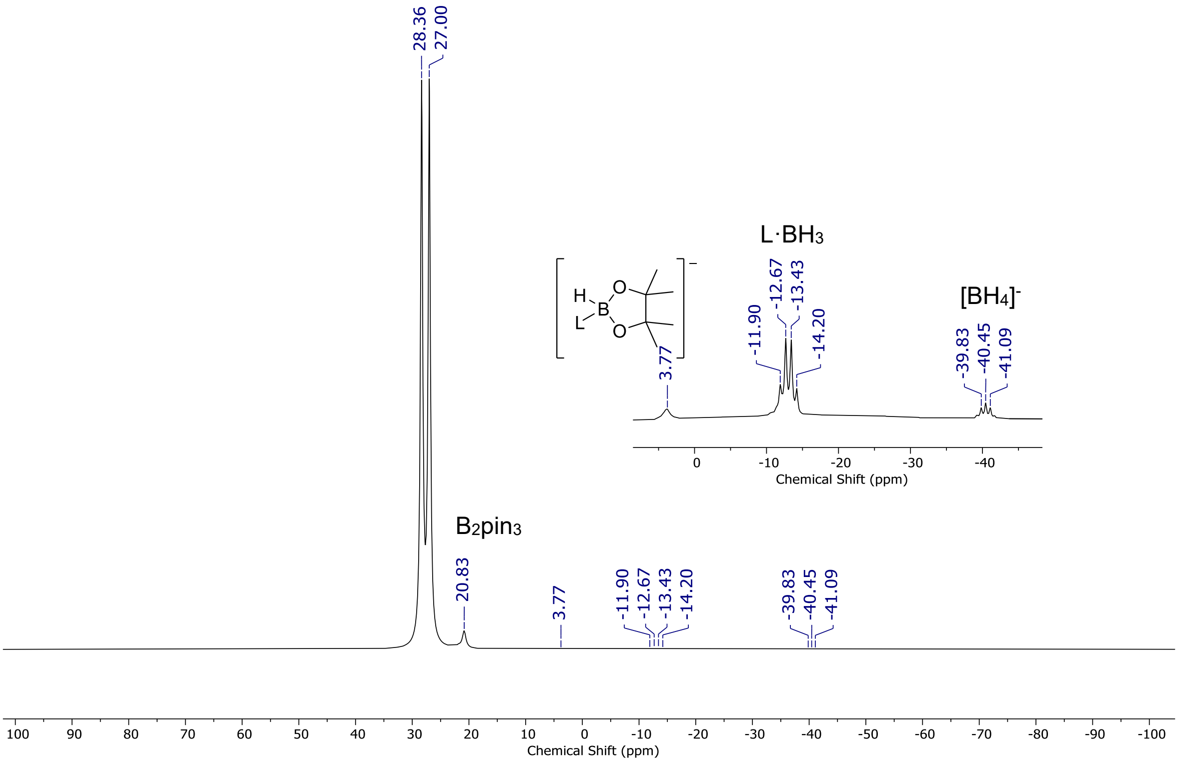

Fig. 45: ^{11}B NMR (128 MHz, h_8-toluene) of LiO^{t}Bu and HBpin **3b**.

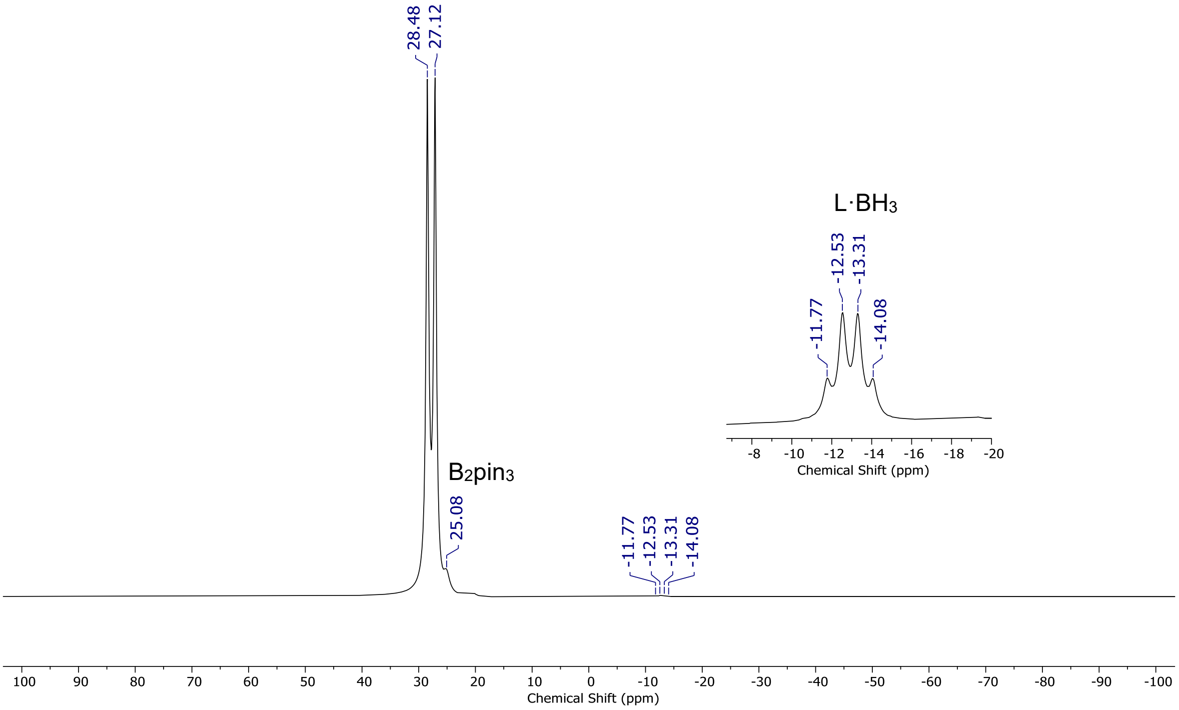

Fig. 46: ^{11}B NMR (128 MHz, h$_8$-toluene) of K[N(SiMe$_3$)$_2$] and HBpin **3b**.

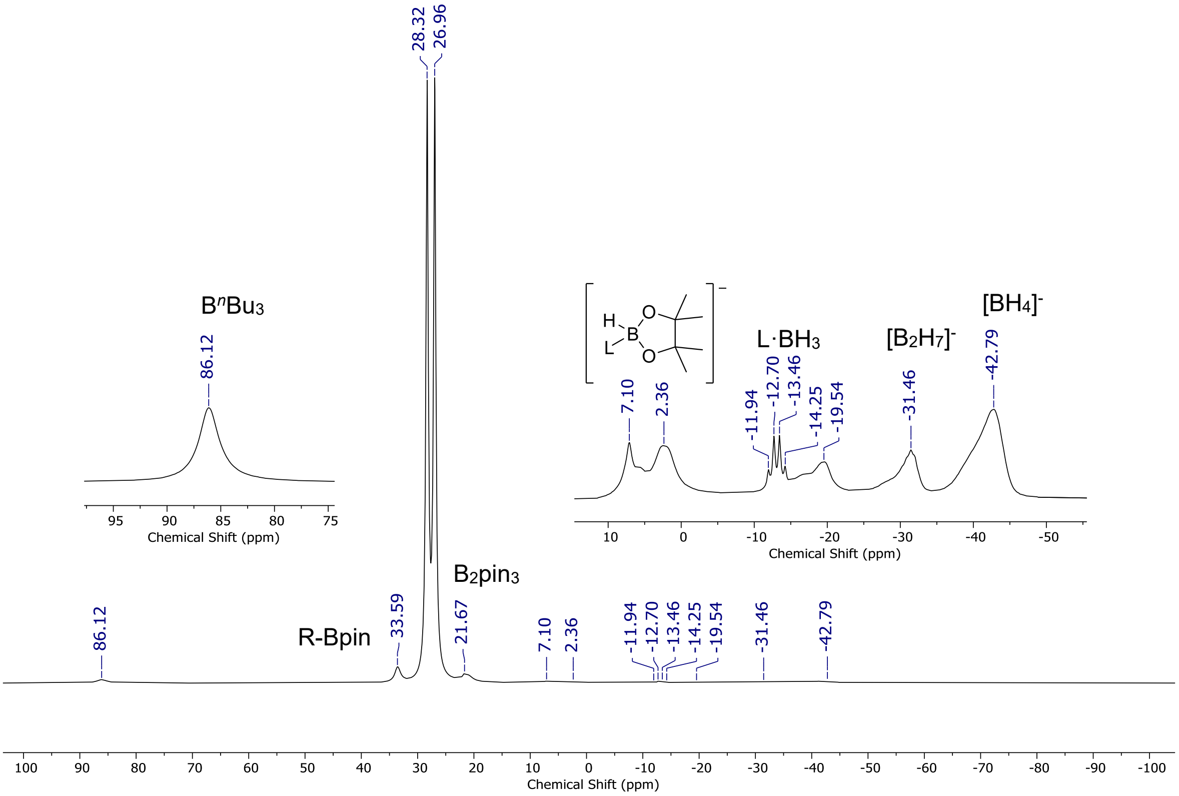

Fig. 47: ^{11}B NMR (128 MHz, h$_8$-toluene) of ^{n}Bu$_2$Mg and HBpin **3b**.

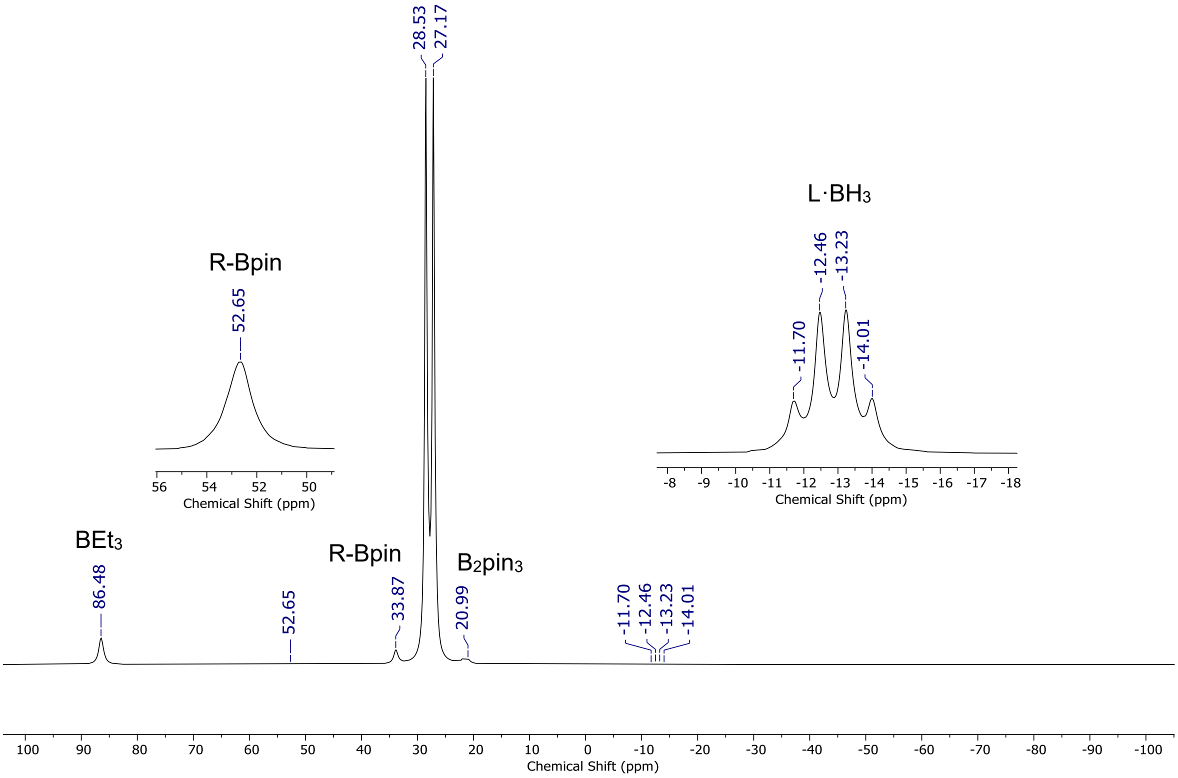

Fig. 48: ^{11}B NMR (128 MHz, h_8-toluene) of BEt_3 and HBpin **3b**.

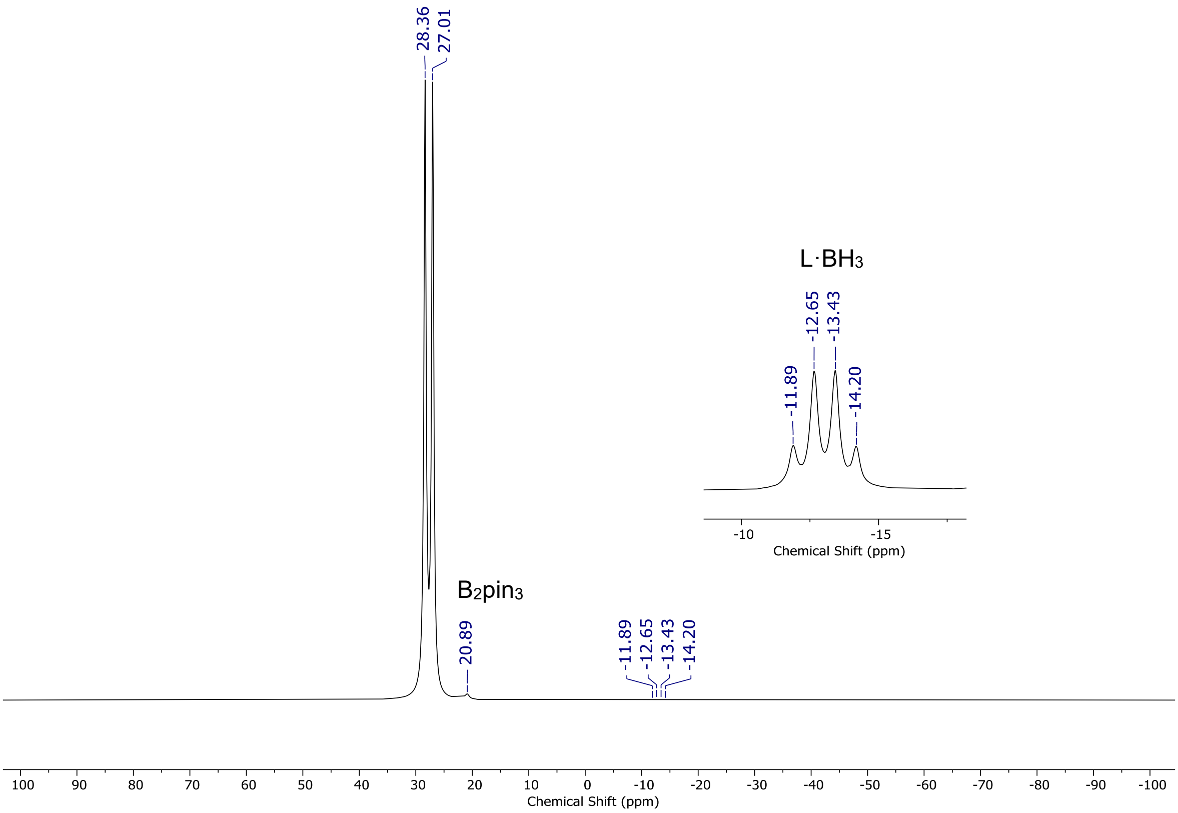

Fig. 49: ^{11}B NMR (128 MHz, h_8-toluene) of $NaBH_4$ and HBpin **3b**.

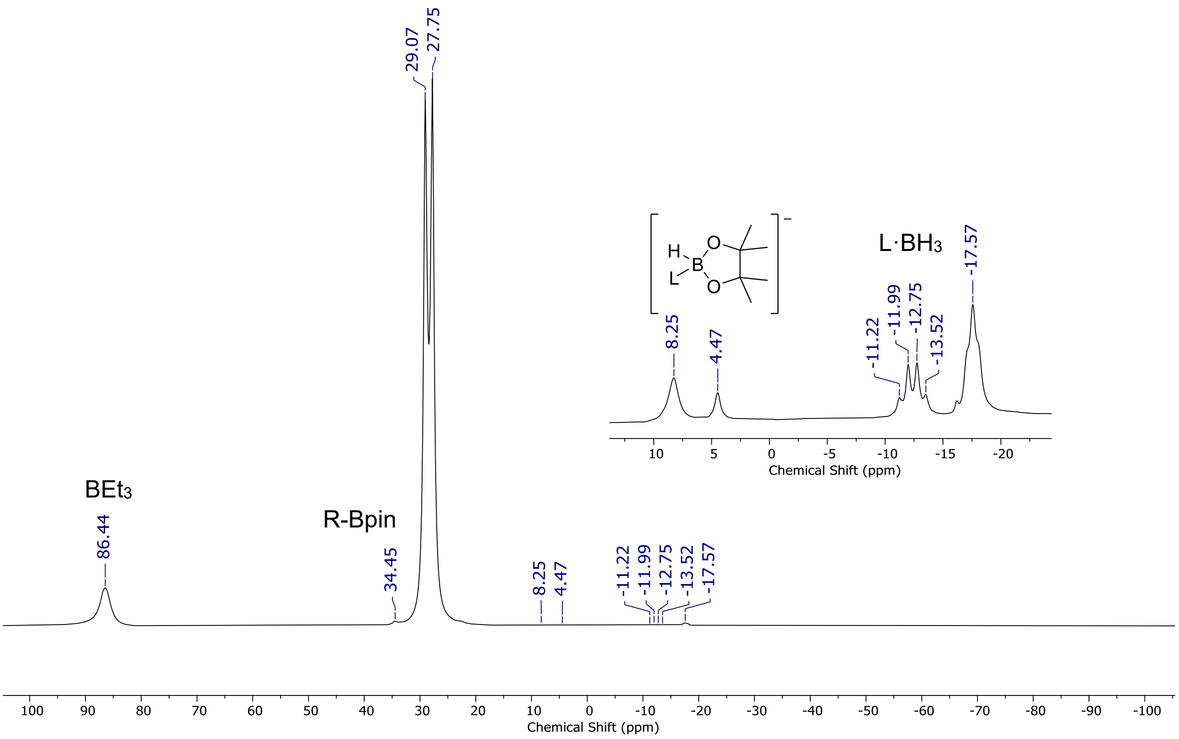

Fig. 50: ^{11}B NMR (128 MHz, h_8-toluene) of $NaHBEt_3$ and HBpin **3b**.

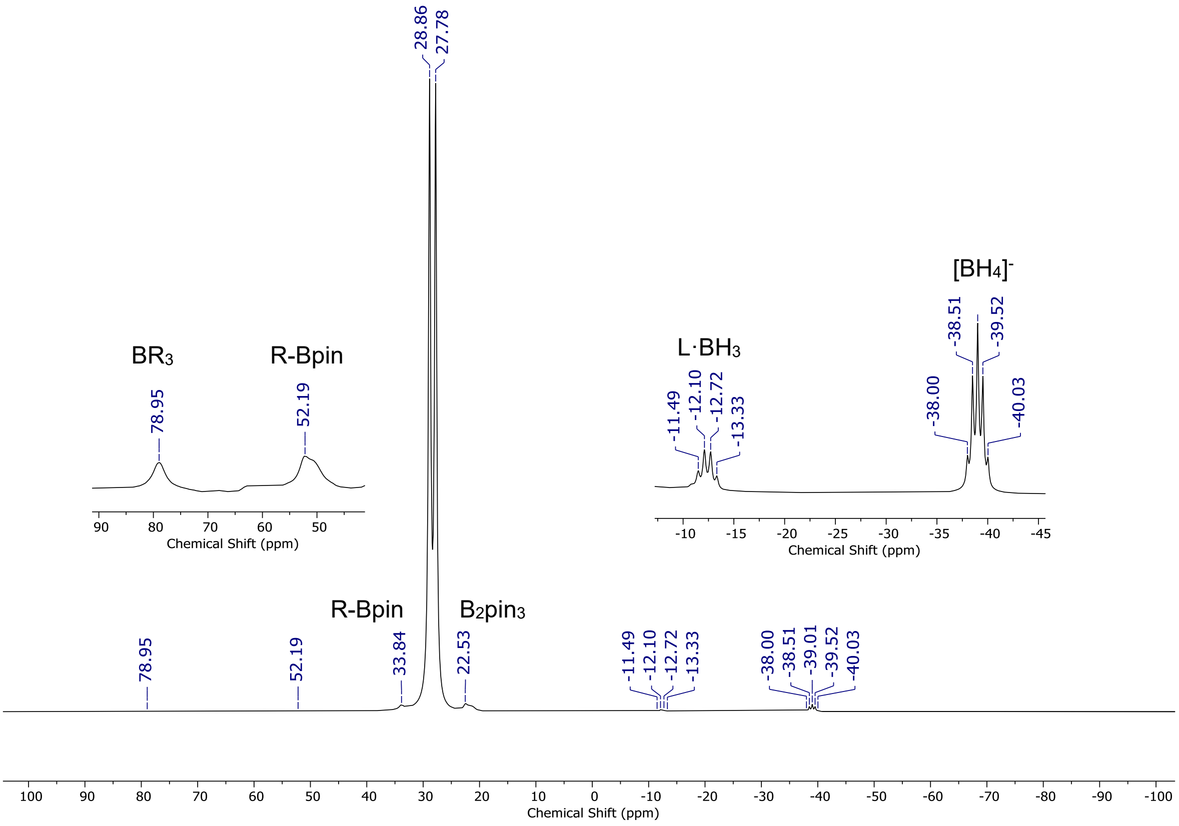

Fig. 51: ^{11}B NMR (128 MHz, h_8-toluene) of $LiCH_2SiMe_3$ and HBpin **3b**.

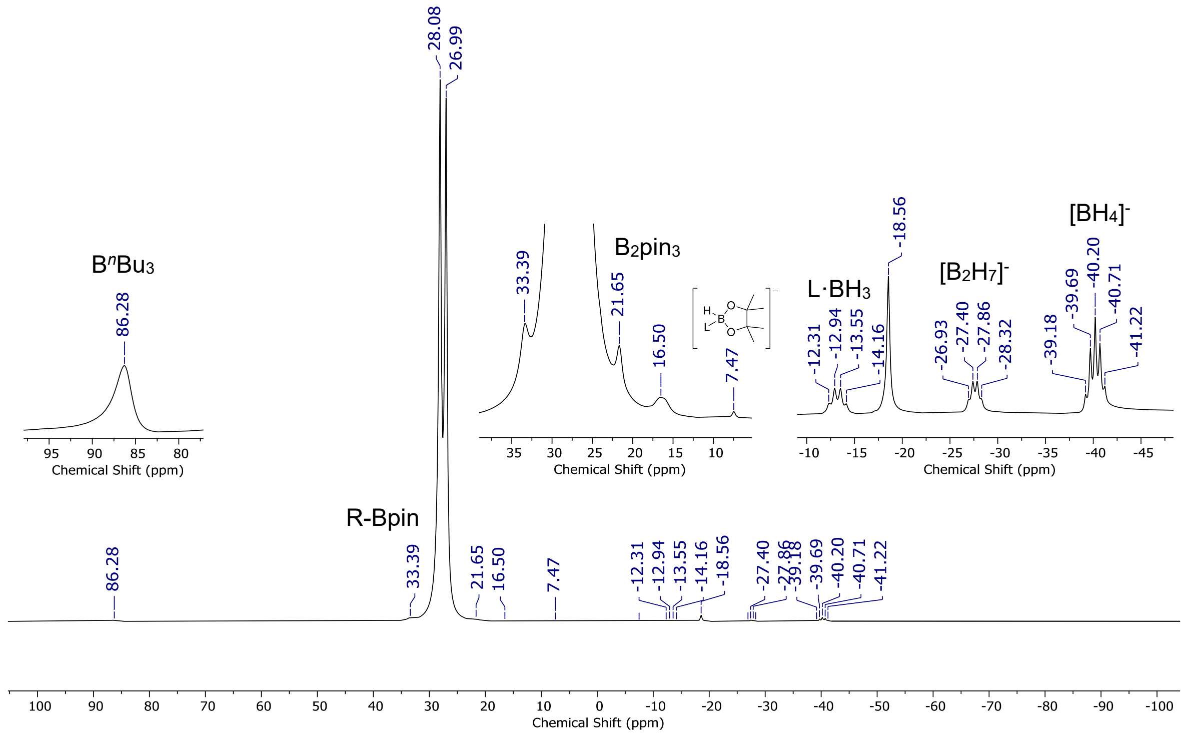

Fig. 52: ^{11}B NMR (160 MHz, h8-toluene) of ^{n}BuLi and HBpin **3b**.

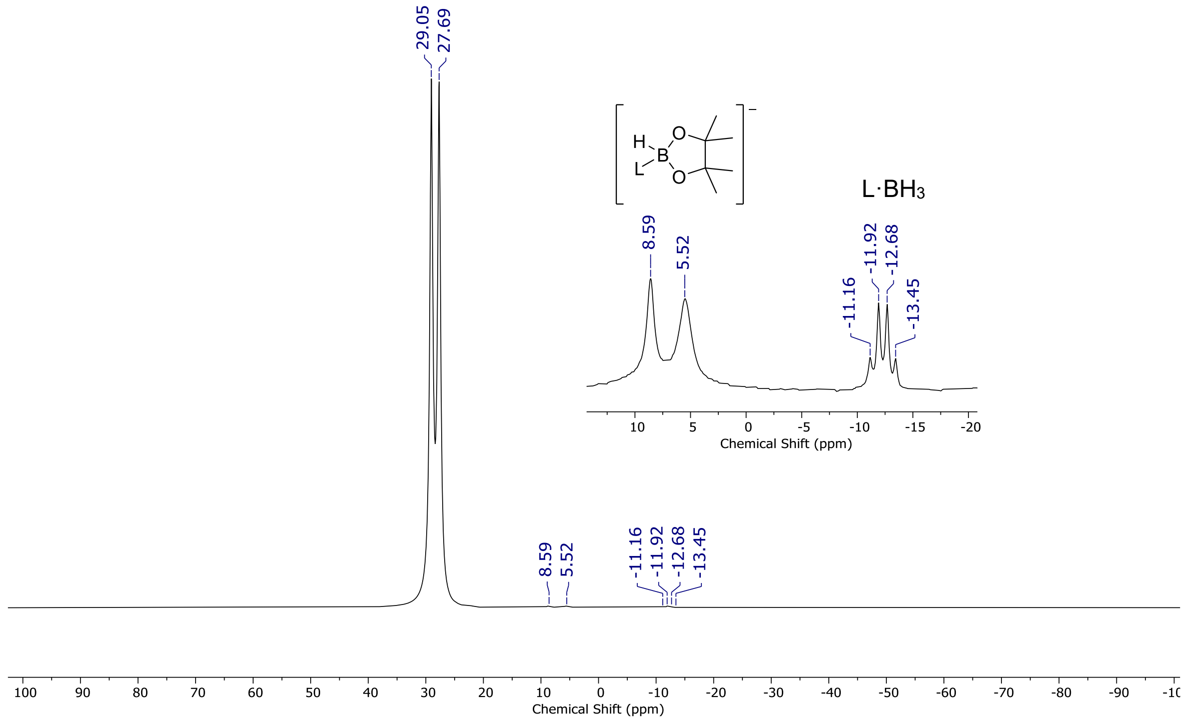

Fig. 53: ^{11}B NMR (160 MHz, h8-toluene) of sodium 2-ethylhexanoate and HBpin **3b**.

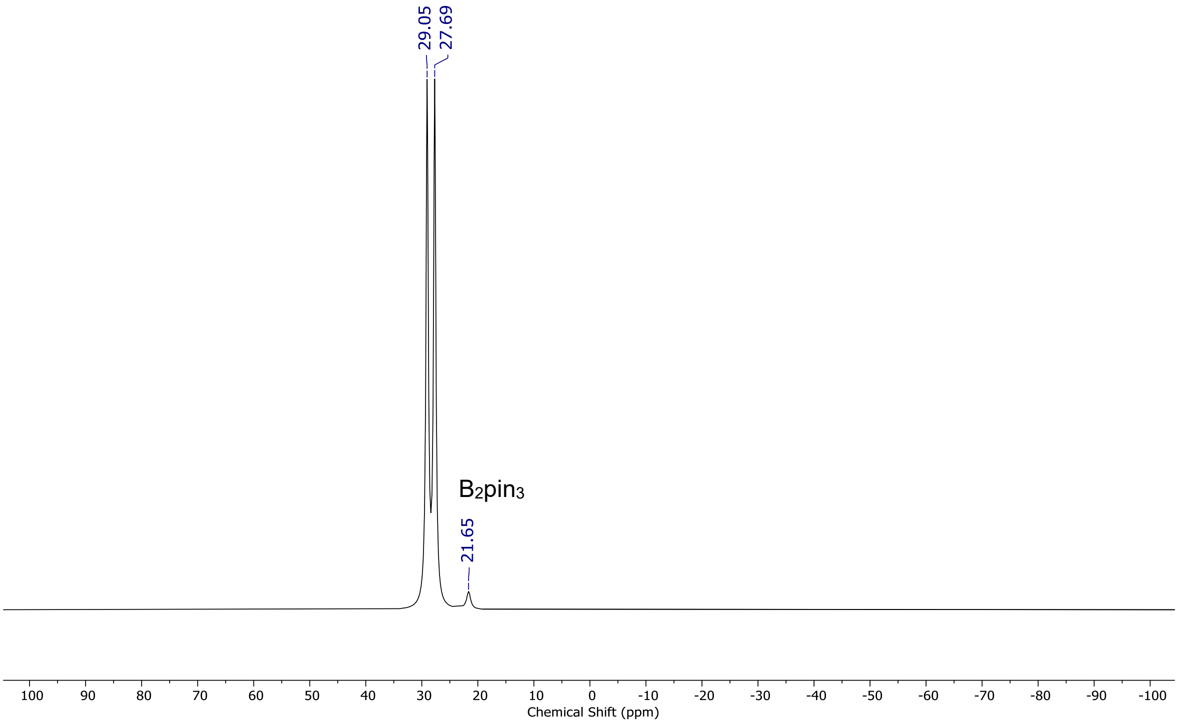

Fig. 54: ^{11}B NMR (128 MHz, h_8-toluene) of $CaCl_2$ and HBpin **3b**.

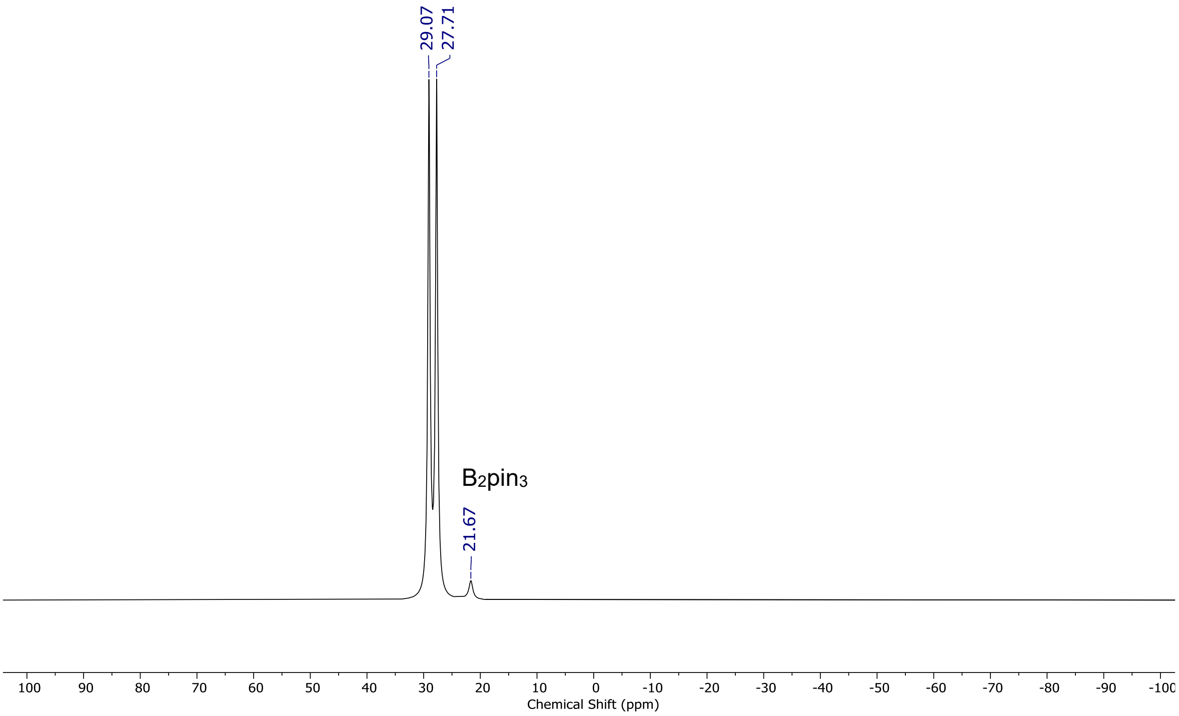

Fig. 55: ^{11}B NMR (128 MHz, CH_2Cl_2) of [**Ir**(cod)OMe]$_2$ and HBpin **3b**.

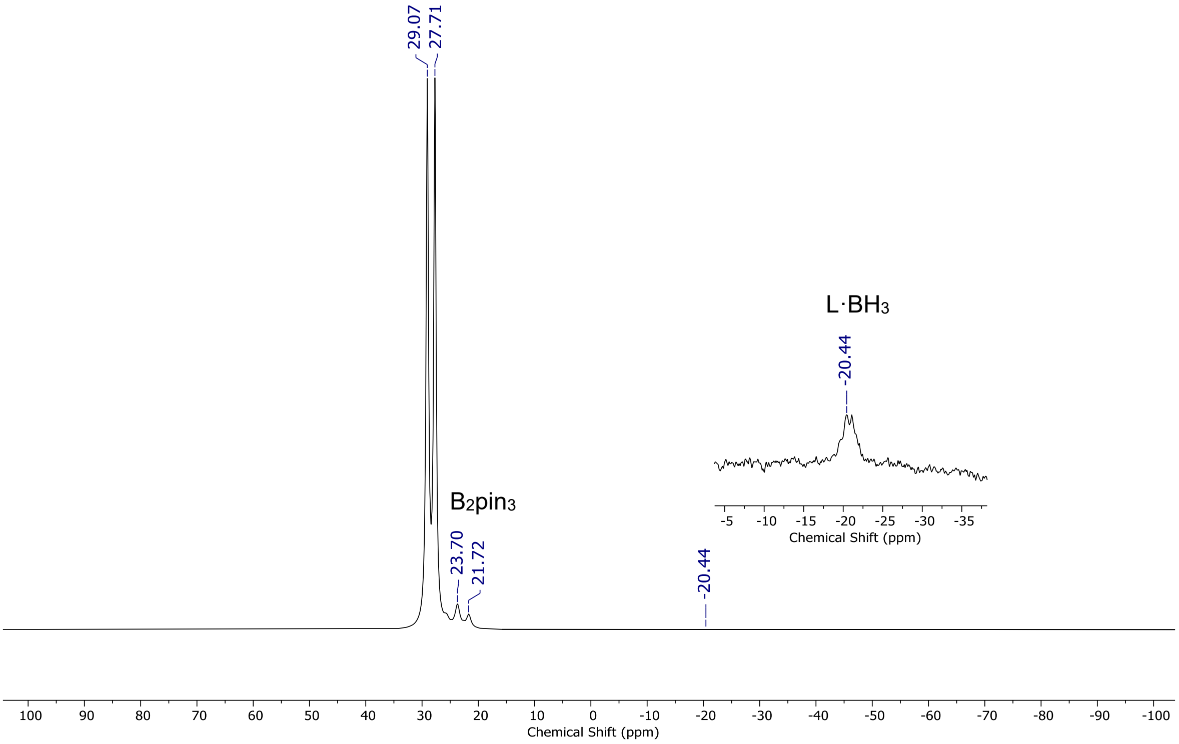

Fig. 56: ^{11}B NMR (128 MHz, h_8-toluene) of Phosphazene base P_1-t-Bu and HBpin **3b**.

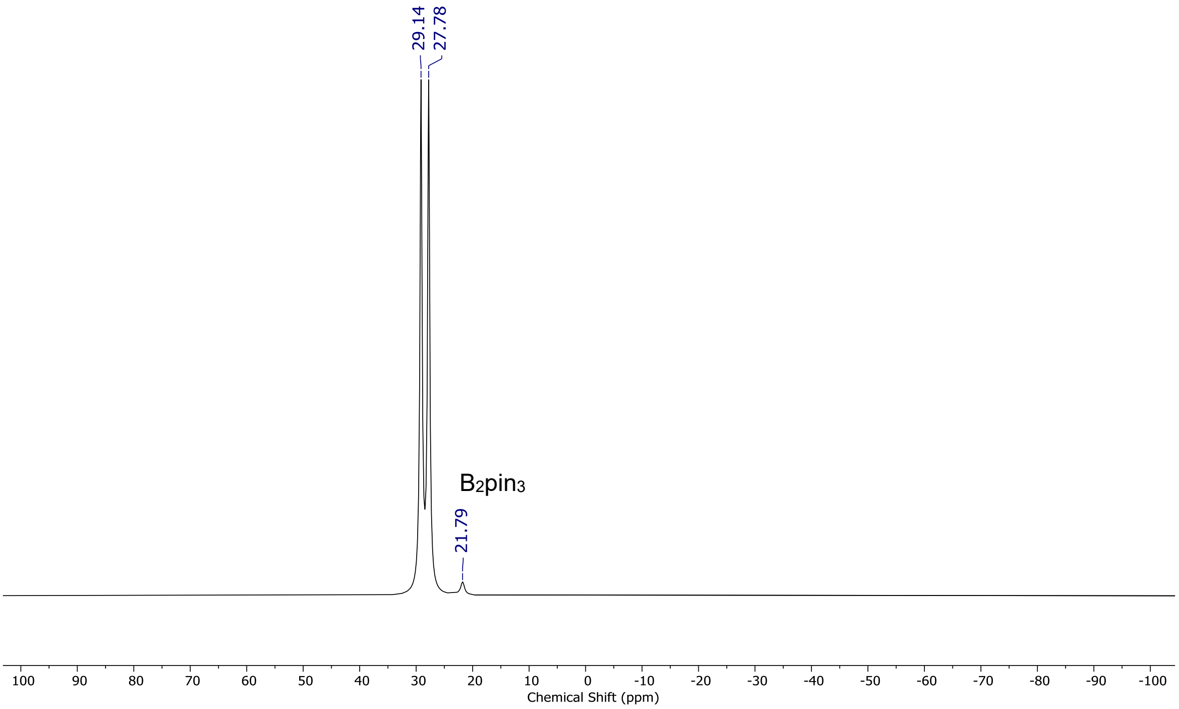

Fig. 57: ^{11}B NMR (128 MHz, h_8-toluene) of 1-Ethyl-1-methylpyrrolidium bis(trifluoro)imide and HBpin **3b**.

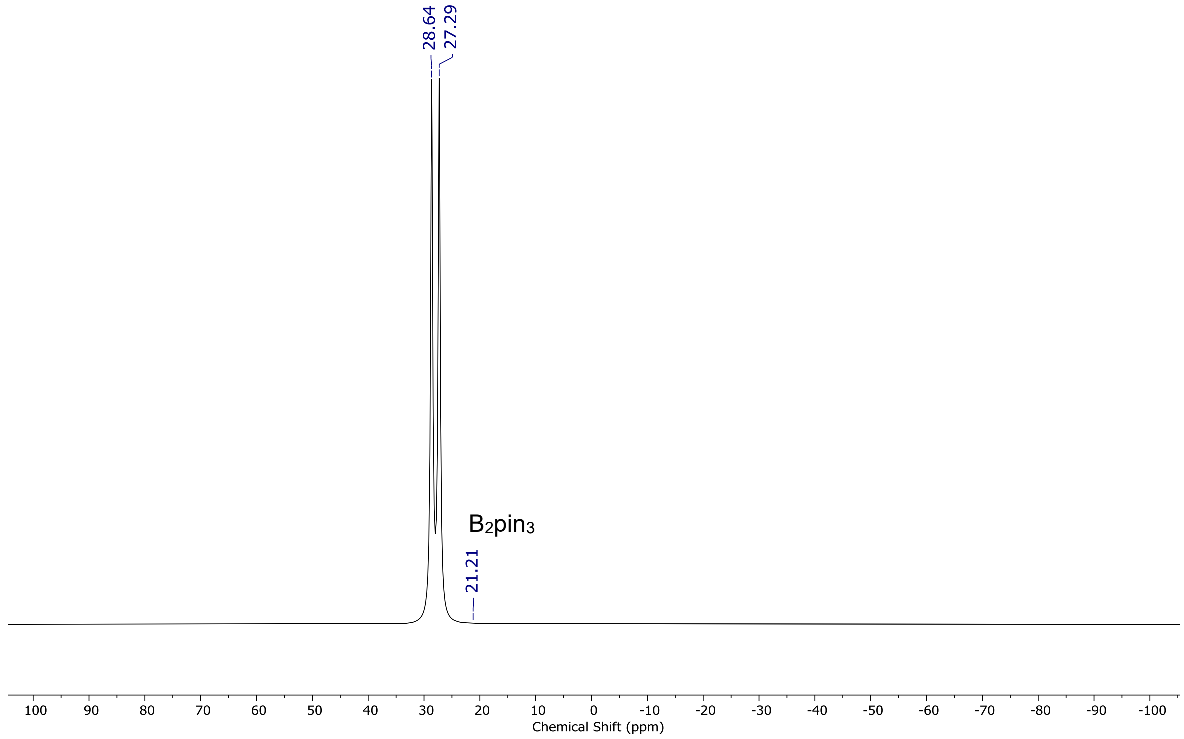

Fig. 58: ^{11}B NMR (128 MHz, h_8-toluene) of $Zn(OTf)_2$ and HBpin **3b**.

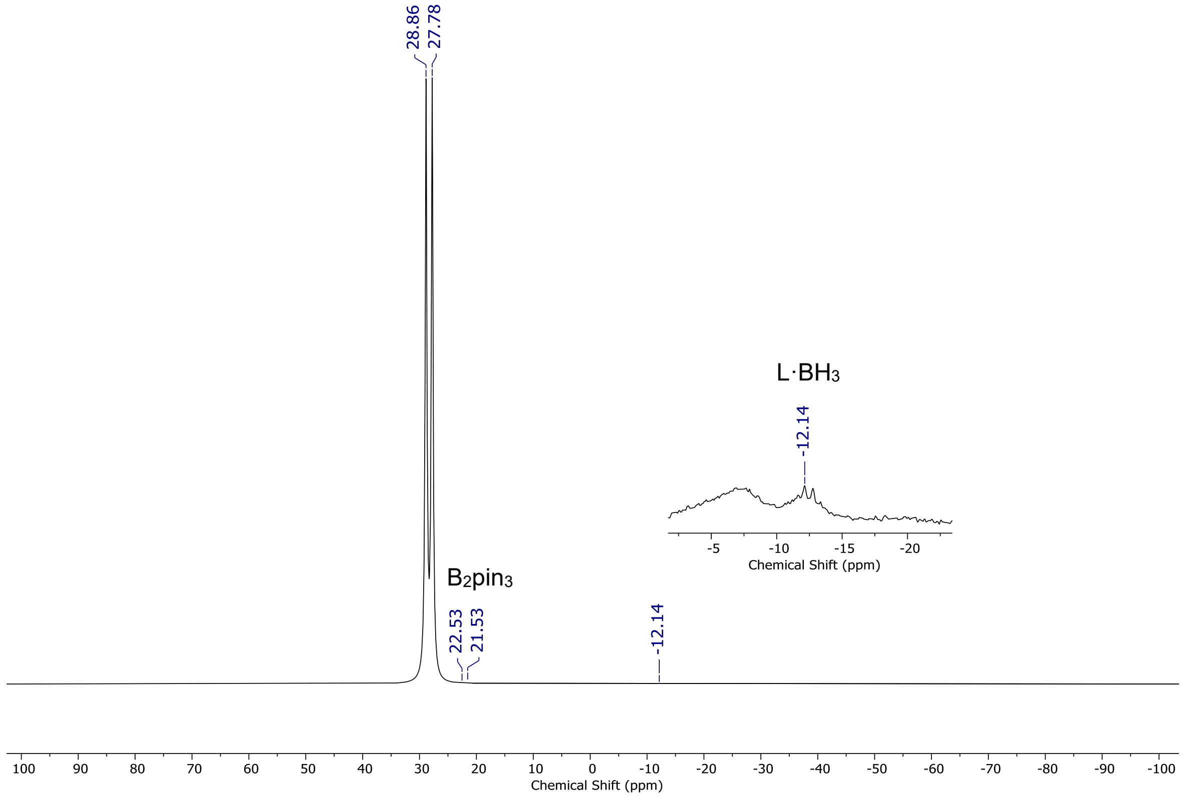

Fig. 59: ^{11}B NMR (160 MHz, h_8-toluene) of PMe_3 and HBpin **3b**.

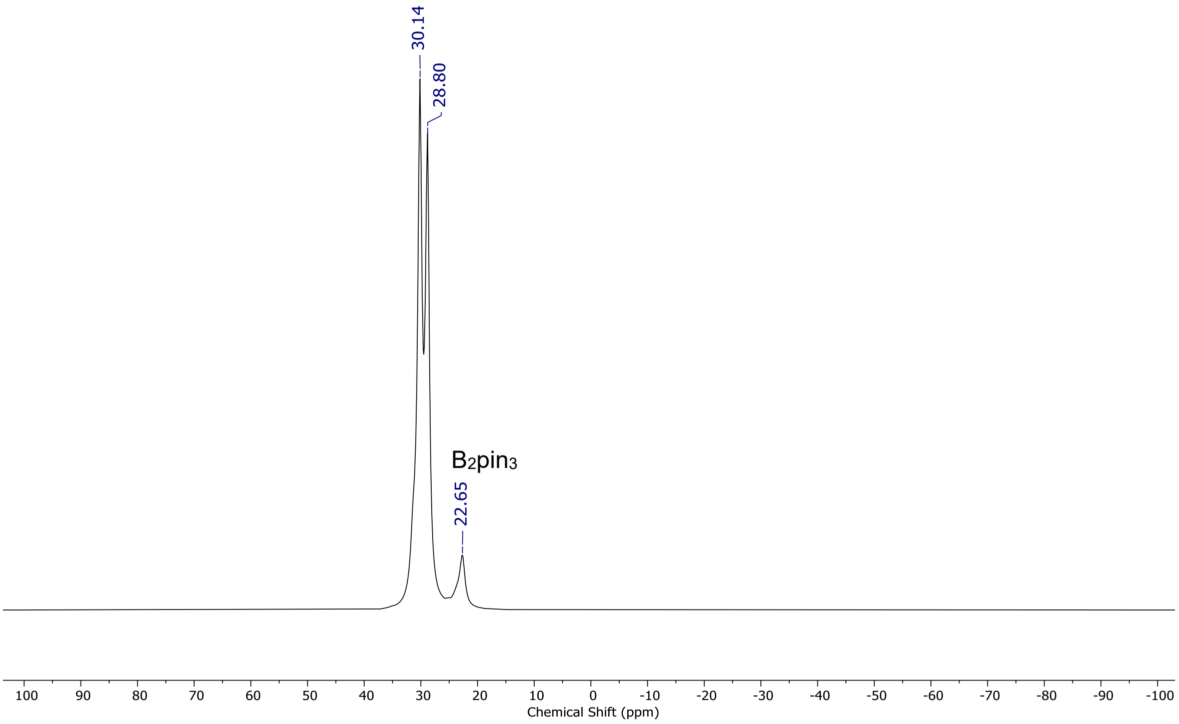

Fig. 60: ^{11}B NMR (160 MHz, h_4-tetrahydrofuran) of Co(acac)$_2$ and HBpin **3b**.

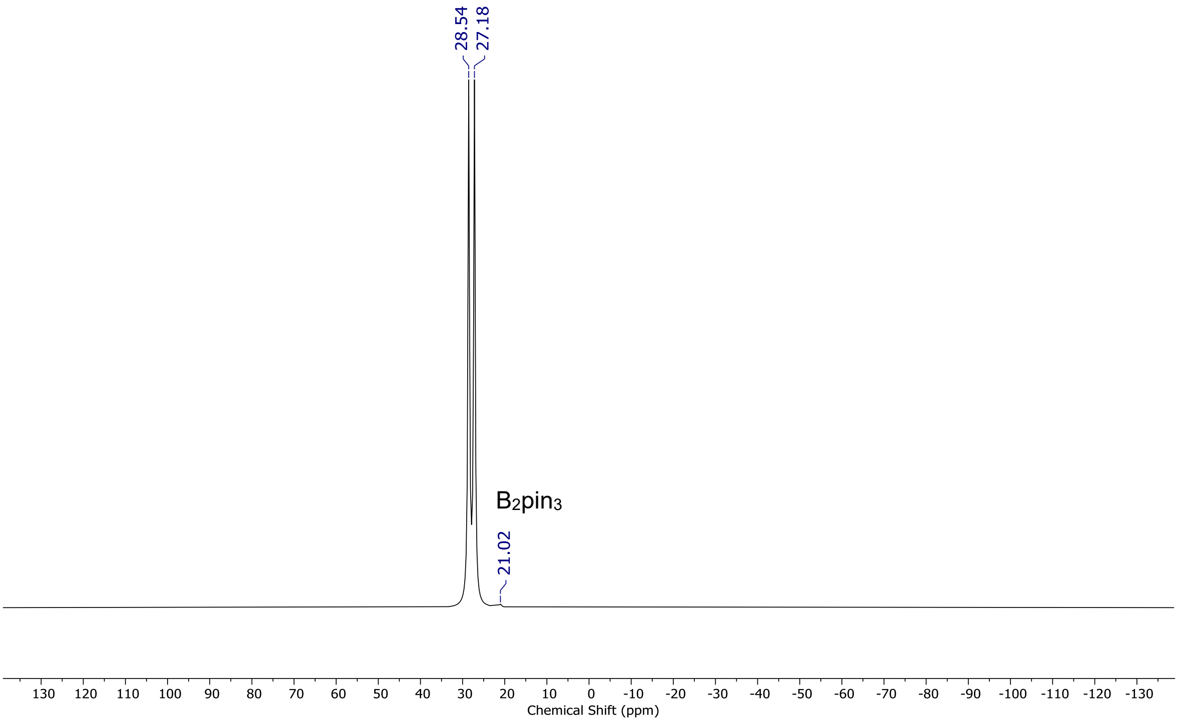

Fig. 61: ^{11}B NMR (160 MHz, h_4-tetrahydrofuran) of Xantphos and HBpin **3b**.

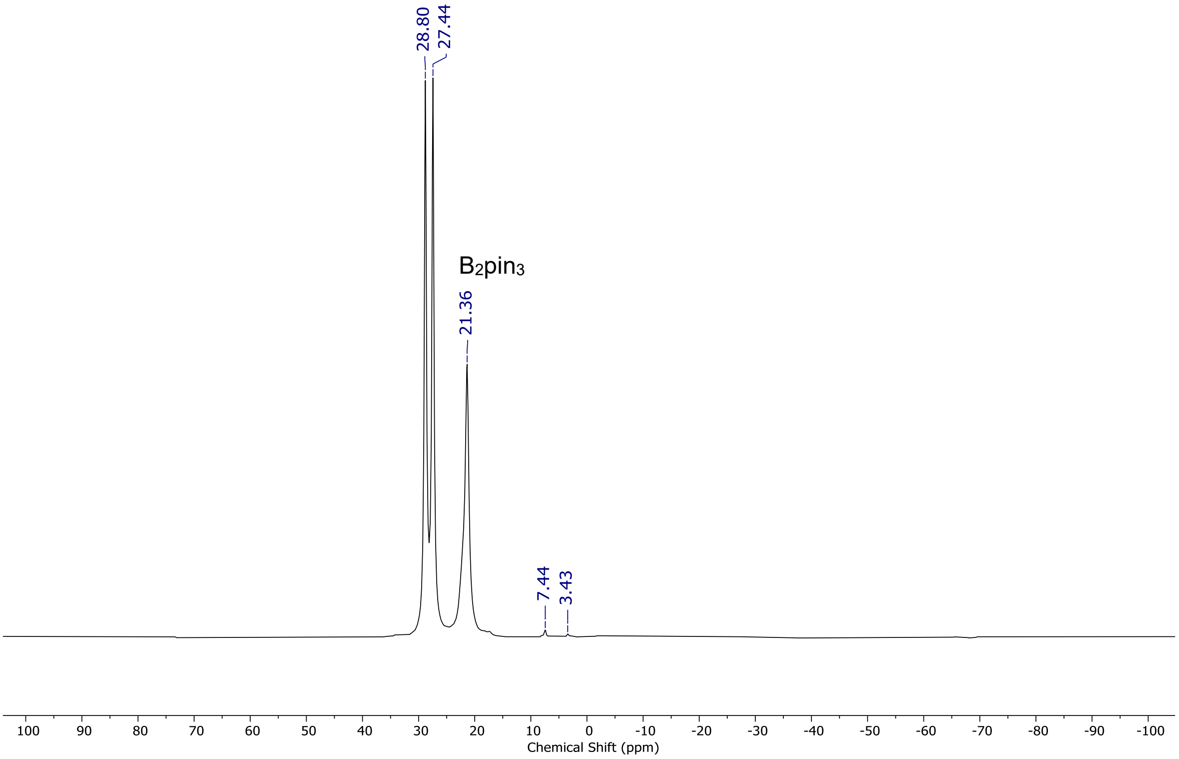

Fig. 62: ^{11}B NMR (160 MHz, h_4-tetrahydrofuran) of Co(acac)$_2$., Xantphos and HBpin **3b**.

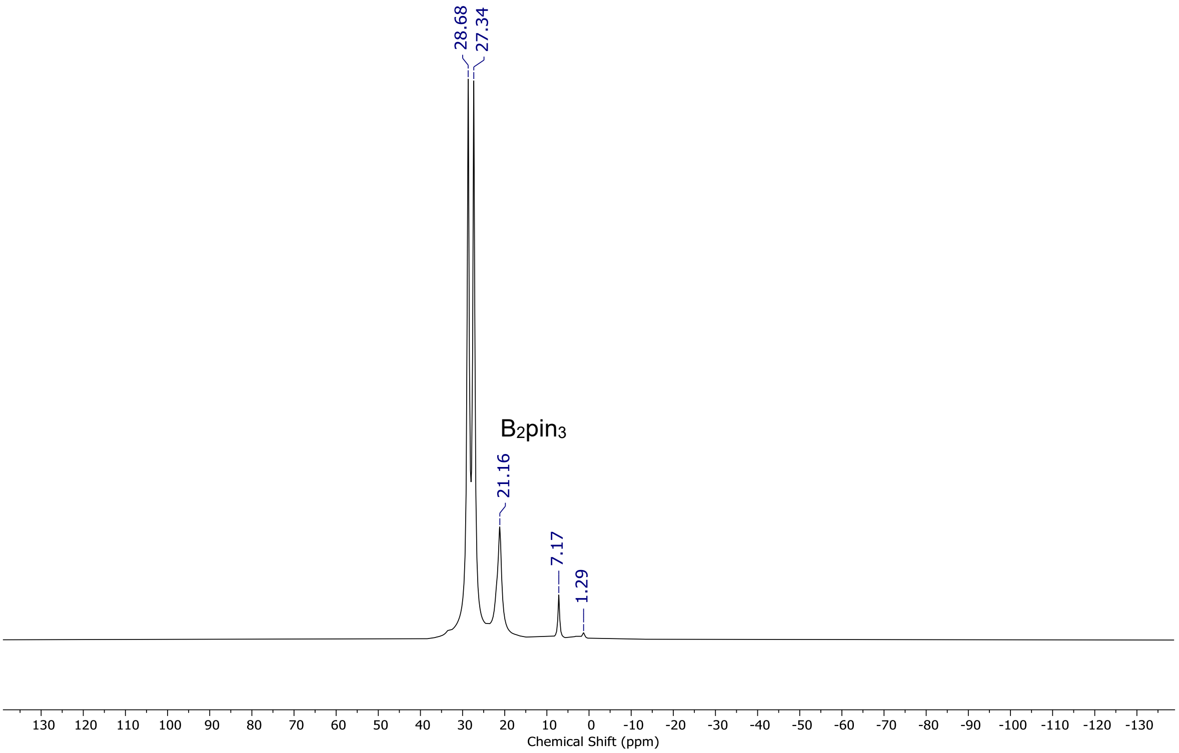

Fig. 63: ^{11}B NMR (160 MHz, h_8-toluene) of Ni(acac)$_2$ and HBpin **3b**.

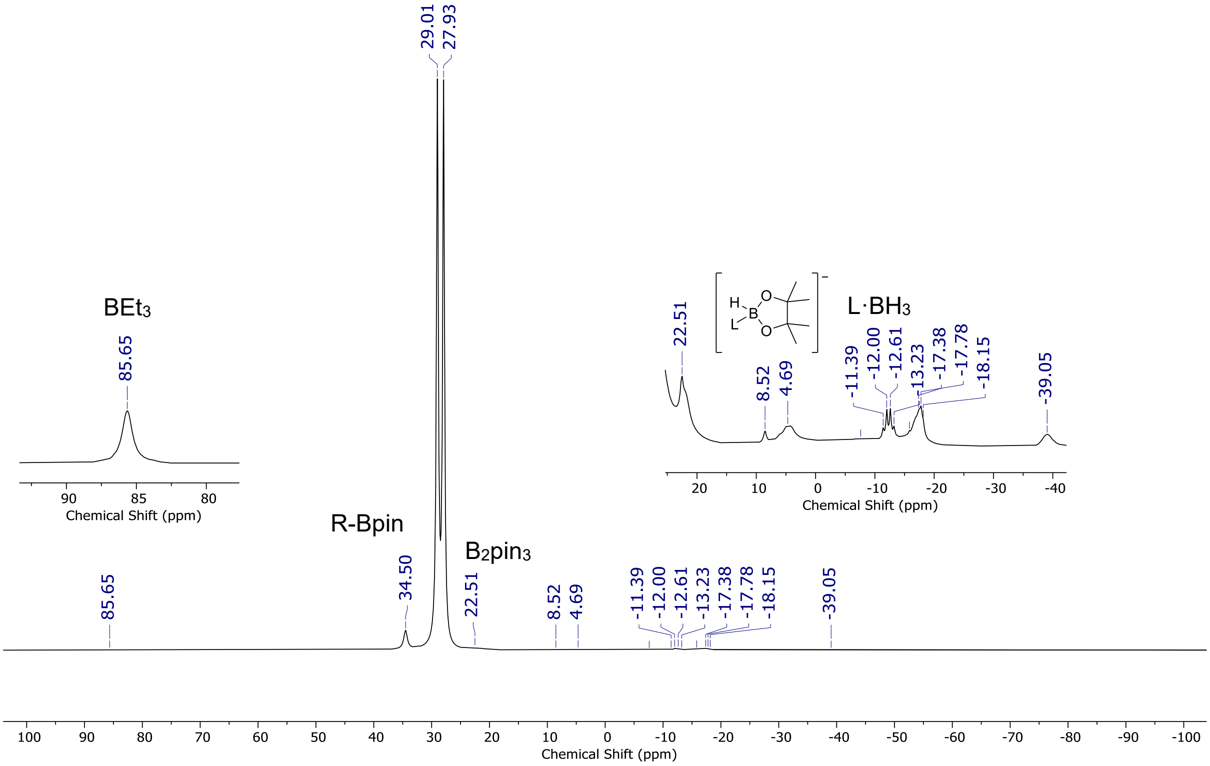

Fig. 64: ^{11}B NMR (160 MHz, h_8-toluene) of EtMgBr and HBpin **3b**.
